# Design of cross-reactive antigens with machine learning and high-throughput experimental evaluation

**DOI:** 10.3389/fbinf.2025.1580967

**Published:** 2025-07-16

**Authors:** Chelsy Chesterman, Thomas Desautels, Luz-Jeannette Sierra, Kathryn T. Arrildt, Adam Zemla, Edmond Y. Lau, Shivshankar Sundaram, Jason Laliberte, Lynn Chen, Aaron Ruby, Mark Mednikov, Sylvie Bertholet, Dong Yu, Kate Luisi, Enrico Malito, Corey P. Mallett, Matthew J. Bottomley, Robert A. van den Berg, Daniel Faissol

**Affiliations:** ^1^ GSK, Rockville, MD, United States; ^2^ Lawrence Livermore National Laboratory, Livermore, CA, United States

**Keywords:** AI, ML, protein engineering, vaccine, antigen, antibody, Neisseria meningitidis, protein structure

## Abstract

Selecting an optimal antigen is a crucial step in vaccine development, significantly influencing both the vaccine’s effectiveness and the breadth of protection it provides. High antigen sequence variability, as seen in pathogens like rhinovirus, HIV, influenza virus, complicates the design of a single cross-protective antigen. Consequently, vaccination with a single antigen molecule often confers protection against only a single variant. In this study, machine learning methods were applied to the design of factor H binding protein (fHbp), an antigen from the bacterial pathogen *Neisseria meningitidis*. The vast number of potential antigen mutants presents a significant challenge for improving fHbp antigenicity. Moreover, limited data on antigen-antibody binding in public databases constrains the training of machine learning models. To address these challenges, we used computational models to predict fHbp properties and machine learning was applied to select both the most promising and informative mutants using a Gaussian process (GP) model. These mutants were experimentally evaluated to both confirm promising leads and refine the machine learning model for future iterations. In our current model, mutants were designed that enabled the transfer of fHbp v1.1 specific conformational epitopes onto fHbp v3.28, while maintaining binding to overlapping cross-reactive epitopes. The top mutant identified underwent biophysical and x-ray crystallographic characterization to confirm that the overall structure of fHbp was maintained throughout this epitope engineering experiment. The integrated strategy presented here could form the basis of a next-generation, iterative antigen design platform, potentially accelerating the development of new broadly protective vaccines.

## Introduction

Advances in vaccine technologies, such as reverse vaccinology and structural vaccinology, have significantly enhanced our ability to develop vaccines for numerous diseases (Baden et al., 2021; Crank et al., 2019; Hsieh et al., 2020; Pizza et al., 2000; Polack et al., 2020; Rappuoli, 2001; Rappuoli et al., 2016). However, a significant remaining challenge in vaccinology is antigen sequence variation, where frequent mutations in infectious pathogens allow them to evade the immune system (McCulloch et al., 2017; Rappuoli, 2007). Consequently, developing broadly protective vaccines against highly mutating pathogens, such as influenza virus, HIV, rhinovirus, and meningococcus remains a difficult task (Rappuoli, 2007; Ng’uni et al., 2020).

*Neisseria meningitidis* factor H binding protein (fHbp) is a key component of vaccines that protect against meningitis and life-threatening sepsis caused by serogroup B meningococcus (Pace and Pollard, 2012; Rosenstein et al., 2001; Seib et al., 2015; Serruto et al., 2012; Zlotnick et al., 2015). Over 1,300 naturally occurring sequences of fHbp have been reported (Jolley and Maiden, 2010), classified into three variant groups (Bambini et al., 2009; Masignani et al., 2003; Murphy et al., 2009). Immunization with a single fHbp antigen typically induces an immune response specific to the administered variant group. Therefore, the licensed vaccines contain multiple antigens to ensure broad protection. This makes the fHbp antigen an ideal test case for the design of broadly protective antigens in variable pathogens. In addition, at least 20 crystal structures of fHbp variants and antibody-fHbp complexes are available in the protein data bank (Berman et al., 2016), providing a basis for rational design.

A significant challenge in rational antigen design is the vast search space. Each amino acid position has 20 different possibilities, resulting in an enormous combinatorial search space for a protein of 300 amino acids. This complexity makes it difficult for humans to navigate, so antigen design is typically driven by expert knowledge and focuses on the most promising protein domains. Advances in high-performance computing, simulation, and artificial intelligence (AI)/machine learning (ML) have paved the way for new approaches to rapidly design immunogens, including those not intuitive to human experts (Beede et al., 2020; Lalmuanawma et al., 2020; Ong et al., 2020). Unsupervised machine learning methods, including the use of protein sequence-based language models (Elnaggar et al., 2022; Prihoda et al., 2022; Verkuil et al., 2022), show substantial promise in developing proteins *in silico* that are fit, stable, or fold *in vitro*. These methods can also improve antibody binding behavior in a so-called “zero-shot” context (Hie et al., 2024). However, the need for large quantities of representative training data often makes these methods unsuitable for the scientific exploration of novel, idiosyncratic, or poorly characterized systems, such as vaccine antigen design (D'Amour et al., 2022). While substantial strides have been made, even the best ML structure prediction methods are uncertain or incorrect about many antibody-antigen co-structures, even when given high computational resources (Abramson et al., 2024), and structure-enabled ML antibody design remains challenging (Bennett et al., 2024). Conversely, there is substantial power and efficiency to be gained in iterative selection of designs using data obtained from measuring the quantity of interest in the system or protein of interest, including ML-assisted directed evolution approaches (Bachas et al., 2022; Shanehsazzadeh et al., 2024; Stanton et al., 2022; Yang et al., 2019).

Another strategy employed for large-scale *in silico* evaluation of protein-protein binding is physics-based calculations of binding energy. There are multiple simulation tools available to predict binding with differing levels of rigor. The fastest computational methods, such as FoldX (Schymkowitz et al., 2005) and STATIUM (DeBartolo et al., 2014) are the least rigorous. More elaborate methods, including Rosetta and molecular dynamics, are more rigorous and account for some flexibility in the protein backbone. While more rigorous approaches are expected to provide more accurate predictions, none of the available tools is completely accurate (Sirin et al., 2016).

In this work, we adopt a hybrid approach to manipulating antibody/antigen binding. This approach combines the advantages of physics-based simulations, machine learning, and experimentation. It has recently been shown that limited experimental data can be supplemented by molecular simulations to develop an ML tool for the prediction of protein stability (Jokinen et al., 2018) and that aggregation of a mixture of predictions can be a fruitful method for prioritizing mutations (Riahi et al., 2021). Another related application is redesigning prophylactic antibodies to counter viral escape (Desautels et al., 2024). Here, the motivating application is the creation of a vaccine antigen that would induce the production of broadly neutralizing antibodies *in vivo*, using *in vitro* binding to a panel of known, broadly neutralizing antibodies as a proxy for this desired shift in antigenicity. Collecting at large scale such binding data is still expensive and limited, as few data are collected in each system and each antigenic protein is distinct. Consequently, public data sets only contain thousands of relevant experimental observations (Sirin et al., 2016), while ML models typically employ millions or billions of datapoints. Simulation methods provide principled binding estimates, but effectively selecting antigen designs for subsequent *in vitro* testing requires a rigorous algorithmic framework for optimal performance.

Overall, this study establishes the capabilities required to implement machine learning as a primary component of an integrated strategy for vaccine antigen design. Specifically, this work attempts to expand the antigenicity of fHbp variant 3.28 (v3.28) by incorporating epitopes specific to fHbp variant 1.1 (v1.1), without compromising overlapping epitopes known to elicit cross-reactive antibodies. These two sequences have 60.2% identity and 75.7% similarity. Two phases of experimentation were conducted. In the set-up phase, the initial preparatory experiments focused on generating model-specific training data and confirmed that the target outcome was achievable. Next, the primary design campaign evaluated a Gaussian process (GP) model and Bayesian active learning method for candidate selection. The antigenicity of the candidate fHbp mutants was validated experimentally by measuring the affinity to a panel of antibodies (Bianchi et al., 2019; Lopez-Sagaseta et al., 2018; Malito et al., 2013; Malito et al., 2016; Veggi et al., 2020). Several fHbp v3.28 candidate antigens selected for experimental evaluation by the machine learning model gained binding to fHbp v1.1-specific antibodies, providing a proof of concept for further exploration of this approach.

## Results

### Preparatory experiments

**FIGURE 1:**
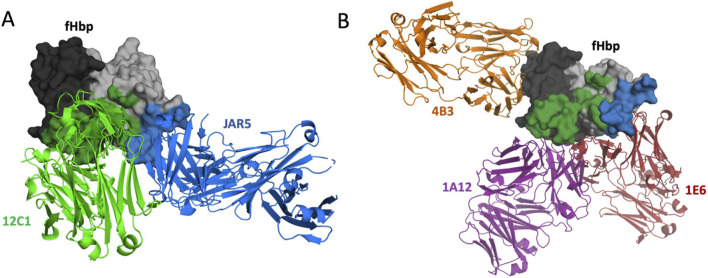
Illustration of the mAb binding locations on the surface of fHbp. Aligned overlays of independent crystal structures, each containing a single Fab antibody fragment bound to fHbp v1.1 or fHbp v3.28; PDB: 2YPV, 5T5F, 5O14, 6H2Y, and 6XZW (Riahi et al., 2021; Desautels et al., 2024; Bianchi et al., 2019; Lopez-Sagaseta et al., 2018; Malito et al., 2013). **(A)** Bound Fab structures of the fHbp v1.1 specific mAbs are depicted in green and blue. **(B)** Bound Fab structures of the cross-reactive mAbs 4B3, 1A12, and 1E6 are depicted in orange, purple, and red respectively. Residues in the epitopes of fHbp v1.1-specific mAbs JAR5 (blue) and 12C1 (green) are indicated on the fHbp surface.

**FIGURE 2:**
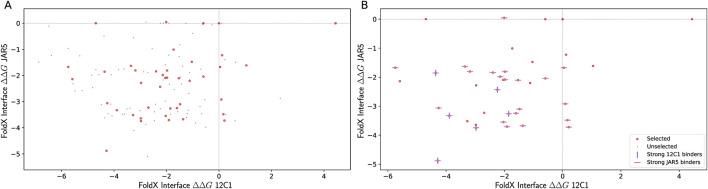
Round 1 summary of computational and experimental outcomes. **(A)** Interface ΔΔG calculated with FoldX for binding of fHbp v3.28-derived sequences to mAbs JAR5 and 12C1. ΔΔG values for binding to 1A12, 4B3, and 1E6 were also calculated (Supplementary Table S1). Of these sequences, and including v3.28, 41 were selected for production and are shown as red dots; sequences not selected for laboratory evaluation are shown as black dots. **(B)** For the selected sequences, experimentally validated binding is indicated with horizontal (pink, JAR5) or vertical (purple, 12C1) bars. Sequence m00006, which was selected as the starting sequence for Round 2, is at (−1.53, −2.11) in both panels.

The set-up phase focused on generating model-specific training data and confirming the feasibility of expanding the antigenicity of fHbp v3.28 to fHbp-specific antibodies. Three goals were considered in the design and selection of fHbp mutants in the set-up phase: (1) introduce substitutions into fHbp v3.28 or fHbp v1.1 to generate mutants with variable binding to two fHbp v1.1-specific monoclonal antibodies (mAbs) known as 12C1 and JAR5 (Figure 1), (2) select mutations in fHbp v3.28 that do not disrupt binding to cross-reactive mAbs 1A12, 4B3, and 1E6, and (3) determine the difficulty of introducing the 12C1 and JAR5 epitopes into fHbp v3.28 without disrupting binding to mAbs 1A12, 4B3, and 1E6. To accomplish these goals, residues within the 12C1 and JAR5 epitopes (as defined by their crystal structures with fHbp v1.1) (Malito et al., 2013; Malito et al., 2016) were identified and different combinations of these residues transferred from fHbp v1.1 into the fHbp v3.28 sequence (to test gain of binding) or vice-versa (to test loss of binding) (Figure 1). In locations where the amino acid residue is conserved between fHbp v1.1 and fHbp v3.28, a structurally compatible alternative residue for substitution at that location was selected by using StralSV, a computational tool that determines the probability of finding an amino acid given the local structure of the protein (Zemla et al., 2011).

In total, 131 mutants of fHbp v1.1 and v3.28 were proposed. The binding affinity for mAbs 12C1, JAR5, 1A12, 4B3, and 1E6 to each mutant was estimated computationally. Specifically, 10 homology models were generated for each mutant and two approaches from the FoldX package, ‘BuildModel’ and ‘AnalyseComplex,’ were used to determine the change in binding free energy (ΔΔG) for the interaction of each mutant with the mAbs (Supplementary Table S1; see also Materials and Methods). Forty-eight mutants, eight fHbp v1.1 derivatives and forty fHbp v3.28 derivatives, were selected for experimental evaluation considering binding predictions for all five mAbs and the stated goals (Figure 2).

In total, 50 fHbp proteins (48 mutants, plus fHbp v1.1 and fHbp v3.28) were produced *in vitro* on a small scale (4–12 mL) in *Escherichia coli* and evaluated for protein yield after one-step nickel affinity purification. From this group, 39 fHbp samples were successfully produced (final concentration >10 μg/mL), allowing the measurement of 195 antigen-antibody interactions (Supplementary Tables S2, S3) by biolayer interferometry (BLI). Affinity measurements (K_d_) fell between 750 pM and 5.5 nM for binding of all fHbp mutants to the cross-reactive mAb 4B3. This variation is expected given the accuracy of individual measurements by this technique. The consistent interaction with mAb 4B3, which recognizes a large discontinuous conformational epitope (Veggi et al., 2020), provided confidence that all fHbp mutants maintained the expected overall 3-dimensional fold. All mutants also bound the cross-reactive mAbs 1E6 and 1A12, though some mutants had an affinity reduced by up to 100-fold. Appreciable binding to mAb JAR5 was detected for 90% of the fHbp mutants produced, and 38% of the mutants also bound mAb12C1. Binding mutants were defined as having a measurable Octet K_d_ where the curve fit had an *R* ^2^ value of at least 0.8. The initial data for JAR5 and 12C1 binding to mutants includes 28 fHbp-mAb pairs with no measurable binding, four pairs with weak binding affinity (K_d_ > 50 nM), and 46 Ag-Ab pairs for which the binding of the fHbp mutant was similar to the Ag-Ab affinity observed for binding with fHbp v1.1. Mutants of fHbp v3.28 with the strongest binding to mAbs JAR5 and 12C1 (*n* = 2) had affinities equivalent to fHbp v1.1 (Table 1). Therefore, it was determined that the design problem selected for this project, gaining binding to mAbs JAR5 and 12C1 in fHbp v3.28 while maintaining binding to cross-reactive mAbs 1A12, 4B3, and 1E6, was achievable.

**TABLE 1:** Summary of measured affinities (K_d_) for binding of five mAbs to select fHbp mutant sequences determined by BLI.

		mAb affinity				
Antigen	Description	JAR5	12C1	1A12	1E6	4B3
fHbp v1.1	Wild type	200–700 pM	100–800 pM	Tight binding (no k_off_)	3–10 nM	1–5 nM
fHbp v3.28	Wild type	No binding	No binding	7–20 nM	2–4 nM	5–6 nM
m000019	Round 1 best mutant	254 pM	37 pM	Tight binding (no k_off_)	800 pM	2.4 nM
m000006	Round 2 starting sequence	Tight binding (no k_off_)	11.1 µM*	1.8 µM	870 pM	5.5 nM
m002416	Round 2 best mutant	631 pM	293 pM	544 pM	320 pM	3.8 nM

Ranges of typical affinities are provided for wild-type antigens when the fHbp protein was produced and mAb affinities measured in multiple independent experiments. Variations observed were consistent with the expected error of the high-throughput BLI assay. *No binding was measured in preliminary experiments. Weak binding was measured in the main design campaign after improving assay parameters. See also Supplementary Tables S2, S5.

Finally, it was observed during these preliminary experiments that mouse 12C1 mAb performed poorly in the BLI affinity assay, with specifically low signal/noise ratio even when binding affinity was strong. Therefore, we humanized the mouse JAR5 and 12C1 mAbs and observed significant improvements in assay performance (Table 1). This was attributed to the substitution of anti-mouse mAb biosensors (AMC) for anti-human mAb biosensors (AHC). While data quality was improved, the Ag-Ab affinity measured was the same. The humanized JAR5 and 12C1 mAb reagents were used for all subsequent work.

### Vaccine design pipeline integrating machine learning

The primary purpose of this work was to incorporate machine learning methods into our pipeline for vaccine design and selection. In the main experiment for this project, a machine learning method was implemented to select a diverse pool of fHbp mutant sequences after computational prediction of Ag-Ab binding. The design was focused on the 12C1 epitope (more difficult design problem than JAR5 epitope) and implemented a mutant design strategy that reduced the need for prior knowledge of fHbp v1.1. All mutants were made in fHbp m000006, a mutant of fHbp v3.28 identified in the preliminary work, that had good protein expression (88% yield when compared with wild-type fHbp v3.28). fHbp m000006 is primarily fHbp v3.28 with 51 residues mutated to their counterparts in fHbp v1.1. During the preliminary experiments, this mutant bound mAb JAR5 and did not bind mAb 12C1. The objective for the machine learning campaign was to computationally design and select mutants of fHbp m000006 that bound mAb 12C1 and improved binding to mAb 1A12, which shares an overlapping epitope with mAb 12C1.

**FIGURE 3:**
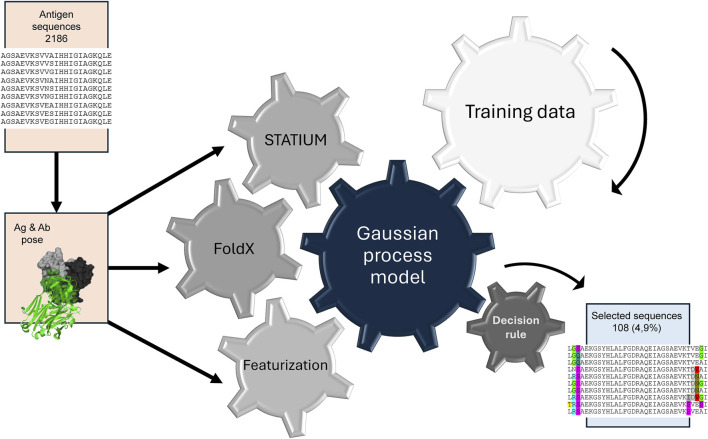
Overview of the computational strategy for selection of diverse candidates with the GP model. All candidates within the defined search space are computationally modelled and multiple binding parameters are predicted using established biophysical tools, including STATIUM and FoldX. This data is combined in a machine learning model and the selection of candidates is driven by the training data provided. This allows the selection of a small set of candidates for experimental evaluation.

**FIGURE 4:**
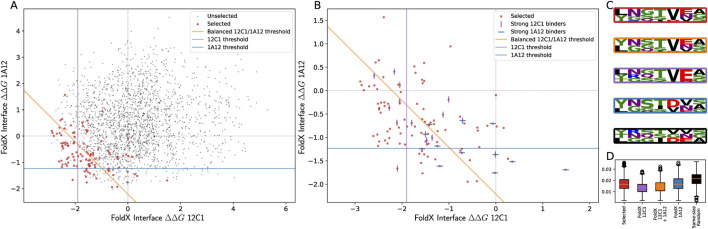
Sequences selected by the Gaussian process model for experimental evaluation. **(A)** Scatter plot of FoldX interface ΔΔG values (AnalyseComplex) for all 2,186 mutants evaluated computationally. The 108 mutants selected using the machine learning model and the decision rule are highlighted in red. Lines superimposed on the figure show effective selection thresholds in FoldX values corresponding to alternative sets of 108 mutants. If the 108 sequences with the best FoldX-predicted ΔΔG in binding 1A12 had been selected, points below the blue line fall in this set. Similarly, for if FoldX predicted binding of 12C1 is the only criterion, points left of the purple line are the resulting set, and for the equal sum of the two ΔΔGs for 1A12 and 12C1, points below and left of the orange line would constitute the resulting set. **(B)** The selected 108 points were tested in BLI. Among these, the best binders for 12C1 and 1A12 are marked, using cyan vertical and blue horizontal bars, respectively. A number of selected, strongly binding sequences do not lie in any of the sets constructed *post hoc* on the basis of FoldX binding predictions. **(C)** For each of group of sequences, sequence logos showing positions 221-223 and 249-252 are framed by the corresponding color. The sequence logo framed in black is all 2,187 mutants, including the parental sequence. **(D)** Pairwise distances among the 108 sequences in each selected or comparison set, using Blosum62. The black set is a size-matched, randomly selected set of 108 sequences from all 2,186 mutants considered, giving a fair comparison of intra-set sequence distances. Boxes show first quartile, median, and third quartile, while whiskers are 1.5 times the interquartile range or to the most extreme datum, whichever is narrower. Using the GP model, the decision rule traded off predicted binding performance for greater diversity in the selected set.

**FIGURE 5:**
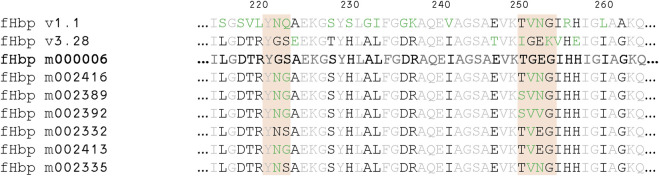
Alignment of the fHbp v3.28 mutant sequences with the highest affinity for mAb 12C1 and 1A12. The starting sequence selected for the machine learning design test, fHbp m000006, is shown in bold and deviations from this sequence are shown in green font. Residues identical in all sequences are in light grey. Locations allowed to mutate are highlighted.

Seven residue locations were selected for mutation, specifically in the region where the 12C1 and 1A12 epitopes overlap on the fHbp v3.28 surface, producing a design search space of 20^7^ (20 amino acids possible for each of seven locations). The search space was narrowed by selecting the top three structurally compatible amino acid substitutions using StralSV (Table 2) (Zemla et al., 2011). This resulted in a pool of 2,186 (3^7^-1) mutated sequences. This search space was probed with a hybrid computational approach as outlined conceptually in Figure 3. Calculations were performed using FoldX and STATIUM to calculate the ΔΔG for each mutant when binding all five mAbs (Supplementary Table S4). Machine learning was used to evaluate this data. Specifically, this work employed multi-objective Bayesian optimization and a GP model trained with published data from the AB-Bind database (Sirin et al., 2016) and project-specific data points collected in the preliminary experiments. Finally, a batch upper confidence bound decision rule (Desautels et al., 2014) was used to iteratively select 108 sequences, or 4.9% of the candidates for experimental evaluation (Figure 4). This decision rule ensured exploration (broad acquisition of information) with exploitation (selected mutants with the best predictors), while balancing the binding of fHbp mutants to mAbs 12C1 and 1A12 simultaneously. As constructed, the decision rule favored sequences with the best FoldX predictions, and the most important contribution of the GP model was to ensure diversity in the selected sequences for experimental evaluation. This stands in contrast to selection methods based on the output of individual computational predictors, such as FoldX alone, which might result in homogeneous, narrow groups of mutants (see Figure 5) that could fall victim to potential biases introduced by these tools.

**TABLE 2:** Comparison of key sequences.

Residue Location	221	222	223	249	250	251	252
fHbp v1.1	Y	N	Q	T	V	N	G
fHbp v3.28	Y	G	S	I	G	E	K
m000006	Y	G	S	T	G	E	G
Allowed mutations based on StralSV	L T Y	R G N	S G Q	S T I	D V G	V N E	A G S
Consensus of top binders	Y	N	G/S	T/S	V	Any	G

Focused alignment of amino acid residues. Includes residues from fHbp v1.1, fHbp v3.28, the starting sequence for design (m000006), amino acids selected by StralSV, and a consensus sequence derived from the top mutations after experimental evaluation.

Experimental evaluation of binding affinity was carried out for a selected batch of 102 candidate sequences, 83 of which could be produced in small scale *E. coli* cultures (8 mL) with sufficient yields (5%–50% yield compared with fHbp v3.28 wild type). Affinity measurements for the binding of 12C1, 1A12, and 1E6 were performed for all obtained mutants (Supplementary Table S5). Re-evaluation of the starting mutant, fHbp m000006, as a control in the main design campaign revealed unexpected weak binding of fHbp m000006 to mAb 12C1. This was attributed to improvements in the experimental assay. Therefore, final evaluation of the mutants focused on simultaneous improvement of binding to mAbs 12C1 and 1A12. All evaluated mutants bound mAb 1E6 with an affinity equivalent to the starting sequence (within 2-fold), or stronger, confirming that all fHbp mutants were folded. As expected, variation was observed among the binding of mutants to mAbs 12C1 and 1A12. In total, 40 mutants selected by machine learning had measurable affinity to mAb 12C1 and mAb 1A12. From this set, 17 fHbp mutants with the highest binding affinities were selected and it was confirmed that these samples also bound mAb 4B3 and mAb JAR5 with the expected affinity. Finally, six mutants were identified that bound all five antibodies with affinities similar to those obtained for fHbp v1.1 (affinity for mAbs 1A12 and 12C1 both stronger than 5 nM). When the respective sequences were aligned, they revealed a high degree of homology within the target mutation sites to fHbp v1.1 (Figure 5; Table 2) and a consensus sequence was derived (Table 2).

In total, 12 mutants, or 0.5% of the mutants in the decision set of 2,186 possibilities, conformed to this consensus sequence. The 108 mutants selected for experimental evaluation represented 4.9% of the total possibilities and included seven, or 58.3% of the potential fHbp candidates that matched the consensus sequence, a 12-fold enrichment. Only one experimentally evaluated mutant that conformed to this consensus showed weaker binding, namely, fHbp m002419, with an affinity to mAb 1A12 being 10 nM. Overall, the machine learning methods implemented here selected an enriched set of mutants for experimental testing, leading to multiple candidates capable of binding mAbs 12C1 and 1A12 with high affinity (stronger than 5 nM).

### Biophysical characterization of a top mutant confirms protein integrity and antigenic design

The computational and experimental evaluation of fHbp mutants thus far focused solely on antigenicity (antibody binding) and did not confirm protein stability/integrity/folding beyond the inclusion of cross-reactive mAbs in the testing panel. The mutant from the ML design campaign with the highest measured affinities for 12C1 and 1A12, fHbp m002416, was produced in sufficient quantity to perform further biophysical characterization. Purified fHbp m002416 was monodisperse and monomeric as determined by size-exclusion chromatography (Supplementary Figure S1). The thermal stability of fHbp v1.1, fHbp v3.28, and fHbp m002416 was compared using differential scanning fluorimetry. As shown previously, fHbp v1.1 showed greater thermostability in the N-terminal domain (Tm1) relative to fHbp v3.28 (Malito et al., 2013; Johnson et al., 2012). By comparison, fHbp m002416 showed a reduction in thermostability, most significantly in the N-terminal domain, relative to both fHbp v1.1 and fHbp v3.28, suggesting a significant impact from the introduced mutations (Table 3).

**TABLE 3:** Thermostability of fHbp samples determined by differential scanning fluorimetry.

Sample	Melting temperatures	
	Tm1	Tm2
fHbp v1.1	64.4	86.2
fHbp v3.28	59.0	84.1
fHbp m002416	47.2	82.1

Measurements were performed in duplicate and the average is reported.

**FIGURE 6:**
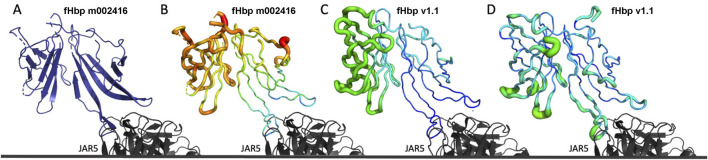
Crystal structure of fHbp m002416. **(A)** Crystal structure of fHbp m002416 (blue) crystallized with JAR5 (black) PDB: 2YPV. **(B)** Crystal structure of fHbp m002416 bound to JAR5 (black) with fHbp m002416 colored by b-factors. **(C)** Crystal structure of fHbp v1.1 in complex with JAR5 (black) in space group C2221, fHbp v1.1 colored by b-factor. **(D)** Crystal structure of fHbp v1.1 in complex with JAR5 (black) in space group I4122, fHbp colored by b-factor. B-factor scale from high (red) to low (blue) and standardized across panels **(B**–**D)**.

To further explore the impact of mutations in fHbp m002416, a crystal structure was determined for this protein in complex with the Fab fragment of mAb JAR5 (Figure 6). JAR5 Fab was crucial for the crystallization of fHbp m002416, which did not crystallize in isolation. The JAR5 epitope/paratope interface adopts the same conformation as seen previously in the structure of JAR5 bound to fHbp v1.1 (Malito et al., 2016). The overall fold and domain structure reproduced the expected backbone for fHbp, including the 12C1 epitope where mutations were introduced. While >70% of the amino acid residues in fHbp m002416 were visible within the electron density, a section of the C-terminal domain could not be modelled due to flexibility and high local b-factors. When crystallized in space group C2, the solvent content of the crystal was 37%, the C-terminal domain of fHbp participated in few crystal contacts, and the disordered region was entirely exposed to the solvent channels. In contrast, crystals of fHbp v1.1 bound to JAR5 were previously grown in space groups C222_1_ (Malito et al., 2016) or I4_1_22, where the full C-terminal beta-barrel was modelled, despite above average b-factors relative to the N-terminal domain. Therefore, while fHbp m002416 did not maintain the thermostability of natural fHbp variants, it did maintain the overall fold of fHbp proteins while increasing the cross-reactivity to the tested panel of relevant antibodies. These results emphasized the importance of optimizing the computation-designed antigens for multiple parameters, including both antigenicity and stability, in future campaigns. Crystallographic statistics for new structures presented herein are summarized in Supplementary Table S6.

## Discussion

The type of interaction between antigen and antibody is distinct from general protein-protein interactions, where both binding partners have co-evolved over time (Akbar et al., 2021; Graves et al., 2020). Therefore, it is important to develop specific datasets and tools for predicting and optimizing antibody-antigen interactions. Protective epitopes can often be clustered close together (Neu et al., 2016) and few tools are available for optimizing the binding of antigens to multiple antibodies simultaneously. Machine learning methods employing GP-models have already been successfully applied to predict various protein properties, including thermostability (Jokinen et al., 2018; Pires et al., 2014; Romero et al., 2013), substrates for enzymatic reactions (Mellor et al., 2016), fluorescence (Saito et al., 2018), membrane localization, and peptide binding to MHC complexes (Ren et al., 2011). This work evaluated an integrated approach, incorporating both biophysical calculations of Ag-Ab affinity and machine learning strategies to optimize binding to multiple mAbs with overlapping binding sites.

The AB-bind database provided a limited number of datapoints (1,109) for generating a training dataset for immediate implementation of machine learning. Additionally, 45% of mutants in the AB-Bind database contain only alanine substitutions, and 37% represent a single point mutation to alanine (Sirin et al., 2016). Active ML strategies have shown small amounts of problem-specific data can significantly improve the performance of ML algorithms, especially when applied iteratively (Brochu et al., 2010; Liu, 2004; Shahriari et al., 2016). Therefore, preliminary experiments were designed to supplement the AB-bind data with fHbp-mAb datapoints, including a diversity of amino acid mutations and multi-residue mutations.

The primary design campaign focused on the 12C1 epitope and its overlap with the 1A12 epitope. While the preliminary experiments demonstrated that fHbp v3.28 can be mutated to bind fHbp v1.1-specific mAbs 12C1 and JAR5, the manual design strategy relied heavily on knowledge of the fHbp v1.1 sequence. By incorporating ML, the goal was to significantly reduce our reliance on information from fHbp v1.1. Additionally, the co-evolution of overlapping epitopes is a key point in vaccine design, making the interplay between 1A12 and 12C1 binding of specific interest.

All residue locations shared between the 12C1 and 1A12 epitopes were allowed to mutate, and the number of residues allowed in each position was down-selected. To provide an unbiased method for determining the search space, the algorithm StralSV (Zemla et al., 2011) was used to suggest the most compatible residues based on local structure. The top three residues for each location included known residues from fHbp v3.28, fHbp v1.1, and one to two additional residues. Thus, it was apparent that the known solution, all fHbp v1.1 residues in these locations, would be within the search space. After experimentally evaluating 108 computationally selected sequences, a consensus motif was determined based on the top six successful mutants. This sequence conflicted with the fHbp v1.1 sequence in only one position, residue 223, where a glycine or serine was preferred over glutamine. This substitution in the top candidates did not improve the binding of fHbp to either mAbs 12C1 or 1A12 over fHbp v1.1.

ML models provide a measure of the informational relationships and dependencies among a collection of many variant sequences. This allows the construction of batches of simultaneous experiments that strategically trade off exploitation of knowledge (prediction of strong performance) with the acquisition of valuable, non-redundant information on the overall design space. Balancing these objectives can result in practical gains in the quality of the selected sequences when taken as a group, including discovering effective designs not in the top tier of any ranking directly derived from computational tools (see Figure 5A), greater sequence diversity, and the balancing of competing design goals (Figures 5B,C). In this case, the search scope after down selection by StralSV was too narrow to allow the algorithm to propose a non-intuitive solution. Within the space allowed, the algorithm funneled toward the known fHbp v1.1 solution. To expand on this work in the future and find potentially novel solutions, the amount of down selection performed by StralSV will need to be reduced.

While antigenicity and binding affinity are key properties of antigens, developability and antigen stability are also crucial for developing an effective vaccine. Here, 18% of sequences selected by the ML algorithm were not successfully produced, reducing the efficiency of experimental evaluation. While the high-throughput production methods employed do not pin-point specific failures, the most likely issues include inadequate protein expression, protein aggregation, or reduced stability (Tokuriki and Tawfik, 2009). Given that the computational model only considers binding affinity, there was concern that stability and protein folding in successful designs had also been impacted. To further explore, the highest affinity binder, fHbp m002416, was selected for biophysical characterization. A crystal structure of this ML-derived mutant confirmed the expected 3D fold of the fHbp protein backbone. Interestingly, while this mutant folded correctly and had high affinity for both antibody targets, its thermostability was reduced. Therefore, an optimal ML strategy for vaccine antigen design must balance improved antigenicity with other protein properties, including protein expression and stability. This phenomenon has also been observed in the development of monoclonal antibody therapies and small molecule therapeutics using ML methods (Schneider et al., 2020).

Given the rapid advance of vaccine delivery platforms, including recent breakthroughs in using mRNA-encoded antigens, selecting and designing appropriate molecules is poised to become a rate-limiting step in vaccine development and future pandemic responses. The application of computational algorithms has massive potential to reduce the number of wet-lab experiments and the time required for this stage of development. Moving forward, an active ML approach that iterates between computational simulations and targeted wet-lab experimentation performed in a rapid high-throughput manner is expected to provide the most efficient tool for vaccine antigen development. Our work established the core capabilities required to develop this technology further and tackle more challenging design problems.

## Materials and methods

### Computational evaluation and selection of mutants for preparatory experiments

X-ray crystallographic structures of fHbp v1.1 bound to five specific antibodies, namely, 12C1, JAR5, 1A12, 1E6, 4B3, are available in the Protein Data Bank as 2YPV, 5T5F, 5O14, 6H2Y, and 6XZW (Bianchi et al., 2019; Lopez-Sagaseta et al., 2018; Malito et al., 2013; Malito et al., 2016; Veggi et al., 2020). All antibodies bind fHbp v1.1; antibodies 1A12, 1E6, and 4B3 also bind fHbp v3.28. Five homology models were created using the AS2TS system (Zemla et al., 2005) for fHbp v3.28 in complex with each antibody based on the available crystal structures. Models of fHbp v3.28 in complex with the antibody panel were used to define two putative epitopes each, including residues with at least one atom from fHbp within a distance ≤5 and/or ≤7 Å from the respective antibody (Supplementary Figure S2). Non-conserved amino acid locations within the proposed 12C1 and JAR5 epitopes were mutated to contain fHbp v1.1 residues. Overlapping positions were defined as those that are included in more than one epitope. Residues in overlapping regions were varied within the mutant panel to include residues present in fHbp v1.1, fHbp v3.28, or those suggested as conservative mutations by StralSV (Zemla et al., 2011). StralSV was used to select an alternative residue substitution at the positions where V1 and V3 had the same residue. The final mutants scored well according to ΔΔG assessment, and as anticipated showed binding to JAR5 experimentally confirmed. Sequences for all mutants evaluated are listed in Supplementary Document S1.

Five homology models were created using AS2TS (Zemla et al., 2005) for each sequence in the initial pool of candidates (5 × 131 = 655 total) to generate a pose in complex with each of the five antibodies. Each model was energy-minimized using molecular dynamics (MD) in GROMACS using the steepest descents method (Abraham et al., 2015). Both the energy-minimized, and original homology models were used in future calculations, for a total of 10 models per fHbp antigen sequence. Two approaches from the FoldX package were used to determine the change in binding free energy (ΔΔG); ‘BuildModel’ - “FOLDX total energies before and after introducing mutations” and ‘AnalyseComplex’ - “FOLDX energies in interfaces” (Schymkowitz et al., 2005). From this initial set of 131 mutants, 48 mutants were selected based on the best scores in all evaluated complexes and agreement in the two approaches for calculating ΔΔG values. All ΔΔG values calculated in this round are included in Supplementary Table S1.

### Computational evaluation and selection of mutants using a GP model

From the training data, mutant m000006 was selected as the starting fHbp sequence. Seven residues from a region of the 12C1 epitope that overlaps with the 1A12 epitope were identified. StralSV was used to select the top three residue substitutions that would be compatible with these locations (Zemla et al., 2011). This yielded 2,186 (3^7, minus the unmutated m000006 sequence) sequences, and homology models were generated in AS2TS (Zemla et al., 2005) for each of them. FoldX (Schymkowitz et al., 2005) and STATIUM (DeBartolo et al., 2014) calculations were performed for each candidate to determine the change in binding free energy (ΔΔG) relatively to the starting sequence. This yielded 2,186 (3^7, minus the unmutated m000006 sequence) sequences (Supplementary Document S2) and homology models were generated in AS2TS (Zemla et al., 2005) for each of them. FoldX (Schymkowitz et al., 2005) and STATIUM (DeBartolo et al., 2014) calculations were performed for each candidate to determine the change in binding free energy (ΔΔG) relative to the starting sequence (Supplementary Table S4).

The relationships between likely experimental outcomes were modeled via a GP (Rasmussen et al., 2004). To represent mutant antigens to the GP model and make predictions about the effects of these mutations, a feature vector was constructed as follows: The contacts or potential contacts in these interfaces were identified by *α*-*α* distances of 10 Å or less. Each residue in such an interaction was assigned a chemical class and a size class (Supplementary Table S7); each identified interaction was then added to a tally of how many interactions exhibit particular chemical types (e.g., an aliphatic/aromatic interaction) and size types (e.g., a small/very large interaction). This set of all interaction features, for both the pre- and post-mutation versions of the antigen, was assembled into a feature vector. The outputs of the STATIUM and FoldX models were concatenated onto this feature vector and the whole feature vector was passed to the GP. The GP applies a normalizing feature transformation and Matérn kernel function to determine the similarity of any two such sequences’ mutational effects on the binding free energy. The model was implemented in Python in scikit-learn (Pedregosa et al., 2011). It was trained on the AB-Bind data set (Sirin et al., 2016) and antigen-specific results from round one experiments. For each interaction, a score is calculated, which is the model’s predicted mean, the model’s predicted variance, and a weighted combination of the FoldX and STATIUM scores. Because antigens must bind several target antibodies, separate predictions and separate scores were calculated for each of the target antibodies. The final score function was a sum over target antibodies:

$$g_{t}\left(x\right)=\sum_{i}\alpha_{i}\mu_{i|t-1}\left(x\right)+\beta_{i}\sigma_{i|t-1}\left(x\right)+\gamma \cdot S_{i}\left(x\right)$$

Where x was the antigen under consideration; $\mu_{i|t-1}$ and $\sigma_{i|t-1}$ were the predicted (“posterior”) mean and standard deviation of the GP model for this antigen and the i th target antibody, given selections 1 to t-1; $S_{i}$ was the set of simulation results (FoldX and STATIUM); and α,β,γ were weighting coefficients, respectively scalar-, scalar-, and vector-valued. We then selected the maximizer of $g\left(x\right)$ , i.e.,

$$x_{t}=\underset{x\in D_{t}}{\mathrm{argmax}}g_{t}\left(x\right)$$

Where $D_{t}$ was the current decision set. Note that because desirable values for μ and S were negative (i.e., mutation is desired to make binding free energy more negative), α and γ were negative.

A batch upper confidence bound decision rule (Desautels et al., 2014) was adapted and applied to the posterior distribution to iteratively select the batch of 108 sequences from the decision set of 2,186. This decision rule balances exploration (broad acquisition of information) with exploitation (selecting sequences that are predicted to be promising in terms of free energy), while balancing the multiple target antibodies.

### High-throughput cloning and sequencing

The fHbp mutant amino acid sequences were reverse-translated and codon-optimized for *E. coli* K12 expression using the Geneious R10 software (Version 10.1.3, Biomatters Ltd., San Diego, CA). Target sequences were synthesized as gBlocks (Integrated DNA technologies, IDT, Iowa, United States) and cloned into plasmid pBR322. Linearized plasmid was produced by PCR using Q5 Hot-start High-Fidelity DNA Polymerase (New England Biolabs) and site-specific primers (IDT), following the manufacturer’s protocol. Clones were assembled using NEBuilder HiFi DNA Assembly (E5520S, New England BioLabs) to perform Gibson Assembly in an automated 96-well plate format on a customized Tecan Fluent platform. This platform was used to resuspend dried DNA reagents, combine reaction components at the appropriate ratios, heat/cool the plate as required, transform competent bacteria, spread and incubate bacterial plates, and pick colonies for inoculation into LB media. New plasmid DNA constructs were isolated from bacterial culture with the MagJET Plasmid DNA Kit (ThermoFisher Scientific) automated in a 96-well format on a Kingfisher Flex (ThermoFisher Scientific). Sequence confirmation was performed by Sanger sequencing (Genewiz).

### Production of antibody reagents

Plasmids for the expression of mAb 4B3, 1E6, 1A12, JAR5, and 12C1 have been described previously (Bianchi et al., 2019; Lopez-Sagaseta et al., 2018; Malito et al., 2013; Malito et al., 2016; Veggi et al., 2020). The human mAb 4B3, 1E6, and 1A12 and mouse antibodies JAR5 and 12C1 were prepared as previously reported (Veggi et al., 2020). In brief, plasmid DNA was prepared by maxi-prep and transiently transfected into Expi293 cell using an ExpiFectamine kit (ThermoFisher). Culture supernatant was harvest after a 4–5-day growth in a shake-flask culture at 37°C and 8% CO_2_. Antibodies were purified from the harvested supernatant by affinity chromatography (HiTrap MabSelect SuRe, Cytiva) followed by size-exclusion chromatography (Superdex 200 16/600, Cytiva). Antibodies were eluted from the affinity column with 0.1 M sodium citrate pH 3.0, which was immediately neutralized with 1 M Tris pH 9.0 (5:1 ratio). Buffer was exchanged into the storage buffer, 20 mM HEPES pH 7.0, 150 mM NaCl, during size-exclusion chromatography. The mouse antibodies had sub-optimal performance during initial binding studies (specifically low signal/noise ratio). Therefore, the variable domains from the mouse JAR5 and 12C1 mAb were subcloned into a human IgG framework containing the crystallizable domains (CH1, CH2, CH3, CL1). These chimeric antibodies, hJAR5 and h12C1, were produced using the same protocol and used for evaluating the mutants selected by the GP model.

Fab fragments for mouse JAR5 were sub-cloned from the original mouse vectors and produced in Expi293 cells using the same overall protocol as the mAb reagents. Culture supernatant was concentrated and diafiltered into Tris saline buffer (50 mM Tris pH 8.0, 150 mM NaCl) prior to purification. Fab fragments contained a Streptavidin tag fused to the C-terminus of the heavy chain which enabled purification by affinity chromatography (StrepTrap, Cytiva). Protein was eluted in 100 mM Tris pH 8.0, 150 mM NaCl, 1 mM EDTA, 2.5 mM desthiobiotin and further purified by size-exclusion chromatography (Superdex 200 Increase 10/300 GL, Cytiva) in 25 mM Tris pH 8.0, 150 mM NaCl. All antibody samples were concentrated using Millipore centrifugal filters. Antibody reagents were stored frozen at −80°C prior to characterization.

### Production of fHbp reagents

Plasmids for the expression of wild-type fHbp variants 1.1 and 3.28 with a C-terminal 6-His fusion tag were reported previously (Masignani et al., 2003; Giuliani et al., 2005). Protein sample references and protein samples for X-ray crystallography were produced in low throughput on a large scale. Chemically competent *E. coli* BL21(DE3) star cells (ThermoFisher) were transformed with the respective fHbp plasmid and protein was expressed during growth in up to 1 L of Terrific Broth (TB) media containing 100 μg/mL carbenicillin. Cultures were incubated at 37°C for 16 h prior to harvest. Cells were lysed by sonication and insoluble debris was removed by centrifugation. Soluble protein was purified by nickel-affinity chromatography (HisTrap FF, Cytiva). Protein was eluted with a linear gradient of 20–250 mM imidazole in 50 mM Tris pH 8.0, 300 mM NaCl. Further purification was performed by size-exclusion chromatography (Superdex 200 Increase 10/300 GL, Cytiva) using 25 mM Tris-HCL pH 8.0 and 150 mM NaCl as the elution buffer. Protein samples were concentrated (Millipore-Sigma) and protein concentration was determined using optical density at 280 nm before storage at −80°C.

Mutant fHbp sequences were produced in parallel using a high-throughput approach. Protein was expressed using 4 mL cultures of TB media with 100 μg/mL carbenicillin in 24 deep-well plates shaken at 37°C. Harvested cells were lysed with BugBuster Master Mix reagent (Millipore Sigma). Mutant fHbp samples were purified using one-step nickel-affinity chromatography using the MagneHis protein purification system (Promega) and automated with a Kingfisher Flex (ThermoFisher). Proteins were eluted in 50 mM Tris pH 8.0, 300 mM NaCl, 500 mM imidazole and protein quality were assessed by SDS-PAGE.

### Biolayer interferometry experiments (BLI)

The concentration of fHbp samples was determined using an Octet 384 Red Instrument (Sartorius FortéBio) operating at 30°C and anti-His biosensors. Highly pure fHbp v3.28 was used as the standard curve protein and all protein samples were diluted 1/10 in Octet buffer (1 x phosphate-buffered saline [PBS], 1% w/v bovine serum albumin). Binding kinetics were also measured using BLI at 30°C and anti-Human Fc biosensors. IgG antibodies at a concentration of 10 μg/mL were captured on the biosensor followed by binding of fHbp samples with concentrations ranging from approximately 150 nM–1 nM. For each fHbp-mAb pair, binding traces (average n = 4) were fit using the FortéBio Analysis 11.1 HT software package to determine K_D_, K_on_, and k_off_.

### Differential scanning fluorimetry

Previous studies based on differential scanning calorimetry have demonstrated that fHbp proteins from variant groups 1, 2 and 3 all have two distinct melting points that correspond to the unfolding of the N- and C-terminal domains (Malito et al., 2013; Johnson et al., 2012). To measure both events, while using a minimal amount of protein, the stability of fHbp m002416 was determined using differential scanning fluorimetry. The first melting point (Tm1) was measured using the hydrophobic dye SYPRO orange and corresponds to the unfolding of the N-terminal domain. The second melting point (Tm2) was measured using intrinsic fluorescence and corresponds to the unfolding of the C-terminal domain.

fHbp samples were diluted in PBS to an approximate concentration of 0.5 mg/mL and a total volume of 20 μL. Samples were further diluted with 400 μL of buffer followed by concentration to 50 μL using 0.5 mL, 10 kDa cutoff devices from Millipore. This process was repeated twice to remove imidazole and other buffer components remaining from the purification. Final sample volume was approximately 80 μL. SYPRO Orange hydrophobic dye (ThermoFisher) was diluted from 1000X to 10X in PBS. Chemical DSF samples were prepared in duplicate by mixing 18 mL of protein with 2 mL of dye in a 96-well PCR plate and protecting from light. Samples were analyze using the ViiA 7 System (ThermoFisher) using a ramp speed of 0.02°C/s from 25°C to 99°C. Melting scans were analyzed using Protein Thermal Shift v1.4 and analyzed with default parameters. In addition, fHbp protein samples in PBS were loaded into high-sensitivity capillaries and analyzed using a Prometheus NT.48 system (nanoTemper). The melting scan was performed from 24°C to 110°C with a ramp of 1°C/min. Duplicate measurements were performed for each sample and melting temperature was determined using the manufacturer provided software.

### X-ray crystallography

Purified fHbp 2,416 and JAR5 Fab were combined in a 1:1.1 ratio at room temperature in protein buffer (25 mM Tris pH 8.0, 150 mM NaCl) at a final protein concentration of 11.3 mg/mL. Sparse matrix screening was performed using a Mosquito automated drop setter (SPT Labtech) and drop volumes of 200 nL protein +200 nL precipitant. Crystals were identified in well B8 of the ProPlex screen (Molecular Dimensions); 0.1 M sodium citrate pH 5.0, 15% w/v PEG 4000, 0.1 M magnesium chloride and were harvested after an 8-day incubation at 20°C. 20% glycerol was added as cryoprotectant prior to plunge freezing in liquid nitrogen. Data were collected at the Advance Photon Source, beamline 22-ID equipped with an Eiger 16M detector. The X-ray wavelength was 1.0 Å and 800 images were collected with a frame width of 0.25° at a detector distance of 300 nM. Diffraction patterns were integrated and scaled using XDS (Kabsch, 2010). Data was truncated in XDS to remove frames from the end of the data collection that showed the most radiation damage. Molecular replacement was performed by placing individual models of fHbp v1.1 and JAR5 Fab extracted from PDB 5T5F using Phaser as implemented in Phenix (Adams et al., 2010; McC et al., 2007). The final model was built after iterative rounds of refinement in Phenix and manual corrections in Coot (Emsley et al., 2010). Visualization and analysis of the final model were performed with PyMOL 2.0 (Schrodinger LLC).

## Data Availability

The datasets presented in this article are not readily available due to company data access procedures and safeguards. Requests to access the datasets should be directed to Chelsy Chesterman (chelsy.c.chesterman@gsk.com).

## References

[B1] Abraham M. J. Murtola T. Schulz R. Páll S. Smith J. C. Hess B. (2015). GROMACS: high performance molecular simulations through multi-level parallelism from laptops to supercomputers. SoftwareX 1-2, 19–25. 10.1016/j.softx.2015.06.001

[B2] Abramson J. Adler J. Dunger J. Evans R. Green T. Pritzel A. (2024). Accurate structure prediction of biomolecular interactions with AlphaFold 3. Nature 630 (8016), 493–500. 10.1038/s41586-024-07487-w 38718835 PMC11168924

[B3] Adams P. D. Afonine P. V. Bunkoczi G. Chen V. B. Davis I. W. Echols N. (2010). PHENIX: a comprehensive Python-based system for macromolecular structure solution. Acta Crystallogr. D. Biol. Crystallogr. 66 (Pt 2), 213–221. 10.1107/s0907444909052925 20124702 PMC2815670

[B4] Akbar R. Robert P. A. Pavlovic M. Jeliazkov J. R. Snapkov I. Slabodkin A. (2021). A compact vocabulary of paratope-epitope interactions enables predictability of antibody-antigen binding. Cell Rep. 34 (11), 108856. 10.1016/j.celrep.2021.108856 33730590

[B5] Bachas S. Rakocevic G. Spencer D. Sastry A. V. Haile R. Sutton J. M. (2022). Antibody optimization enabled by artificial intelligence predictions of binding affinity and naturalness. bioRxiv. 10.1101/2022.08.16.504181

[B6] Baden L. R. El Sahly H. M. Essink B. Kotloff K. Frey S. Novak R. (2021). Efficacy and safety of the mRNA-1273 SARS-CoV-2 vaccine. N. Engl. J. Med. 384 (5), 403–416. 10.1056/nejmoa2035389 33378609 PMC7787219

[B7] Bambini S. Muzzi A. Olcen P. Rappuoli R. Pizza M. Comanducci M. (2009). Distribution and genetic variability of three vaccine components in a panel of strains representative of the diversity of serogroup B meningococcus. Vaccine 27 (21), 2794–2803. 10.1016/j.vaccine.2009.02.098 19428890

[B8] Beede E. Baylor E. Hersch F. Iurchenko A. Wilcox L. Ruamviboonsuk P. (2020). “A human-centered evaluation of a deep learning system deployed in clinics for the detection of diabetic retinopathy,” in Proceedings of the 2020 CHI conference on human factors in computing systems, 1–12. 10.1145/3313831.3376718

[B9] Bennett N. R. Watson J. L. Ragotte R. J. Borst A. J. See D. L. Weidle C. (2024). Atomically accurate *de novo* design of antibodies with RFdiffusion. bioRxiv, 2024.03.14.585103. 10.1101/2024.03.14.585103 PMC12727541 41193805

[B10] Berman H. M. Burley S. K. Kleywegt G. J. Markley J. L. Nakamura H. Velankar S. (2016). The archiving and dissemination of biological structure data. Curr. Opin. Struct. Biol. 40, 17–22. 10.1016/j.sbi.2016.06.018 27450113 PMC5161703

[B11] Bianchi F. Veggi D. Santini L. Buricchi F. Bartolini E. Lo Surdo P. (2019). Cocrystal structure of meningococcal factor H binding protein variant 3 reveals a new crossprotective epitope recognized by human mAb 1E6. FASEB J. 33 (11), 12099–12111. 10.1096/fj.201900374r 31442074 PMC6902690

[B12] Brochu E. Cora V. M. de Freitas N. (2010). A tutorial on bayesian optimization of expensive cost functions, with application to active user modeling and hierarchical reinforcement learning. arXiv. 10.48550/arXiv.1012.2599

[B13] Crank M. C. Ruckwardt T. J. Chen M. Morabito K. M. Phung E. Costner P. J. (2019). A proof of concept for structure-based vaccine design targeting RSV in humans. Science 365 (6452), 505–509. 10.1126/science.aav9033 31371616

[B14] D'Amour A. Heller K. Moldovan D. Adlam B. Alipanahi B. Beutel A. (2022). Underspecification presents challenges for credibility in modern machine learning. J. Mach. Learn. Res. 23, 1–61. Available online at: https://www.jmlr.org/papers/volume23/20-1335/20-1335.pdf .

[B15] DeBartolo J. Taipale M. Keating A. E. (2014). Genome-wide prediction and validation of peptides that bind human prosurvival Bcl-2 proteins. PLoS Comput. Biol. 10 (6), e1003693. 10.1371/journal.pcbi.1003693 24967846 PMC4072508

[B16] Desautels T. A. Arrildt K. T. Zemla A. T. Lau E. Y. Zhu F. Ricci D. (2024). Computationally restoring the potency of a clinical antibody against Omicron. Nature 629 (8013), 878–885. 10.1038/s41586-024-07385-1 38720086 PMC11111397

[B17] Desautels T. A. Krause A. Burdick J. (2014). Parallelizing exploration-exploitation tradeoffs in Gaussian process bandit optimization. J. Mach. Learn. Res. 15, 4053–40103. Available online at: https://www.jmlr.org/papers/volume15/desautels14a/desautels14a.pdf .

[B18] Elnaggar A. Heinzinger M. Dallago C. Rehawi G. Wang Y. Jones L. (2022). ProtTrans: toward understanding the language of life through self-supervised learning. IEEE Trans. Pattern Analysis Mach. Intell. 44 (10), 7112–7127. 10.1109/tpami.2021.3095381 34232869

[B19] Emsley P. Lohkamp B. Scott W. G. Cowtan K. (2010). Features and development of Coot. Acta Crystallogr. D. Biol. Crystallogr. 66 (Pt 4), 486–501. 10.1107/s0907444910007493 20383002 PMC2852313

[B20] Giuliani M. M. Santini L. Brunelli B. Biolchi A. Arico B. Di Marcello F. (2005). The region comprising amino acids 100 to 255 of Neisseria meningitidis lipoprotein GNA 1870 elicits bactericidal antibodies. Infect. Immun. 73 (2), 1151–1160. 10.1128/iai.73.2.1151-1160.2005 15664958 PMC546939

[B21] Graves J. Byerly J. Priego E. Makkapati N. Parish S. V. Medellin B. (2020). A review of deep learning methods for antibodies. Antibodies (Basel) 9 (2), 12. 10.3390/antib9020012 32354020 PMC7344881

[B22] Hie B. L. Shanker V. R. Xu D. Bruun T. U. J. Weidenbacher P. A. Tang S. (2024). Efficient evolution of human antibodies from general protein language models. Nat. Biotechnol. 42 (2), 275–283. 10.1038/s41587-023-01763-2 37095349 PMC10869273

[B23] Hsieh C. L. Goldsmith J. A. Schaub J. M. DiVenere A. M. Kuo H. C. Javanmardi K. (2020). Structure-based design of prefusion-stabilized SARS-CoV-2 spikes. Science 369 (6510), 1501–1505. 10.1126/science.abd0826 32703906 PMC7402631

[B24] Johnson S. Tan L. van der Veen S. Caesar J. Goicoechea De Jorge E. Harding R. J. (2012). Design and evaluation of meningococcal vaccines through structure-based modification of host and pathogen molecules. PLoS Pathog. 8 (10), e1002981. 10.1371/journal.ppat.1002981 23133374 PMC3486911

[B25] Jokinen E. Heinonen M. Lahdesmaki H. (2018). mGPfusion: predicting protein stability changes with Gaussian process kernel learning and data fusion. Bioinformatics 34 (13), i274–i283. 10.1093/bioinformatics/bty238 29949987 PMC6022679

[B26] Jolley K. A. Maiden M. C. (2010). BIGSdb: scalable analysis of bacterial genome variation at the population level. BMC Bioinforma. 11, 595. 10.1186/1471-2105-11-595 PMC3004885 21143983

[B27] Kabsch W. (2010). Xds. Acta Crystallogr. D. Biol. Crystallogr. 66 (Pt 2), 125–132. 10.1107/s0907444909047337 20124692 PMC2815665

[B28] Lalmuanawma S. Hussain J. Chhakchhuak L. (2020). Applications of machine learning and artificial intelligence for Covid-19 (SARS-CoV-2) pandemic: a review. Chaos Solit. Fractals 139, 110059. 10.1016/j.chaos.2020.110059 PMC7315944 32834612

[B29] Liu Y. (2004). Active learning with support vector machine applied to gene expression data for cancer classification. J. Chem. Inf. Comput. Sci. 44 (6), 1936–1941. 10.1021/ci049810a 15554662

[B30] Lopez-Sagaseta J. Beernink P. T. Bianchi F. Santini L. Frigimelica E. Lucas A. H. (2018). Crystal structure reveals vaccine elicited bactericidal human antibody targeting a conserved epitope on meningococcal fHbp. Nat. Commun. 9 (1), 528. 10.1038/s41467-018-02827-7 29410413 PMC5802752

[B31] Malito E. Faleri A. Lo Surdo P. Veggi D. Maruggi G. Grassi E. (2013). Defining a protective epitope on factor H binding protein, a key meningococcal virulence factor and vaccine antigen. Proc. Natl. Acad. Sci. U. S. A. 110 (9), 3304–3309. 10.1073/pnas.1222845110 23396847 PMC3587270

[B32] Malito E. Lo Surdo P. Veggi D. Santini L. Stefek H. Brunelli B. (2016). Neisseria meningitidis factor H-binding protein bound to monoclonal antibody JAR5: implications for antibody synergy. Biochem. J. 473 (24), 4699–4713. 10.1042/bcj20160806 27784765 PMC6398935

[B33] Masignani V. Comanducci M. Giuliani M. M. Bambini S. Adu-Bobie J. Arico B. (2003). Vaccination against Neisseria meningitidis using three variants of the lipoprotein GNA1870. J. Exp. Med. 197 (6), 789–799. 10.1084/jem.20021911 12642606 PMC2193853

[B34] McCoy A. J. Grosse-Kunstleve R. W. Adams P. D. Winn M. D. Storoni L. C. Read R. J. (2007). Phaser crystallographic software. J. Appl. Crystallogr. 40 (Pt 4), 658–674. 10.1107/s0021889807021206 19461840 PMC2483472

[B35] McCulloch R. Cobbold C. A. Figueiredo L. Jackson A. Morrison L. J. Mugnier M. R. (2017). Emerging challenges in understanding trypanosome antigenic variation. Emerg. Top. Life Sci. 1 (6), 585–592. 10.1042/etls20170104 30271884 PMC6162063

[B36] Mellor J. Grigoras I. Carbonell P. Faulon J. L. (2016). Semisupervised Gaussian process for automated enzyme search. ACS Synth. Biol. 5 (6), 518–528. 10.1021/acssynbio.5b00294 27007080

[B37] Murphy E. Andrew L. Lee K. L. Dilts D. A. Nunez L. Fink P. S. (2009). Sequence diversity of the factor H binding protein vaccine candidate in epidemiologically relevant strains of serogroup B Neisseria meningitidis. J. Infect. Dis. 200 (3), 379–389. 10.1086/600141 19534597

[B38] Neu K. E. Henry Dunand C. J. Wilson P. C. (2016). Heads, stalks and everything else: how can antibodies eradicate influenza as a human disease? Curr. Opin. Immunol. 42, 48–55. 10.1016/j.coi.2016.05.012 27268395 PMC5086271

[B39] Ng'uni T. Chasara C. Ndhlovu Z. M. (2020). Major scientific hurdles in HIV vaccine development: historical perspective and future directions. Front. Immunol. 11, 590780. 10.3389/fimmu.2020.590780 33193428 PMC7655734

[B40] Ong E. Wong M. U. Huffman A. He Y. (2020). COVID-19 coronavirus vaccine design using reverse vaccinology and machine learning. Front. Immunol. 11, 1581. 10.3389/fimmu.2020.01581 32719684 PMC7350702

[B41] Pace D. Pollard A. J. (2012). Meningococcal disease: clinical presentation and sequelae. Vaccine 30 (Suppl. 2), B3–B9. 10.1016/j.vaccine.2011.12.062 22607896

[B42] Pedregosa F. Varoquaux G. Gramfort A. Michel V. Thirion B. Grisel O. (2011). Scikit-learn: machine learning in Python. J. Mach. Learn. Res. 12, 2825–2830. Available online at: https://www.jmlr.org/papers/volume12/pedregosa11a/pedregosa11a.pdf?source=post_page .

[B43] Pires D. E. Ascher D. B. Blundell T. L. (2014). mCSM: predicting the effects of mutations in proteins using graph-based signatures. Bioinformatics 30 (3), 335–342. 10.1093/bioinformatics/btt691 24281696 PMC3904523

[B44] Pizza M. Scarlato V. Masignani V. Giuliani M. M. Arico B. Comanducci M. (2000). Identification of vaccine candidates against serogroup B meningococcus by whole-genome sequencing. Science 287 (5459), 1816–1820. 10.1126/science.287.5459.1816 10710308

[B45] Polack F. P. Thomas S. J. Kitchin N. Absalon J. Gurtman A. Lockhart S. (2020). Safety and efficacy of the BNT162b2 mRNA covid-19 vaccine. N. Engl. J. Med. 383 (27), 2603–2615. 10.1056/nejmoa2034577 33301246 PMC7745181

[B46] Prihoda D. Maamary J. Waight A. Juan V. Fayadat-Dilman L. Svozil D. (2022). BioPhi: a platform for antibody design, humanization, and humanness evaluation based on natural antibody repertoires and deep learning. MAbs 14 (1), 2020203. 10.1080/19420862.2021.2020203 35133949 PMC8837241

[B47] Rappuoli R. (2001). Reverse vaccinology, a genome-based approach to vaccine development. Vaccine 19 (17-19), 2688–2691. 10.1016/s0264-410x(00)00554-5 11257410

[B48] Rappuoli R. (2007). Bridging the knowledge gaps in vaccine design. Nat. Biotechnol. 25 (12), 1361–1366. 10.1038/nbt1207-1361 18066025

[B49] Rappuoli R. Bottomley M. J. D'Oro U. Finco O. De Gregorio E. (2016). Reverse vaccinology 2.0: human immunology instructs vaccine antigen design. J. Exp. Med. 213 (4), 469–481. 10.1084/jem.20151960 27022144 PMC4821650

[B50] Rasmussen C. E. (2004). “Gaussian processes in machine learning,” Advanced lectures on machine learning ML 2003 lecture notes in computer science. Editors Bousquet O. von Luxburg U. Rätsch G. (Berlin, Heidelberg: Springer), 3176, 63–71. 10.1007/978-3-540-28650-9_4

[B51] Ren Y. Chen X. Feng M. Wang Q. Zhou P. (2011). Gaussian process: a promising approach for the modeling and prediction of Peptide binding affinity to MHC proteins. Protein Pept. Lett. 18 (7), 670–678. 10.2174/092986611795445978 21413918

[B52] Riahi S. Lee J. H. Wei S. Cost R. Masiero A. Prades C. (2021). Application of an integrated computational antibody engineering platform to design SARS-CoV-2 neutralizers. Antib. Ther. 4 (2), 109–122. 10.1093/abt/tbab011 34396040 PMC8344454

[B53] Romero P. A. Krause A. Arnold F. H. (2013). Navigating the protein fitness landscape with Gaussian processes. Proc. Natl. Acad. Sci. U. S. A. 110 (3), E193–E201. 10.1073/pnas.1215251110 23277561 PMC3549130

[B54] Rosenstein N. E. Perkins B. A. Stephens D. S. Popovic T. Hughes J. M. (2001). Meningococcal disease. N. Engl. J. Med. 344 (18), 1378–1388. 10.1056/nejm200105033441807 11333996

[B55] Saito Y. Oikawa M. Nakazawa H. Niide T. Kameda T. Tsuda K. (2018). Machine-learning-Guided mutagenesis for directed evolution of fluorescent proteins. ACS Synth. Biol. 7 (9), 2014–2022. 10.1021/acssynbio.8b00155 30103599

[B56] Schneider P. Walters W. P. Plowright A. T. Sieroka N. Listgarten J. Goodnow R. A. Jr (2020). Rethinking drug design in the artificial intelligence era. Nat. Rev. Drug Discov. 19 (5), 353–364. 10.1038/s41573-019-0050-3 31801986

[B57] Schymkowitz J. Borg J. Stricher F. Nys R. Rousseau F. Serrano L. (2005). The FoldX web server: an online force field. Nucleic Acids Res. 33 (Web Server issue), W382–W388. 10.1093/nar/gki387 15980494 PMC1160148

[B58] Seib K. L. Scarselli M. Comanducci M. Toneatto D. Masignani V. (2015). Neisseria meningitidis factor H-binding protein fHbp: a key virulence factor and vaccine antigen. Expert Rev. Vaccines 14 (6), 841–859. 10.1586/14760584.2015.1016915 25704037

[B59] Serruto D. Bottomley M. J. Ram S. Giuliani M. M. Rappuoli R. (2012). The new multicomponent vaccine against meningococcal serogroup B, 4CMenB: immunological, functional and structural characterization of the antigens. Vaccine 30 (Suppl. 2), B87–B97. 10.1016/j.vaccine.2012.01.033 22607904 PMC3360877

[B60] Shahriari B. Swersky K. Wang Z. Adams R. P. de Freitas N. (2016). Taking the human out of the loop: a review of bayesian optimization. Proc. IEEE 104 (1), 148–175. 10.1109/jproc.2015.2494218

[B61] Shanehsazzadeh A. McPartlon M. Kasun G. Steiger A. K. Sutton J. M. Yassine E. (2024). Unlocking *de novo* antibody design with generative artificial intelligence. bioRxiv. 10.1101/2023.01.08.523187

[B62] Sirin S. Apgar J. R. Bennett E. M. Keating A. E. (2016). AB-Bind: antibody binding mutational database for computational affinity predictions. Protein Sci. 25 (2), 393–409. 10.1002/pro.2829 26473627 PMC4815335

[B63] Stanton S. Maddox W. Gruver N. Maffettone P. Delaney E. Greenside P. (2022). Accelerating bayesian optimization for biological sequence design with denoising autoencoders. Proc. 39th Int. Conf. Mach. Learn. 162, 20459–20478. Available online at: https://proceedings.mlr.press/v162/stanton22a/stanton22a.pdf .

[B64] Tokuriki N. Tawfik D. S. (2009). Stability effects of mutations and protein evolvability. Curr. Opin. Struct. Biol. 19 (5), 596–604. 10.1016/j.sbi.2009.08.003 19765975

[B65] Veggi D. Bianchi F. Santini L. Lo Surdo P. Chesterman C. C. Pansegrau W. (2020). 4CMenB vaccine induces elite cross-protective human antibodies that compete with human factor H for binding to meningococcal fHbp. PLoS Pathog. 16 (10), e1008882. 10.1371/journal.ppat.1008882 33007046 PMC7556464

[B66] Verkuil R. Kabeli O. Du Y. Wicky B. I. M. Milles L. F. Dauparas J. (2022). Language models generalize beyond natural proteins. bioRxiv. 10.1101/2022.12.21.521521

[B67] Yang K. K. Wu Z. Arnold F. H. (2019). Machine-learning-guided directed evolution for protein engineering. Nat. Methods 16 (8), 687–694. 10.1038/s41592-019-0496-6 31308553

[B68] Zemla A. Zhou C. E. Slezak T. Kuczmarski T. Rama D. Torres C. (2005). AS2TS system for protein structure modeling and analysis. Nucleic Acids Res. 33 (Web Server issue), W111–W115. 10.1093/nar/gki457 15980437 PMC1160218

[B69] Zemla A. T. Lang D. M. Kostova T. Andino R. Ecale Zhou C. L. (2011). StralSV: assessment of sequence variability within similar 3D structures and application to polio RNA-dependent RNA polymerase. BMC Bioinforma. 12, 226. 10.1186/1471-2105-12-226 PMC3121648 21635786

[B70] Zlotnick G. W. Jones T. R. Liberator P. Hao L. Harris S. McNeil L. K. (2015). The discovery and development of a novel vaccine to protect against Neisseria meningitidis Serogroup B Disease. Hum. Vaccin Immunother. 11 (1), 5–13. 10.4161/hv.34293 25483509 PMC4514153

