# Supplementary material for: Design of cross-reactive antigens with machine learning and high-throughput experimental evaluation
Source: Front Bioinform. 2025 Jul 16;5:1580967. doi: 10.3389/fbinf.2025.1580967 (PMC12319226; doi:10.3389/fbinf.2025.1580967)
Supplement: Supplementary file 3 [file Supplementaryfile1.docx]

Document S1

#########################################

# Homology modeling of fab-fHbp complexes was done using 5 known PDB complexes:

# 2ypv 5o14 5t5f xxx1 6h2y

#

# 1fab - Fab from P2ypv (12C1)

# 2fab - Fab from P5o14 (1A12)

# 3fab - Fab from P5t5f (JAR5)

# 4fab - Fab from Pxxx1 (4B3)

# 5fab - Fab from P6h2y (1E6)

#

#########################################

# Set of 10 "master" sequences with original headers:

#

# >fH_V1_P2ypv_WT Wild_type (m0001)

# VAADIGAGLADALTAPLDHKDKGLQSLTLDQSVRKNEKLKLAAQGAEKTYGNGDSLNTGKLKNDKVSRFDFIRQIEVDGQLITLESGEFQVYKQSHSALTAFQTEQIQDSEHSGKMVAKRQFRIGDIAGEHTSFDKLPEGGRATYRGTAFGSDDAGGKLTYTIDFAAKQGNGKIEHLKSPELNVDLAAADIKPDGKRHAVISGSVLYNQAEKGSYSLGIFGGKAQEVAGSAEVKTVNGIRHIGLAAKQ

#

# >fH_V3_P6h2y_WT Wild_type (m0002)

# VAADIGTGLADALTAPLDHKDKGLKSLTLEDSIPQNGTLTLSAQGAEKTFKAGDKDNSLNTGKLKNDKISRFDFVQKIEVDGQTITLASGEFQIYKQNHSAVVALQIEKINNPDKTDSLINQRSFLVSGLGGEHTAFNQLPGGKAEYHGKAFSSDDPNGRLHYSIDFTKKQGYGRIEHLKTLEQNVELAAAELKADEKSHAVILGDTRYGSEEKGTYHLALFGDRAQEIAGSATVKIGEKVHEIGIAGKQ

#

# >fH_V1_V3_ch01 chimera_01 (m0003)

# VAADIGAGLADALTAPLDHKDKGLQSLTLDQSVRKNETLTLSAQGAEKTYGNGDSLNTGKLKNDKVSRFDFVRKIEVDGQLITLESGEFQVYKQSHSALTAFQTEKIQDSEHSGKLVNKRQFRIGDIAGEHTSFDKLPEGGRATYRGTAFGSDDPNGKLTYTIDFAAKQGNGKIEHLKSPELNVDLAAADIKPDGKRHAVISGSVLYNQAEKGSYSLGIFGGKAQEVAGSAEVKTVNGIRHIGLAAKQ

#

# >fH_V3_V1_ch02 chimera_02 (m0004)

# VAADIGTGLADALTAPLDHKDKGLKSLTLEDSISQNGTLTLSAQGAEKTFKVGDKDNSLNTGKLKNDKISRFDFVQKIEVDGQTITLASGEFQIYKQDHSAVVALQIEQIQDSEHSGKMVNQRSFLVSGLGGEHTAFNQLPGGKAEYHGKAFSSDDPNGRLHYSIDFTKKQGYGRIEHLKTPEQNVELAAAELKADEKSHAVILGDTRYGSEEKGTYHLALFGDRAQEIAGSATVKIGEKVHEIGIAGKQ

#

# >fH_V3_28_ch51 chimera_51 (m0005)

# VAADIGTGLADALTAPLDHKDKGLKSLTLEDSIRQNGTLTLSAQGAEKTFKAGDKDNSLNTGKLKNDKISRFDFVQKIEVDGQTITLASGEFQIYKQNHSAVVALQIEQIQDSEHSGKMVNKRQFRISGLGGEHTAFNQLPGGKAEYHGKAFSSDDPNGRLHYSIDFTKKQGYGRIEHLKTLEQNVELAAAELKADEKSHAVILGDTRYNSAEKGSYHLALFGDRAQEIAGSAEVKTVNGIHHIGIAGKQ

#

# >fH_V3_28_ch52 chimera_52 (m0006)

# VAADIGTGLADALTAPLDHKDKGLKSLTLEDSIRQNGTLTLSAQGAEKTFKAGDKDNSLNTGKLKNDKISRFDFVQKIEVDGQTITLASGEFQIYKQNHSAVVALQIEKIQDSEHSGKLVNKRQFRISGLGGEHTAFNQLPGGKAEYHGKAFSSDDPNGRLHYSIDFTKKQGYGRIEHLKTLEQNVELAAAELKADEKSHAVILGDTRYGSAEKGSYHLALFGDRAQEIAGSAEVKTGEGIHHIGIAGKQ

#

# >fH_V3_28_ch53 chimera_53 (m0007)

# VAADIGTGLADALTAPLDHKDKGLKSLTLEDSIRQNGTLTLSAQGAEKTFKAGDKDNSLNTGKLKNDKISRFDFVQKIEVDGQTITLASGEFQIYKQNHSAVVALQIEEIQDSEHSGKAVNKRQFRISGLGGEHTAFNQLPGGKAEYHGKAFSSDDPNGRLHYSIDFTKKQGYGRIEHLKTLEQNVELAAAELKADEKSHAVILGDTRYDSAEKGSYHLALFGDRAQEIAGSAEVKTADGIHHIGIAGKQ

#

# >fH_V3_28_ch71 chimera_71 (m0008)

# VAADIGTGLADALTAPLDHKDKGLKSLTLEQSIRQNETLTLSAQGAEKTFKAGDKDNSLNTGKLKNDKISRFDFVRKIEVDGQLITLESGEFQIYKQNHSAVVAFQIEQIQDSEHSGKMVAKRQFRISGLGGEHTAFNQLPGGKAEYHGKAFSSDDPNGRLHYSIDFTKKQGYGRIEHLKTLEQNVELAAAELKADEKSHAVISGSVRYNQAEKGSYSLALFGDRAQEIAGSAEVKTVNGIRHIGIAGKQ

#

# >fH_V3_28_ch72 chimera_72 (m0009)

# VAADIGTGLADALTAPLDHKDKGLKSLTLEQSIRQNETLTLSAQGAEKTFKAGDKDNSLNTGKLKNDKISRFDFVRKIEVDGQLITLASGEFQIYKQNHSAVVAFQIEKIQDSEHSGKLVNQRSFRISGLGGEHTAFNQLPGGKAEYHGKAFSSDDPNGRLHYSIDFTKKQGYGRIEHLKTLEQNVELAAAELKADEKSHAVILGDVRYGSEEKGSYSLALFGDRAQEIAGSAEVKTGEGIRHIGIAGKQ

#

# >fH_V3_28_ch73 chimera_73 (m0010)

# VAADIGTGLADALTAPLDHKDKGLKSLTLEQSIRQNETLTLSAQGAEKTFKAGDKDNSLNTGKLKNDKISRFDFVRKIEVDGQLITLSSGEFQIYKQNHSAVVAFQIEEIQDSEHSGKAVDTRGFRISGLGGEHTAFNQLPGGKAEYHGKAFSSDDPNGRLHYSIDFTKKQGYGRIEHLKTLEQNVELAAAELKADEKSHAVITGKVRYDGDEKGSYSLALFGDRAQEIAGSAEVKTADGIRHIGIAGKQ

#

#########################################

# All mutants were constructed by mutating closest "master" sequences

# Evaluation of all constructed mutants (131 : 10 "master" antigens + 121 additional mutants) was performed by calculating DDGs

# with 2 "master" sequences (m0001) and (m0002) used as initial (prior mutation) references:

#########################################

#

# Set of 10 "master" sequences with headers in the same format as all other constructed mutants:

#

>Mutant_number:m0001 Master.m0001: fH_V1_P2ypv_WT Mutations: -

VAADIGAGLADALTAPLDHKDKGLQSLTLDQSVRKNEKLKLAAQGAEKTYGNGDSLNTGKLKNDKVSRFDFIRQIEVDGQLITLESGEFQVYKQSHSALTAFQTEQIQDSEHSGKMVAKRQFRIGDIAGEHTSFDKLPEGGRATYRGTAFGSDDAGGKLTYTIDFAAKQGNGKIEHLKSPELNVDLAAADIKPDGKRHAVISGSVLYNQAEKGSYSLGIFGGKAQEVAGSAEVKTVNGIRHIGLAAKQ

>Mutant_number:m0002 Master.m0002: fH_V3_P6h2y_WT Mutations: -

VAADIGTGLADALTAPLDHKDKGLKSLTLEDSIPQNGTLTLSAQGAEKTFKAGDKDNSLNTGKLKNDKISRFDFVQKIEVDGQTITLASGEFQIYKQNHSAVVALQIEKINNPDKTDSLINQRSFLVSGLGGEHTAFNQLPGGKAEYHGKAFSSDDPNGRLHYSIDFTKKQGYGRIEHLKTLEQNVELAAAELKADEKSHAVILGDTRYGSEEKGTYHLALFGDRAQEIAGSATVKIGEKVHEIGIAGKQ

>Mutant_number:m0003 Master.m0001: fH_V1_P2ypv_WT Mutations: KA38T,KA40T,AA42S,IA72V,QA74K,QA106K,MA116L,AA118N,AA155P,GA156N

VAADIGAGLADALTAPLDHKDKGLQSLTLDQSVRKNETLTLSAQGAEKTYGNGDSLNTGKLKNDKVSRFDFVRKIEVDGQLITLESGEFQVYKQSHSALTAFQTEKIQDSEHSGKLVNKRQFRIGDIAGEHTSFDKLPEGGRATYRGTAFGSDDPNGKLTYTIDFAAKQGNGKIEHLKSPELNVDLAAADIKPDGKRHAVISGSVLYNQAEKGSYSLGIFGGKAQEVAGSAEVKTVNGIRHIGLAAKQ

>Mutant_number:m0004 Master.m0002: fH_V3_P6h2y_WT Mutations: PA34S,AA52V,NA98D,KA109Q,NA111Q,NA112D,PA113S,DA114E,KA115H,TA116S,DA117G,SA118K,LA119M,IA120V,LA182P

VAADIGTGLADALTAPLDHKDKGLKSLTLEDSISQNGTLTLSAQGAEKTFKVGDKDNSLNTGKLKNDKISRFDFVQKIEVDGQTITLASGEFQIYKQDHSAVVALQIEQIQDSEHSGKMVNQRSFLVSGLGGEHTAFNQLPGGKAEYHGKAFSSDDPNGRLHYSIDFTKKQGYGRIEHLKTPEQNVELAAAELKADEKSHAVILGDTRYGSEEKGTYHLALFGDRAQEIAGSATVKIGEKVHEIGIAGKQ

>Mutant_number:m0005 Master.m0002: fH_V3_P6h2y_WT Mutations: PA34R,KA109Q,NA111Q,NA112D,PA113S,DA114E,KA115H,TA116S,DA117G,SA118K,LA119M,IA120V,QA122K,SA124Q,LA126R,VA127I,GA210N,EA212A,TA216S,TA234E,IA237T,GA238V,EA239N,KA240G,VA241I,EA243H

VAADIGTGLADALTAPLDHKDKGLKSLTLEDSIRQNGTLTLSAQGAEKTFKAGDKDNSLNTGKLKNDKISRFDFVQKIEVDGQTITLASGEFQIYKQNHSAVVALQIEQIQDSEHSGKMVNKRQFRISGLGGEHTAFNQLPGGKAEYHGKAFSSDDPNGRLHYSIDFTKKQGYGRIEHLKTLEQNVELAAAELKADEKSHAVILGDTRYNSAEKGSYHLALFGDRAQEIAGSAEVKTVNGIHHIGIAGKQ

>Mutant_number:m0006 Master.m0002: fH_V3_P6h2y_WT Mutations: PA34R,NA111Q,NA112D,PA113S,DA114E,KA115H,TA116S,DA117G,SA118K,IA120V,QA122K,SA124Q,LA126R,VA127I,EA212A,TA216S,TA234E,IA237T,KA240G,VA241I,EA243H

VAADIGTGLADALTAPLDHKDKGLKSLTLEDSIRQNGTLTLSAQGAEKTFKAGDKDNSLNTGKLKNDKISRFDFVQKIEVDGQTITLASGEFQIYKQNHSAVVALQIEKIQDSEHSGKLVNKRQFRISGLGGEHTAFNQLPGGKAEYHGKAFSSDDPNGRLHYSIDFTKKQGYGRIEHLKTLEQNVELAAAELKADEKSHAVILGDTRYGSAEKGSYHLALFGDRAQEIAGSAEVKTGEGIHHIGIAGKQ

>Mutant_number:m0007 Master.m0002: fH_V3_P6h2y_WT Mutations: PA34R,KA109E,NA111Q,NA112D,PA113S,DA114E,KA115H,TA116S,DA117G,SA118K,LA119A,IA120V,QA122K,SA124Q,LA126R,VA127I,GA210D,EA212A,TA216S,TA234E,IA237T,GA238A,EA239D,KA240G,VA241I,EA243H

VAADIGTGLADALTAPLDHKDKGLKSLTLEDSIRQNGTLTLSAQGAEKTFKAGDKDNSLNTGKLKNDKISRFDFVQKIEVDGQTITLASGEFQIYKQNHSAVVALQIEEIQDSEHSGKAVNKRQFRISGLGGEHTAFNQLPGGKAEYHGKAFSSDDPNGRLHYSIDFTKKQGYGRIEHLKTLEQNVELAAAELKADEKSHAVILGDTRYDSAEKGSYHLALFGDRAQEIAGSAEVKTADGIHHIGIAGKQ

>Mutant_number:m0008 Master.m0002: fH_V3_P6h2y_WT Mutations: DA31Q,PA34R,GA37E,QA76R,TA84L,AA88E,LA105F,KA109Q,NA111Q,NA112D,PA113S,DA114E,KA115H,TA116S,DA117G,SA118K,LA119M,IA120V,NA121A,QA122K,SA124Q,LA126R,VA127I,LA204S,DA206S,TA207V,GA210N,SA211Q,EA212A,TA216S,HA218S,TA234E,IA237T,GA238V,EA239N,KA240G,VA241I,HA242R,EA243H

VAADIGTGLADALTAPLDHKDKGLKSLTLEQSIRQNETLTLSAQGAEKTFKAGDKDNSLNTGKLKNDKISRFDFVRKIEVDGQLITLESGEFQIYKQNHSAVVAFQIEQIQDSEHSGKMVAKRQFRISGLGGEHTAFNQLPGGKAEYHGKAFSSDDPNGRLHYSIDFTKKQGYGRIEHLKTLEQNVELAAAELKADEKSHAVISGSVRYNQAEKGSYSLALFGDRAQEIAGSAEVKTVNGIRHIGIAGKQ

>Mutant_number:m0009 Master.m0002: fH_V3_P6h2y_WT Mutations: DA31Q,PA34R,GA37E,QA76R,TA84L,LA105F,NA111Q,NA112D,PA113S,DA114E,KA115H,TA116S,DA117G,SA118K,IA120V,LA126R,VA127I,TA207V,TA216S,HA218S,TA234E,IA237T,KA240G,VA241I,HA242R,EA243H

VAADIGTGLADALTAPLDHKDKGLKSLTLEQSIRQNETLTLSAQGAEKTFKAGDKDNSLNTGKLKNDKISRFDFVRKIEVDGQLITLASGEFQIYKQNHSAVVAFQIEKIQDSEHSGKLVNQRSFRISGLGGEHTAFNQLPGGKAEYHGKAFSSDDPNGRLHYSIDFTKKQGYGRIEHLKTLEQNVELAAAELKADEKSHAVILGDVRYGSEEKGSYSLALFGDRAQEIAGSAEVKTGEGIRHIGIAGKQ

>Mutant_number:m0010 Master.m0002: fH_V3_P6h2y_WT Mutations: DA31Q,PA34R,GA37E,QA76R,TA84L,AA88S,LA105F,KA109E,NA111Q,NA112D,PA113S,DA114E,KA115H,TA116S,DA117G,SA118K,LA119A,IA120V,NA121D,QA122T,SA124G,LA126R,VA127I,LA204T,DA206K,TA207V,GA210D,SA211G,EA212D,TA216S,HA218S,TA234E,IA237T,GA238A,EA239D,KA240G,VA241I,HA242R,EA243H

VAADIGTGLADALTAPLDHKDKGLKSLTLEQSIRQNETLTLSAQGAEKTFKAGDKDNSLNTGKLKNDKISRFDFVRKIEVDGQLITLSSGEFQIYKQNHSAVVAFQIEEIQDSEHSGKAVDTRGFRISGLGGEHTAFNQLPGGKAEYHGKAFSSDDPNGRLHYSIDFTKKQGYGRIEHLKTLEQNVELAAAELKADEKSHAVITGKVRYDGDEKGSYSLALFGDRAQEIAGSAEVKTADGIRHIGIAGKQ

#########################################

#

# Set of additional 121 constructed mutants (by mutating WT sequences i.e. using 2 masters (m0001, m0002) as initial sequences for the new constructs):

#

>Mutant_number:m0011 Master.m0001: fH_V1_P2ypv_WT Mutations: KA38T,KA40T,AA42S,EA76R,VA77S

VAADIGAGLADALTAPLDHKDKGLQSLTLDQSVRKNETLTLSAQGAEKTYGNGDSLNTGKLKNDKVSRFDFIRQIRSDGQLITLESGEFQVYKQSHSALTAFQTEQIQDSEHSGKMVAKRQFRIGDIAGEHTSFDKLPEGGRATYRGTAFGSDDAGGKLTYTIDFAAKQGNGKIEHLKSPELNVDLAAADIKPDGKRHAVISGSVLYNQAEKGSYSLGIFGGKAQEVAGSAEVKTVNGIRHIGLAAKQ

>Mutant_number:m0012 Master.m0001: fH_V1_P2ypv_WT Mutations: IA72V,QA74K

VAADIGAGLADALTAPLDHKDKGLQSLTLDQSVRKNEKLKLAAQGAEKTYGNGDSLNTGKLKNDKVSRFDFVRKIEVDGQLITLESGEFQVYKQSHSALTAFQTEQIQDSEHSGKMVAKRQFRIGDIAGEHTSFDKLPEGGRATYRGTAFGSDDAGGKLTYTIDFAAKQGNGKIEHLKSPELNVDLAAADIKPDGKRHAVISGSVLYNQAEKGSYSLGIFGGKAQEVAGSAEVKTVNGIRHIGLAAKQ

>Mutant_number:m0013 Master.m0001: fH_V1_P2ypv_WT Mutations: EA76R,VA77S,MA116L,AA118N

VAADIGAGLADALTAPLDHKDKGLQSLTLDQSVRKNEKLKLAAQGAEKTYGNGDSLNTGKLKNDKVSRFDFIRQIRSDGQLITLESGEFQVYKQSHSALTAFQTEQIQDSEHSGKLVNKRQFRIGDIAGEHTSFDKLPEGGRATYRGTAFGSDDAGGKLTYTIDFAAKQGNGKIEHLKSPELNVDLAAADIKPDGKRHAVISGSVLYNQAEKGSYSLGIFGGKAQEVAGSAEVKTVNGIRHIGLAAKQ

>Mutant_number:m0014 Master.m0001: fH_V1_P2ypv_WT Mutations: AA155P,GA156N

VAADIGAGLADALTAPLDHKDKGLQSLTLDQSVRKNEKLKLAAQGAEKTYGNGDSLNTGKLKNDKVSRFDFIRQIEVDGQLITLESGEFQVYKQSHSALTAFQTEQIQDSEHSGKMVAKRQFRIGDIAGEHTSFDKLPEGGRATYRGTAFGSDDPNGKLTYTIDFAAKQGNGKIEHLKSPELNVDLAAADIKPDGKRHAVISGSVLYNQAEKGSYSLGIFGGKAQEVAGSAEVKTVNGIRHIGLAAKQ

>Mutant_number:m0015 Master.m0001: fH_V1_P2ypv_WT Mutations: IA72V,QA74K,QA106K,MA116L,AA118N,AA155P,GA156N

VAADIGAGLADALTAPLDHKDKGLQSLTLDQSVRKNEKLKLAAQGAEKTYGNGDSLNTGKLKNDKVSRFDFVRKIEVDGQLITLESGEFQVYKQSHSALTAFQTEKIQDSEHSGKLVNKRQFRIGDIAGEHTSFDKLPEGGRATYRGTAFGSDDPNGKLTYTIDFAAKQGNGKIEHLKSPELNVDLAAADIKPDGKRHAVISGSVLYNQAEKGSYSLGIFGGKAQEVAGSAEVKTVNGIRHIGLAAKQ

>Mutant_number:m0016 Master.m0001: fH_V1_P2ypv_WT Mutations: KA38T,KA40T,AA42S,EA76R,VA77S,QA106K,MA116L,AA118N,AA155P,GA156N

VAADIGAGLADALTAPLDHKDKGLQSLTLDQSVRKNETLTLSAQGAEKTYGNGDSLNTGKLKNDKVSRFDFIRQIRSDGQLITLESGEFQVYKQSHSALTAFQTEKIQDSEHSGKLVNKRQFRIGDIAGEHTSFDKLPEGGRATYRGTAFGSDDPNGKLTYTIDFAAKQGNGKIEHLKSPELNVDLAAADIKPDGKRHAVISGSVLYNQAEKGSYSLGIFGGKAQEVAGSAEVKTVNGIRHIGLAAKQ

>Mutant_number:m0017 Master.m0001: fH_V1_P2ypv_WT Mutations: KA38T,KA40T,AA42S,IA72V,QA74K,MA116L,AA118N,AA155P,GA156N

VAADIGAGLADALTAPLDHKDKGLQSLTLDQSVRKNETLTLSAQGAEKTYGNGDSLNTGKLKNDKVSRFDFVRKIEVDGQLITLESGEFQVYKQSHSALTAFQTEQIQDSEHSGKLVNKRQFRIGDIAGEHTSFDKLPEGGRATYRGTAFGSDDPNGKLTYTIDFAAKQGNGKIEHLKSPELNVDLAAADIKPDGKRHAVISGSVLYNQAEKGSYSLGIFGGKAQEVAGSAEVKTVNGIRHIGLAAKQ

>Mutant_number:m0018 Master.m0001: fH_V1_P2ypv_WT Mutations: KA38T,KA40T,AA42S,IA72V,QA74K,QA106K,AA155P,GA156N

VAADIGAGLADALTAPLDHKDKGLQSLTLDQSVRKNETLTLSAQGAEKTYGNGDSLNTGKLKNDKVSRFDFVRKIEVDGQLITLESGEFQVYKQSHSALTAFQTEKIQDSEHSGKMVAKRQFRIGDIAGEHTSFDKLPEGGRATYRGTAFGSDDPNGKLTYTIDFAAKQGNGKIEHLKSPELNVDLAAADIKPDGKRHAVISGSVLYNQAEKGSYSLGIFGGKAQEVAGSAEVKTVNGIRHIGLAAKQ

>Mutant_number:m0019 Master.m0001: fH_V1_P2ypv_WT Mutations: KA38T,KA40T,AA42S,IA72V,QA74K,QA106K,MA116L,AA118N

VAADIGAGLADALTAPLDHKDKGLQSLTLDQSVRKNETLTLSAQGAEKTYGNGDSLNTGKLKNDKVSRFDFVRKIEVDGQLITLESGEFQVYKQSHSALTAFQTEKIQDSEHSGKLVNKRQFRIGDIAGEHTSFDKLPEGGRATYRGTAFGSDDAGGKLTYTIDFAAKQGNGKIEHLKSPELNVDLAAADIKPDGKRHAVISGSVLYNQAEKGSYSLGIFGGKAQEVAGSAEVKTVNGIRHIGLAAKQ

>Mutant_number:m0020 Master.m0002: fH_V3_P6h2y_WT Mutations: PA34R,GA37E,AA52V,NA98D,LA182P

VAADIGTGLADALTAPLDHKDKGLKSLTLEDSIRQNETLTLSAQGAEKTFKVGDKDNSLNTGKLKNDKISRFDFVQKIEVDGQTITLASGEFQIYKQDHSAVVALQIEKINNPDKTDSLINQRSFLVSGLGGEHTAFNQLPGGKAEYHGKAFSSDDPNGRLHYSIDFTKKQGYGRIEHLKTPEQNVELAAAELKADEKSHAVILGDTRYGSEEKGTYHLALFGDRAQEIAGSATVKIGEKVHEIGIAGKQ

>Mutant_number:m0021 Master.m0002: fH_V3_P6h2y_WT Mutations: QA122K,SA124Q,LA126R

VAADIGTGLADALTAPLDHKDKGLKSLTLEDSIPQNGTLTLSAQGAEKTFKAGDKDNSLNTGKLKNDKISRFDFVQKIEVDGQTITLASGEFQIYKQNHSAVVALQIEKINNPDKTDSLINKRQFRVSGLGGEHTAFNQLPGGKAEYHGKAFSSDDPNGRLHYSIDFTKKQGYGRIEHLKTLEQNVELAAAELKADEKSHAVILGDTRYGSEEKGTYHLALFGDRAQEIAGSATVKIGEKVHEIGIAGKQ

>Mutant_number:m0022 Master.m0002: fH_V3_P6h2y_WT Mutations: GA210N,EA212A,TA216S

VAADIGTGLADALTAPLDHKDKGLKSLTLEDSIPQNGTLTLSAQGAEKTFKAGDKDNSLNTGKLKNDKISRFDFVQKIEVDGQTITLASGEFQIYKQNHSAVVALQIEKINNPDKTDSLINQRSFLVSGLGGEHTAFNQLPGGKAEYHGKAFSSDDPNGRLHYSIDFTKKQGYGRIEHLKTLEQNVELAAAELKADEKSHAVILGDTRYNSAEKGSYHLALFGDRAQEIAGSATVKIGEKVHEIGIAGKQ

>Mutant_number:m0023 Master.m0002: fH_V3_P6h2y_WT Mutations: PA34S,AA52V,NA98D,LA182P,TA234E,IA237T,GA238V

VAADIGTGLADALTAPLDHKDKGLKSLTLEDSISQNGTLTLSAQGAEKTFKVGDKDNSLNTGKLKNDKISRFDFVQKIEVDGQTITLASGEFQIYKQDHSAVVALQIEKINNPDKTDSLINQRSFLVSGLGGEHTAFNQLPGGKAEYHGKAFSSDDPNGRLHYSIDFTKKQGYGRIEHLKTPEQNVELAAAELKADEKSHAVILGDTRYGSEEKGTYHLALFGDRAQEIAGSAEVKTVEKVHEIGIAGKQ

>Mutant_number:m0024 Master.m0002: fH_V3_P6h2y_WT Mutations: EA239N,KA240G,VA241I,EA243H

VAADIGTGLADALTAPLDHKDKGLKSLTLEDSIPQNGTLTLSAQGAEKTFKAGDKDNSLNTGKLKNDKISRFDFVQKIEVDGQTITLASGEFQIYKQNHSAVVALQIEKINNPDKTDSLINQRSFLVSGLGGEHTAFNQLPGGKAEYHGKAFSSDDPNGRLHYSIDFTKKQGYGRIEHLKTLEQNVELAAAELKADEKSHAVILGDTRYGSEEKGTYHLALFGDRAQEIAGSATVKIGNGIHHIGIAGKQ

>Mutant_number:m0025 Master.m0002: fH_V3_P6h2y_WT Mutations: PA34S,AA52V,NA98D,KA109Q,NA111Q,NA112D,PA113S,DA114E,KA115H,TA116S,IA120V,LA182P

VAADIGTGLADALTAPLDHKDKGLKSLTLEDSISQNGTLTLSAQGAEKTFKVGDKDNSLNTGKLKNDKISRFDFVQKIEVDGQTITLASGEFQIYKQDHSAVVALQIEQIQDSEHSDSLVNQRSFLVSGLGGEHTAFNQLPGGKAEYHGKAFSSDDPNGRLHYSIDFTKKQGYGRIEHLKTPEQNVELAAAELKADEKSHAVILGDTRYGSEEKGTYHLALFGDRAQEIAGSATVKIGEKVHEIGIAGKQ

>Mutant_number:m0026 Master.m0002: fH_V3_P6h2y_WT Mutations: PA34S,AA52V,NA98D,KA109Q,NA111Q,NA112D,PA113S,DA114E,KA115H,TA116S,DA117G,SA118K,LA119M,IA120V,QA122K,SA124Q,LA126R,LA182P

VAADIGTGLADALTAPLDHKDKGLKSLTLEDSISQNGTLTLSAQGAEKTFKVGDKDNSLNTGKLKNDKISRFDFVQKIEVDGQTITLASGEFQIYKQDHSAVVALQIEQIQDSEHSGKMVNKRQFRVSGLGGEHTAFNQLPGGKAEYHGKAFSSDDPNGRLHYSIDFTKKQGYGRIEHLKTPEQNVELAAAELKADEKSHAVILGDTRYGSEEKGTYHLALFGDRAQEIAGSATVKIGEKVHEIGIAGKQ

>Mutant_number:m0027 Master.m0002: fH_V3_P6h2y_WT Mutations: PA34S,AA52V,NA98D,DA114E,KA115H,TA116S,DA117G,SA118K,LA119M,IA120V,LA182P

VAADIGTGLADALTAPLDHKDKGLKSLTLEDSISQNGTLTLSAQGAEKTFKVGDKDNSLNTGKLKNDKISRFDFVQKIEVDGQTITLASGEFQIYKQDHSAVVALQIEKINNPEHSGKMVNQRSFLVSGLGGEHTAFNQLPGGKAEYHGKAFSSDDPNGRLHYSIDFTKKQGYGRIEHLKTPEQNVELAAAELKADEKSHAVILGDTRYGSEEKGTYHLALFGDRAQEIAGSATVKIGEKVHEIGIAGKQ

>Mutant_number:m0028 Master.m0002: fH_V3_P6h2y_WT Mutations: PA34S,AA52V,NA98D,KA109Q,NA111Q,NA112D,PA113S,DA114E,KA115H,TA116S,DA117G,SA118K,LA119M,IA120V,LA182P,EA239N,KA240G,VA241I,EA243H

VAADIGTGLADALTAPLDHKDKGLKSLTLEDSISQNGTLTLSAQGAEKTFKVGDKDNSLNTGKLKNDKISRFDFVQKIEVDGQTITLASGEFQIYKQDHSAVVALQIEQIQDSEHSGKMVNQRSFLVSGLGGEHTAFNQLPGGKAEYHGKAFSSDDPNGRLHYSIDFTKKQGYGRIEHLKTPEQNVELAAAELKADEKSHAVILGDTRYGSEEKGTYHLALFGDRAQEIAGSATVKIGNGIHHIGIAGKQ

>Mutant_number:m0029 Master.m0002: fH_V3_P6h2y_WT Mutations: PA34S,AA52V,NA98D,KA109Q,NA111Q,NA112D,PA113S,DA114E,KA115H,TA116S,DA117G,SA118K,LA119M,IA120V,LA182P,GA210N,EA212A,TA216S

VAADIGTGLADALTAPLDHKDKGLKSLTLEDSISQNGTLTLSAQGAEKTFKVGDKDNSLNTGKLKNDKISRFDFVQKIEVDGQTITLASGEFQIYKQDHSAVVALQIEQIQDSEHSGKMVNQRSFLVSGLGGEHTAFNQLPGGKAEYHGKAFSSDDPNGRLHYSIDFTKKQGYGRIEHLKTPEQNVELAAAELKADEKSHAVILGDTRYNSAEKGSYHLALFGDRAQEIAGSATVKIGEKVHEIGIAGKQ

>Mutant_number:m0030 Master.m0002: fH_V3_P6h2y_WT Mutations: PA34S,AA52V,NA98D,KA109Q,NA111Q,NA112D,PA113S,DA114E,KA115H,TA116S,DA117G,SA118K,LA119M,IA120V,LA182P,TA234E,IA237T,GA238V

VAADIGTGLADALTAPLDHKDKGLKSLTLEDSISQNGTLTLSAQGAEKTFKVGDKDNSLNTGKLKNDKISRFDFVQKIEVDGQTITLASGEFQIYKQDHSAVVALQIEQIQDSEHSGKMVNQRSFLVSGLGGEHTAFNQLPGGKAEYHGKAFSSDDPNGRLHYSIDFTKKQGYGRIEHLKTPEQNVELAAAELKADEKSHAVILGDTRYGSEEKGTYHLALFGDRAQEIAGSAEVKTVEKVHEIGIAGKQ

>Mutant_number:m0031 Master.m0002: fH_V3_P6h2y_WT Mutations: PA34R,GA37E,AA52V,NA98D,KA109Q,NA111Q,NA112D,PA113S,DA114E,KA115H,TA116S,DA117G,SA118K,LA119M,IA120V,LA182P

VAADIGTGLADALTAPLDHKDKGLKSLTLEDSIRQNETLTLSAQGAEKTFKVGDKDNSLNTGKLKNDKISRFDFVQKIEVDGQTITLASGEFQIYKQDHSAVVALQIEQIQDSEHSGKMVNQRSFLVSGLGGEHTAFNQLPGGKAEYHGKAFSSDDPNGRLHYSIDFTKKQGYGRIEHLKTPEQNVELAAAELKADEKSHAVILGDTRYGSEEKGTYHLALFGDRAQEIAGSATVKIGEKVHEIGIAGKQ

>Mutant_number:m0032 Master.m0002: fH_V3_P6h2y_WT Mutations: PA34S,KA109Q,NA111Q,NA112D,PA113S,DA114E,KA115H,TA116S,DA117G,SA118K,LA119M,IA120V,QA122K,SA124Q,LA126R,VA127I,GA210N,EA212A,TA216S,TA234E,IA237T,GA238V,EA239N,KA240G,VA241I,EA243H

VAADIGTGLADALTAPLDHKDKGLKSLTLEDSISQNGTLTLSAQGAEKTFKAGDKDNSLNTGKLKNDKISRFDFVQKIEVDGQTITLASGEFQIYKQNHSAVVALQIEQIQDSEHSGKMVNKRQFRISGLGGEHTAFNQLPGGKAEYHGKAFSSDDPNGRLHYSIDFTKKQGYGRIEHLKTLEQNVELAAAELKADEKSHAVILGDTRYNSAEKGSYHLALFGDRAQEIAGSAEVKTVNGIHHIGIAGKQ

>Mutant_number:m0033 Master.m0002: fH_V3_P6h2y_WT Mutations: PA34R,AA52V,KA109Q,NA111Q,NA112D,PA113S,DA114E,KA115H,TA116S,DA117G,SA118K,LA119M,IA120V,QA122K,SA124Q,LA126R,VA127I,GA210N,EA212A,TA216S,TA234E,IA237T,GA238V,EA239N,KA240G,VA241I,EA243H

VAADIGTGLADALTAPLDHKDKGLKSLTLEDSIRQNGTLTLSAQGAEKTFKVGDKDNSLNTGKLKNDKISRFDFVQKIEVDGQTITLASGEFQIYKQNHSAVVALQIEQIQDSEHSGKMVNKRQFRISGLGGEHTAFNQLPGGKAEYHGKAFSSDDPNGRLHYSIDFTKKQGYGRIEHLKTLEQNVELAAAELKADEKSHAVILGDTRYNSAEKGSYHLALFGDRAQEIAGSAEVKTVNGIHHIGIAGKQ

>Mutant_number:m0034 Master.m0002: fH_V3_P6h2y_WT Mutations: PA34R,NA98D,KA109Q,NA111Q,NA112D,PA113S,DA114E,KA115H,TA116S,DA117G,SA118K,LA119M,IA120V,QA122K,SA124Q,LA126R,VA127I,GA210N,EA212A,TA216S,TA234E,IA237T,GA238V,EA239N,KA240G,VA241I,EA243H

VAADIGTGLADALTAPLDHKDKGLKSLTLEDSIRQNGTLTLSAQGAEKTFKAGDKDNSLNTGKLKNDKISRFDFVQKIEVDGQTITLASGEFQIYKQDHSAVVALQIEQIQDSEHSGKMVNKRQFRISGLGGEHTAFNQLPGGKAEYHGKAFSSDDPNGRLHYSIDFTKKQGYGRIEHLKTLEQNVELAAAELKADEKSHAVILGDTRYNSAEKGSYHLALFGDRAQEIAGSAEVKTVNGIHHIGIAGKQ

>Mutant_number:m0035 Master.m0002: fH_V3_P6h2y_WT Mutations: PA34R,PA113S,DA114E,KA115H,TA116S,DA117G,SA118K,LA119M,IA120V,QA122K,SA124Q,LA126R,VA127I,GA210N,EA212A,TA216S,TA234E,IA237T,GA238V,EA239N,KA240G,VA241I,EA243H

VAADIGTGLADALTAPLDHKDKGLKSLTLEDSIRQNGTLTLSAQGAEKTFKAGDKDNSLNTGKLKNDKISRFDFVQKIEVDGQTITLASGEFQIYKQNHSAVVALQIEKINNSEHSGKMVNKRQFRISGLGGEHTAFNQLPGGKAEYHGKAFSSDDPNGRLHYSIDFTKKQGYGRIEHLKTLEQNVELAAAELKADEKSHAVILGDTRYNSAEKGSYHLALFGDRAQEIAGSAEVKTVNGIHHIGIAGKQ

>Mutant_number:m0036 Master.m0002: fH_V3_P6h2y_WT Mutations: PA34R,KA109Q,NA111Q,NA112D,TA116S,DA117G,SA118K,LA119M,IA120V,QA122K,SA124Q,LA126R,VA127I,GA210N,EA212A,TA216S,TA234E,IA237T,GA238V,EA239N,KA240G,VA241I,EA243H

VAADIGTGLADALTAPLDHKDKGLKSLTLEDSIRQNGTLTLSAQGAEKTFKAGDKDNSLNTGKLKNDKISRFDFVQKIEVDGQTITLASGEFQIYKQNHSAVVALQIEQIQDPDKSGKMVNKRQFRISGLGGEHTAFNQLPGGKAEYHGKAFSSDDPNGRLHYSIDFTKKQGYGRIEHLKTLEQNVELAAAELKADEKSHAVILGDTRYNSAEKGSYHLALFGDRAQEIAGSAEVKTVNGIHHIGIAGKQ

>Mutant_number:m0037 Master.m0002: fH_V3_P6h2y_WT Mutations: PA34R,KA109Q,NA111Q,NA112D,PA113S,DA114E,KA115H,LA119M,IA120V,QA122K,SA124Q,LA126R,VA127I,GA210N,EA212A,TA216S,TA234E,IA237T,GA238V,EA239N,KA240G,VA241I,EA243H

VAADIGTGLADALTAPLDHKDKGLKSLTLEDSIRQNGTLTLSAQGAEKTFKAGDKDNSLNTGKLKNDKISRFDFVQKIEVDGQTITLASGEFQIYKQNHSAVVALQIEQIQDSEHTDSMVNKRQFRISGLGGEHTAFNQLPGGKAEYHGKAFSSDDPNGRLHYSIDFTKKQGYGRIEHLKTLEQNVELAAAELKADEKSHAVILGDTRYNSAEKGSYHLALFGDRAQEIAGSAEVKTVNGIHHIGIAGKQ

>Mutant_number:m0038 Master.m0002: fH_V3_P6h2y_WT Mutations: PA34R,KA109Q,NA111Q,NA112D,PA113S,DA114E,KA115H,TA116S,DA117G,SA118K,SA124Q,LA126R,VA127I,GA210N,EA212A,TA216S,TA234E,IA237T,GA238V,EA239N,KA240G,VA241I,EA243H

VAADIGTGLADALTAPLDHKDKGLKSLTLEDSIRQNGTLTLSAQGAEKTFKAGDKDNSLNTGKLKNDKISRFDFVQKIEVDGQTITLASGEFQIYKQNHSAVVALQIEQIQDSEHSGKLINQRQFRISGLGGEHTAFNQLPGGKAEYHGKAFSSDDPNGRLHYSIDFTKKQGYGRIEHLKTLEQNVELAAAELKADEKSHAVILGDTRYNSAEKGSYHLALFGDRAQEIAGSAEVKTVNGIHHIGIAGKQ

>Mutant_number:m0039 Master.m0002: fH_V3_P6h2y_WT Mutations: PA34R,KA109Q,NA111Q,NA112D,PA113S,DA114E,KA115H,TA116S,DA117G,SA118K,LA119M,IA120V,QA122K,GA210N,EA212A,TA216S,TA234E,IA237T,GA238V,EA239N,KA240G,VA241I,EA243H

VAADIGTGLADALTAPLDHKDKGLKSLTLEDSIRQNGTLTLSAQGAEKTFKAGDKDNSLNTGKLKNDKISRFDFVQKIEVDGQTITLASGEFQIYKQNHSAVVALQIEQIQDSEHSGKMVNKRSFLVSGLGGEHTAFNQLPGGKAEYHGKAFSSDDPNGRLHYSIDFTKKQGYGRIEHLKTLEQNVELAAAELKADEKSHAVILGDTRYNSAEKGSYHLALFGDRAQEIAGSAEVKTVNGIHHIGIAGKQ

>Mutant_number:m0040 Master.m0002: fH_V3_P6h2y_WT Mutations: PA34R,KA109Q,NA111Q,NA112D,PA113S,DA114E,KA115H,TA116S,DA117G,SA118K,LA119M,IA120V,QA122K,SA124Q,LA126R,VA127I,LA182P,GA210N,EA212A,TA216S,TA234E,IA237T,GA238V,EA239N,KA240G,VA241I,EA243H

VAADIGTGLADALTAPLDHKDKGLKSLTLEDSIRQNGTLTLSAQGAEKTFKAGDKDNSLNTGKLKNDKISRFDFVQKIEVDGQTITLASGEFQIYKQNHSAVVALQIEQIQDSEHSGKMVNKRQFRISGLGGEHTAFNQLPGGKAEYHGKAFSSDDPNGRLHYSIDFTKKQGYGRIEHLKTPEQNVELAAAELKADEKSHAVILGDTRYNSAEKGSYHLALFGDRAQEIAGSAEVKTVNGIHHIGIAGKQ

>Mutant_number:m0041 Master.m0002: fH_V3_P6h2y_WT Mutations: PA34R,KA109Q,NA111Q,NA112D,PA113S,DA114E,KA115H,TA116S,DA117G,SA118K,LA119M,IA120V,QA122K,SA124Q,LA126R,VA127I,TA234E,IA237T,GA238V,EA239N,KA240G,VA241I,EA243H

VAADIGTGLADALTAPLDHKDKGLKSLTLEDSIRQNGTLTLSAQGAEKTFKAGDKDNSLNTGKLKNDKISRFDFVQKIEVDGQTITLASGEFQIYKQNHSAVVALQIEQIQDSEHSGKMVNKRQFRISGLGGEHTAFNQLPGGKAEYHGKAFSSDDPNGRLHYSIDFTKKQGYGRIEHLKTLEQNVELAAAELKADEKSHAVILGDTRYGSEEKGTYHLALFGDRAQEIAGSAEVKTVNGIHHIGIAGKQ

>Mutant_number:m0042 Master.m0002: fH_V3_P6h2y_WT Mutations: PA34R,KA109Q,NA111Q,NA112D,PA113S,DA114E,KA115H,TA116S,DA117G,SA118K,LA119M,IA120V,QA122K,SA124Q,LA126R,VA127I,GA210N,EA212A,TA216S,EA239N,KA240G,VA241I,EA243H

VAADIGTGLADALTAPLDHKDKGLKSLTLEDSIRQNGTLTLSAQGAEKTFKAGDKDNSLNTGKLKNDKISRFDFVQKIEVDGQTITLASGEFQIYKQNHSAVVALQIEQIQDSEHSGKMVNKRQFRISGLGGEHTAFNQLPGGKAEYHGKAFSSDDPNGRLHYSIDFTKKQGYGRIEHLKTLEQNVELAAAELKADEKSHAVILGDTRYNSAEKGSYHLALFGDRAQEIAGSATVKIGNGIHHIGIAGKQ

>Mutant_number:m0043 Master.m0002: fH_V3_P6h2y_WT Mutations: PA34R,KA109Q,NA111Q,NA112D,PA113S,DA114E,KA115H,TA116S,DA117G,SA118K,LA119M,IA120V,QA122K,SA124Q,LA126R,VA127I,GA210N,EA212A,TA216S,TA234E,IA237T,GA238V,EA243H

VAADIGTGLADALTAPLDHKDKGLKSLTLEDSIRQNGTLTLSAQGAEKTFKAGDKDNSLNTGKLKNDKISRFDFVQKIEVDGQTITLASGEFQIYKQNHSAVVALQIEQIQDSEHSGKMVNKRQFRISGLGGEHTAFNQLPGGKAEYHGKAFSSDDPNGRLHYSIDFTKKQGYGRIEHLKTLEQNVELAAAELKADEKSHAVILGDTRYNSAEKGSYHLALFGDRAQEIAGSAEVKTVEKVHHIGIAGKQ

>Mutant_number:m0044 Master.m0002: fH_V3_P6h2y_WT Mutations: PA34R,KA109Q,NA111Q,NA112D,PA113S,DA114E,KA115H,TA116S,DA117G,SA118K,LA119M,IA120V,QA122K,SA124Q,LA126R,VA127I,GA210N,EA212A,TA216S,TA234E,IA237T,GA238V,EA239N,KA240G

VAADIGTGLADALTAPLDHKDKGLKSLTLEDSIRQNGTLTLSAQGAEKTFKAGDKDNSLNTGKLKNDKISRFDFVQKIEVDGQTITLASGEFQIYKQNHSAVVALQIEQIQDSEHSGKMVNKRQFRISGLGGEHTAFNQLPGGKAEYHGKAFSSDDPNGRLHYSIDFTKKQGYGRIEHLKTLEQNVELAAAELKADEKSHAVILGDTRYNSAEKGSYHLALFGDRAQEIAGSAEVKTVNGVHEIGIAGKQ

>Mutant_number:m0045 Master.m0002: fH_V3_P6h2y_WT Mutations: PA34R,KA109Q,NA111Q,NA112D,PA113S,DA114E,KA115H,TA116S,DA117G,SA118K,LA119M,IA120V,QA122K,SA124Q,GA210N,EA212A,TA216S,TA234E,IA237T,GA238V,EA239N,KA240G,VA241I,EA243H

VAADIGTGLADALTAPLDHKDKGLKSLTLEDSIRQNGTLTLSAQGAEKTFKAGDKDNSLNTGKLKNDKISRFDFVQKIEVDGQTITLASGEFQIYKQNHSAVVALQIEQIQDSEHSGKMVNKRQFLVSGLGGEHTAFNQLPGGKAEYHGKAFSSDDPNGRLHYSIDFTKKQGYGRIEHLKTLEQNVELAAAELKADEKSHAVILGDTRYNSAEKGSYHLALFGDRAQEIAGSAEVKTVNGIHHIGIAGKQ

>Mutant_number:m0046 Master.m0002: fH_V3_P6h2y_WT Mutations: PA34R,KA109Q,NA111Q,NA112D,PA113S,DA114E,KA115H,TA116S,IA120V,QA122K,SA124Q,LA126R,VA127I,GA210N,EA212A,TA216S,TA234E,IA237T,GA238V,EA239N,KA240G,VA241I,EA243H

VAADIGTGLADALTAPLDHKDKGLKSLTLEDSIRQNGTLTLSAQGAEKTFKAGDKDNSLNTGKLKNDKISRFDFVQKIEVDGQTITLASGEFQIYKQNHSAVVALQIEQIQDSEHSDSLVNKRQFRISGLGGEHTAFNQLPGGKAEYHGKAFSSDDPNGRLHYSIDFTKKQGYGRIEHLKTLEQNVELAAAELKADEKSHAVILGDTRYNSAEKGSYHLALFGDRAQEIAGSAEVKTVNGIHHIGIAGKQ

>Mutant_number:m0047 Master.m0002: fH_V3_P6h2y_WT Mutations: PA34S,NA111Q,NA112D,PA113S,DA114E,KA115H,TA116S,DA117G,SA118K,IA120V,QA122K,SA124Q,LA126R,VA127I,EA212A,TA216S,TA234E,IA237T,KA240G,VA241I,EA243H

VAADIGTGLADALTAPLDHKDKGLKSLTLEDSISQNGTLTLSAQGAEKTFKAGDKDNSLNTGKLKNDKISRFDFVQKIEVDGQTITLASGEFQIYKQNHSAVVALQIEKIQDSEHSGKLVNKRQFRISGLGGEHTAFNQLPGGKAEYHGKAFSSDDPNGRLHYSIDFTKKQGYGRIEHLKTLEQNVELAAAELKADEKSHAVILGDTRYGSAEKGSYHLALFGDRAQEIAGSAEVKTGEGIHHIGIAGKQ

>Mutant_number:m0048 Master.m0002: fH_V3_P6h2y_WT Mutations: PA34R,AA52V,NA111Q,NA112D,PA113S,DA114E,KA115H,TA116S,DA117G,SA118K,IA120V,QA122K,SA124Q,LA126R,VA127I,EA212A,TA216S,TA234E,IA237T,KA240G,VA241I,EA243H

VAADIGTGLADALTAPLDHKDKGLKSLTLEDSIRQNGTLTLSAQGAEKTFKVGDKDNSLNTGKLKNDKISRFDFVQKIEVDGQTITLASGEFQIYKQNHSAVVALQIEKIQDSEHSGKLVNKRQFRISGLGGEHTAFNQLPGGKAEYHGKAFSSDDPNGRLHYSIDFTKKQGYGRIEHLKTLEQNVELAAAELKADEKSHAVILGDTRYGSAEKGSYHLALFGDRAQEIAGSAEVKTGEGIHHIGIAGKQ

>Mutant_number:m0049 Master.m0002: fH_V3_P6h2y_WT Mutations: PA34R,NA98D,NA111Q,NA112D,PA113S,DA114E,KA115H,TA116S,DA117G,SA118K,IA120V,QA122K,SA124Q,LA126R,VA127I,EA212A,TA216S,TA234E,IA237T,KA240G,VA241I,EA243H

VAADIGTGLADALTAPLDHKDKGLKSLTLEDSIRQNGTLTLSAQGAEKTFKAGDKDNSLNTGKLKNDKISRFDFVQKIEVDGQTITLASGEFQIYKQDHSAVVALQIEKIQDSEHSGKLVNKRQFRISGLGGEHTAFNQLPGGKAEYHGKAFSSDDPNGRLHYSIDFTKKQGYGRIEHLKTLEQNVELAAAELKADEKSHAVILGDTRYGSAEKGSYHLALFGDRAQEIAGSAEVKTGEGIHHIGIAGKQ

>Mutant_number:m0050 Master.m0002: fH_V3_P6h2y_WT Mutations: PA34R,PA113S,DA114E,KA115H,TA116S,DA117G,SA118K,IA120V,QA122K,SA124Q,LA126R,VA127I,EA212A,TA216S,TA234E,IA237T,KA240G,VA241I,EA243H

VAADIGTGLADALTAPLDHKDKGLKSLTLEDSIRQNGTLTLSAQGAEKTFKAGDKDNSLNTGKLKNDKISRFDFVQKIEVDGQTITLASGEFQIYKQNHSAVVALQIEKINNSEHSGKLVNKRQFRISGLGGEHTAFNQLPGGKAEYHGKAFSSDDPNGRLHYSIDFTKKQGYGRIEHLKTLEQNVELAAAELKADEKSHAVILGDTRYGSAEKGSYHLALFGDRAQEIAGSAEVKTGEGIHHIGIAGKQ

>Mutant_number:m0051 Master.m0002: fH_V3_P6h2y_WT Mutations: PA34R,NA111Q,NA112D,KA115H,TA116S,DA117G,SA118K,IA120V,QA122K,SA124Q,LA126R,VA127I,EA212A,TA216S,TA234E,IA237T,KA240G,VA241I,EA243H

VAADIGTGLADALTAPLDHKDKGLKSLTLEDSIRQNGTLTLSAQGAEKTFKAGDKDNSLNTGKLKNDKISRFDFVQKIEVDGQTITLASGEFQIYKQNHSAVVALQIEKIQDPDHSGKLVNKRQFRISGLGGEHTAFNQLPGGKAEYHGKAFSSDDPNGRLHYSIDFTKKQGYGRIEHLKTLEQNVELAAAELKADEKSHAVILGDTRYGSAEKGSYHLALFGDRAQEIAGSAEVKTGEGIHHIGIAGKQ

>Mutant_number:m0052 Master.m0002: fH_V3_P6h2y_WT Mutations: PA34R,NA111Q,NA112D,PA113S,DA114E,DA117G,SA118K,IA120V,QA122K,SA124Q,LA126R,VA127I,EA212A,TA216S,TA234E,IA237T,KA240G,VA241I,EA243H

VAADIGTGLADALTAPLDHKDKGLKSLTLEDSIRQNGTLTLSAQGAEKTFKAGDKDNSLNTGKLKNDKISRFDFVQKIEVDGQTITLASGEFQIYKQNHSAVVALQIEKIQDSEKTGKLVNKRQFRISGLGGEHTAFNQLPGGKAEYHGKAFSSDDPNGRLHYSIDFTKKQGYGRIEHLKTLEQNVELAAAELKADEKSHAVILGDTRYGSAEKGSYHLALFGDRAQEIAGSAEVKTGEGIHHIGIAGKQ

>Mutant_number:m0053 Master.m0002: fH_V3_P6h2y_WT Mutations: PA34R,NA111Q,NA112D,PA113S,DA114E,KA115H,TA116S,IA120V,QA122K,SA124Q,LA126R,VA127I,EA212A,TA216S,TA234E,IA237T,KA240G,VA241I,EA243H

VAADIGTGLADALTAPLDHKDKGLKSLTLEDSIRQNGTLTLSAQGAEKTFKAGDKDNSLNTGKLKNDKISRFDFVQKIEVDGQTITLASGEFQIYKQNHSAVVALQIEKIQDSEHSDSLVNKRQFRISGLGGEHTAFNQLPGGKAEYHGKAFSSDDPNGRLHYSIDFTKKQGYGRIEHLKTLEQNVELAAAELKADEKSHAVILGDTRYGSAEKGSYHLALFGDRAQEIAGSAEVKTGEGIHHIGIAGKQ

>Mutant_number:m0054 Master.m0002: fH_V3_P6h2y_WT Mutations: PA34R,NA111Q,NA112D,PA113S,DA114E,KA115H,TA116S,DA117G,SA118K,SA124Q,LA126R,VA127I,EA212A,TA216S,TA234E,IA237T,KA240G,VA241I,EA243H

VAADIGTGLADALTAPLDHKDKGLKSLTLEDSIRQNGTLTLSAQGAEKTFKAGDKDNSLNTGKLKNDKISRFDFVQKIEVDGQTITLASGEFQIYKQNHSAVVALQIEKIQDSEHSGKLINQRQFRISGLGGEHTAFNQLPGGKAEYHGKAFSSDDPNGRLHYSIDFTKKQGYGRIEHLKTLEQNVELAAAELKADEKSHAVILGDTRYGSAEKGSYHLALFGDRAQEIAGSAEVKTGEGIHHIGIAGKQ

>Mutant_number:m0055 Master.m0002: fH_V3_P6h2y_WT Mutations: PA34R,NA111Q,NA112D,PA113S,DA114E,KA115H,TA116S,DA117G,SA118K,IA120V,QA122K,VA127I,EA212A,TA216S,TA234E,IA237T,KA240G,VA241I,EA243H

VAADIGTGLADALTAPLDHKDKGLKSLTLEDSIRQNGTLTLSAQGAEKTFKAGDKDNSLNTGKLKNDKISRFDFVQKIEVDGQTITLASGEFQIYKQNHSAVVALQIEKIQDSEHSGKLVNKRSFLISGLGGEHTAFNQLPGGKAEYHGKAFSSDDPNGRLHYSIDFTKKQGYGRIEHLKTLEQNVELAAAELKADEKSHAVILGDTRYGSAEKGSYHLALFGDRAQEIAGSAEVKTGEGIHHIGIAGKQ

>Mutant_number:m0056 Master.m0002: fH_V3_P6h2y_WT Mutations: PA34R,NA111Q,NA112D,PA113S,DA114E,KA115H,TA116S,DA117G,SA118K,IA120V,QA122K,SA124Q,LA126R,VA127I,LA182P,EA212A,TA216S,TA234E,IA237T,KA240G,VA241I,EA243H

VAADIGTGLADALTAPLDHKDKGLKSLTLEDSIRQNGTLTLSAQGAEKTFKAGDKDNSLNTGKLKNDKISRFDFVQKIEVDGQTITLASGEFQIYKQNHSAVVALQIEKIQDSEHSGKLVNKRQFRISGLGGEHTAFNQLPGGKAEYHGKAFSSDDPNGRLHYSIDFTKKQGYGRIEHLKTPEQNVELAAAELKADEKSHAVILGDTRYGSAEKGSYHLALFGDRAQEIAGSAEVKTGEGIHHIGIAGKQ

>Mutant_number:m0057 Master.m0002: fH_V3_P6h2y_WT Mutations: PA34R,NA111Q,NA112D,PA113S,DA114E,KA115H,TA116S,DA117G,SA118K,IA120V,QA122K,SA124Q,LA126R,VA127I,TA234E,IA237T,KA240G,VA241I,EA243H

VAADIGTGLADALTAPLDHKDKGLKSLTLEDSIRQNGTLTLSAQGAEKTFKAGDKDNSLNTGKLKNDKISRFDFVQKIEVDGQTITLASGEFQIYKQNHSAVVALQIEKIQDSEHSGKLVNKRQFRISGLGGEHTAFNQLPGGKAEYHGKAFSSDDPNGRLHYSIDFTKKQGYGRIEHLKTLEQNVELAAAELKADEKSHAVILGDTRYGSEEKGTYHLALFGDRAQEIAGSAEVKTGEGIHHIGIAGKQ

>Mutant_number:m0058 Master.m0002: fH_V3_P6h2y_WT Mutations: PA34R,NA111Q,NA112D,PA113S,DA114E,KA115H,TA116S,DA117G,SA118K,IA120V,QA122K,SA124Q,LA126R,VA127I,EA212A,TA216S,KA240G,VA241I,EA243H

VAADIGTGLADALTAPLDHKDKGLKSLTLEDSIRQNGTLTLSAQGAEKTFKAGDKDNSLNTGKLKNDKISRFDFVQKIEVDGQTITLASGEFQIYKQNHSAVVALQIEKIQDSEHSGKLVNKRQFRISGLGGEHTAFNQLPGGKAEYHGKAFSSDDPNGRLHYSIDFTKKQGYGRIEHLKTLEQNVELAAAELKADEKSHAVILGDTRYGSAEKGSYHLALFGDRAQEIAGSATVKIGEGIHHIGIAGKQ

>Mutant_number:m0059 Master.m0002: fH_V3_P6h2y_WT Mutations: PA34R,NA111Q,NA112D,PA113S,DA114E,KA115H,TA116S,DA117G,SA118K,IA120V,QA122K,SA124Q,LA126R,VA127I,EA212A,TA216S,TA234E,IA237T,VA241I,EA243H

VAADIGTGLADALTAPLDHKDKGLKSLTLEDSIRQNGTLTLSAQGAEKTFKAGDKDNSLNTGKLKNDKISRFDFVQKIEVDGQTITLASGEFQIYKQNHSAVVALQIEKIQDSEHSGKLVNKRQFRISGLGGEHTAFNQLPGGKAEYHGKAFSSDDPNGRLHYSIDFTKKQGYGRIEHLKTLEQNVELAAAELKADEKSHAVILGDTRYGSAEKGSYHLALFGDRAQEIAGSAEVKTGEKIHHIGIAGKQ

>Mutant_number:m0060 Master.m0002: fH_V3_P6h2y_WT Mutations: PA34R,NA111Q,NA112D,PA113S,DA114E,KA115H,TA116S,DA117G,SA118K,IA120V,QA122K,SA124Q,LA126R,VA127I,EA212A,TA216S,TA234E,IA237T,KA240G,EA243H

VAADIGTGLADALTAPLDHKDKGLKSLTLEDSIRQNGTLTLSAQGAEKTFKAGDKDNSLNTGKLKNDKISRFDFVQKIEVDGQTITLASGEFQIYKQNHSAVVALQIEKIQDSEHSGKLVNKRQFRISGLGGEHTAFNQLPGGKAEYHGKAFSSDDPNGRLHYSIDFTKKQGYGRIEHLKTLEQNVELAAAELKADEKSHAVILGDTRYGSAEKGSYHLALFGDRAQEIAGSAEVKTGEGVHHIGIAGKQ

>Mutant_number:m0061 Master.m0002: fH_V3_P6h2y_WT Mutations: PA34R,NA111Q,NA112D,PA113S,DA114E,KA115H,TA116S,DA117G,SA118K,IA120V,QA122K,SA124Q,LA126R,VA127I,EA212A,TA216S,TA234E,IA237T,KA240G,VA241I

VAADIGTGLADALTAPLDHKDKGLKSLTLEDSIRQNGTLTLSAQGAEKTFKAGDKDNSLNTGKLKNDKISRFDFVQKIEVDGQTITLASGEFQIYKQNHSAVVALQIEKIQDSEHSGKLVNKRQFRISGLGGEHTAFNQLPGGKAEYHGKAFSSDDPNGRLHYSIDFTKKQGYGRIEHLKTLEQNVELAAAELKADEKSHAVILGDTRYGSAEKGSYHLALFGDRAQEIAGSAEVKTGEGIHEIGIAGKQ

>Mutant_number:m0062 Master.m0002: fH_V3_P6h2y_WT Mutations: PA34S,KA109E,NA111Q,NA112D,PA113S,DA114E,KA115H,TA116S,DA117G,SA118K,LA119A,IA120V,QA122K,SA124Q,LA126R,VA127I,GA210D,EA212A,TA216S,TA234E,IA237T,GA238A,EA239D,KA240G,VA241I,EA243H

VAADIGTGLADALTAPLDHKDKGLKSLTLEDSISQNGTLTLSAQGAEKTFKAGDKDNSLNTGKLKNDKISRFDFVQKIEVDGQTITLASGEFQIYKQNHSAVVALQIEEIQDSEHSGKAVNKRQFRISGLGGEHTAFNQLPGGKAEYHGKAFSSDDPNGRLHYSIDFTKKQGYGRIEHLKTLEQNVELAAAELKADEKSHAVILGDTRYDSAEKGSYHLALFGDRAQEIAGSAEVKTADGIHHIGIAGKQ

>Mutant_number:m0063 Master.m0002: fH_V3_P6h2y_WT Mutations: PA34R,AA52V,KA109E,NA111Q,NA112D,PA113S,DA114E,KA115H,TA116S,DA117G,SA118K,LA119A,IA120V,QA122K,SA124Q,LA126R,VA127I,GA210D,EA212A,TA216S,TA234E,IA237T,GA238A,EA239D,KA240G,VA241I,EA243H

VAADIGTGLADALTAPLDHKDKGLKSLTLEDSIRQNGTLTLSAQGAEKTFKVGDKDNSLNTGKLKNDKISRFDFVQKIEVDGQTITLASGEFQIYKQNHSAVVALQIEEIQDSEHSGKAVNKRQFRISGLGGEHTAFNQLPGGKAEYHGKAFSSDDPNGRLHYSIDFTKKQGYGRIEHLKTLEQNVELAAAELKADEKSHAVILGDTRYDSAEKGSYHLALFGDRAQEIAGSAEVKTADGIHHIGIAGKQ

>Mutant_number:m0064 Master.m0002: fH_V3_P6h2y_WT Mutations: PA34R,NA98D,KA109E,NA111Q,NA112D,PA113S,DA114E,KA115H,TA116S,DA117G,SA118K,LA119A,IA120V,QA122K,SA124Q,LA126R,VA127I,GA210D,EA212A,TA216S,TA234E,IA237T,GA238A,EA239D,KA240G,VA241I,EA243H

VAADIGTGLADALTAPLDHKDKGLKSLTLEDSIRQNGTLTLSAQGAEKTFKAGDKDNSLNTGKLKNDKISRFDFVQKIEVDGQTITLASGEFQIYKQDHSAVVALQIEEIQDSEHSGKAVNKRQFRISGLGGEHTAFNQLPGGKAEYHGKAFSSDDPNGRLHYSIDFTKKQGYGRIEHLKTLEQNVELAAAELKADEKSHAVILGDTRYDSAEKGSYHLALFGDRAQEIAGSAEVKTADGIHHIGIAGKQ

>Mutant_number:m0065 Master.m0002: fH_V3_P6h2y_WT Mutations: PA34R,NA112D,PA113S,DA114E,KA115H,TA116S,DA117G,SA118K,LA119A,IA120V,QA122K,SA124Q,LA126R,VA127I,GA210D,EA212A,TA216S,TA234E,IA237T,GA238A,EA239D,KA240G,VA241I,EA243H

VAADIGTGLADALTAPLDHKDKGLKSLTLEDSIRQNGTLTLSAQGAEKTFKAGDKDNSLNTGKLKNDKISRFDFVQKIEVDGQTITLASGEFQIYKQNHSAVVALQIEKINDSEHSGKAVNKRQFRISGLGGEHTAFNQLPGGKAEYHGKAFSSDDPNGRLHYSIDFTKKQGYGRIEHLKTLEQNVELAAAELKADEKSHAVILGDTRYDSAEKGSYHLALFGDRAQEIAGSAEVKTADGIHHIGIAGKQ

>Mutant_number:m0066 Master.m0002: fH_V3_P6h2y_WT Mutations: PA34R,KA109E,NA111Q,DA114E,KA115H,TA116S,DA117G,SA118K,LA119A,IA120V,QA122K,SA124Q,LA126R,VA127I,GA210D,EA212A,TA216S,TA234E,IA237T,GA238A,EA239D,KA240G,VA241I,EA243H

VAADIGTGLADALTAPLDHKDKGLKSLTLEDSIRQNGTLTLSAQGAEKTFKAGDKDNSLNTGKLKNDKISRFDFVQKIEVDGQTITLASGEFQIYKQNHSAVVALQIEEIQNPEHSGKAVNKRQFRISGLGGEHTAFNQLPGGKAEYHGKAFSSDDPNGRLHYSIDFTKKQGYGRIEHLKTLEQNVELAAAELKADEKSHAVILGDTRYDSAEKGSYHLALFGDRAQEIAGSAEVKTADGIHHIGIAGKQ

>Mutant_number:m0067 Master.m0002: fH_V3_P6h2y_WT Mutations: PA34R,KA109E,NA111Q,NA112D,PA113S,TA116S,DA117G,SA118K,LA119A,IA120V,QA122K,SA124Q,LA126R,VA127I,GA210D,EA212A,TA216S,TA234E,IA237T,GA238A,EA239D,KA240G,VA241I,EA243H

VAADIGTGLADALTAPLDHKDKGLKSLTLEDSIRQNGTLTLSAQGAEKTFKAGDKDNSLNTGKLKNDKISRFDFVQKIEVDGQTITLASGEFQIYKQNHSAVVALQIEEIQDSDKSGKAVNKRQFRISGLGGEHTAFNQLPGGKAEYHGKAFSSDDPNGRLHYSIDFTKKQGYGRIEHLKTLEQNVELAAAELKADEKSHAVILGDTRYDSAEKGSYHLALFGDRAQEIAGSAEVKTADGIHHIGIAGKQ

>Mutant_number:m0068 Master.m0002: fH_V3_P6h2y_WT Mutations: PA34R,KA109E,NA111Q,NA112D,PA113S,DA114E,KA115H,SA118K,LA119A,IA120V,QA122K,SA124Q,LA126R,VA127I,GA210D,EA212A,TA216S,TA234E,IA237T,GA238A,EA239D,KA240G,VA241I,EA243H

VAADIGTGLADALTAPLDHKDKGLKSLTLEDSIRQNGTLTLSAQGAEKTFKAGDKDNSLNTGKLKNDKISRFDFVQKIEVDGQTITLASGEFQIYKQNHSAVVALQIEEIQDSEHTDKAVNKRQFRISGLGGEHTAFNQLPGGKAEYHGKAFSSDDPNGRLHYSIDFTKKQGYGRIEHLKTLEQNVELAAAELKADEKSHAVILGDTRYDSAEKGSYHLALFGDRAQEIAGSAEVKTADGIHHIGIAGKQ

>Mutant_number:m0069 Master.m0002: fH_V3_P6h2y_WT Mutations: PA34R,KA109E,NA111Q,NA112D,PA113S,DA114E,KA115H,TA116S,DA117G,IA120V,QA122K,SA124Q,LA126R,VA127I,GA210D,EA212A,TA216S,TA234E,IA237T,GA238A,EA239D,KA240G,VA241I,EA243H

VAADIGTGLADALTAPLDHKDKGLKSLTLEDSIRQNGTLTLSAQGAEKTFKAGDKDNSLNTGKLKNDKISRFDFVQKIEVDGQTITLASGEFQIYKQNHSAVVALQIEEIQDSEHSGSLVNKRQFRISGLGGEHTAFNQLPGGKAEYHGKAFSSDDPNGRLHYSIDFTKKQGYGRIEHLKTLEQNVELAAAELKADEKSHAVILGDTRYDSAEKGSYHLALFGDRAQEIAGSAEVKTADGIHHIGIAGKQ

>Mutant_number:m0070 Master.m0002: fH_V3_P6h2y_WT Mutations: PA34R,KA109E,NA111Q,NA112D,PA113S,DA114E,KA115H,TA116S,DA117G,SA118K,LA119A,SA124Q,LA126R,VA127I,GA210D,EA212A,TA216S,TA234E,IA237T,GA238A,EA239D,KA240G,VA241I,EA243H

VAADIGTGLADALTAPLDHKDKGLKSLTLEDSIRQNGTLTLSAQGAEKTFKAGDKDNSLNTGKLKNDKISRFDFVQKIEVDGQTITLASGEFQIYKQNHSAVVALQIEEIQDSEHSGKAINQRQFRISGLGGEHTAFNQLPGGKAEYHGKAFSSDDPNGRLHYSIDFTKKQGYGRIEHLKTLEQNVELAAAELKADEKSHAVILGDTRYDSAEKGSYHLALFGDRAQEIAGSAEVKTADGIHHIGIAGKQ

>Mutant_number:m0071 Master.m0002: fH_V3_P6h2y_WT Mutations: PA34R,KA109E,NA111Q,NA112D,PA113S,DA114E,KA115H,TA116S,DA117G,SA118K,LA119A,IA120V,QA122K,VA127I,GA210D,EA212A,TA216S,TA234E,IA237T,GA238A,EA239D,KA240G,VA241I,EA243H

VAADIGTGLADALTAPLDHKDKGLKSLTLEDSIRQNGTLTLSAQGAEKTFKAGDKDNSLNTGKLKNDKISRFDFVQKIEVDGQTITLASGEFQIYKQNHSAVVALQIEEIQDSEHSGKAVNKRSFLISGLGGEHTAFNQLPGGKAEYHGKAFSSDDPNGRLHYSIDFTKKQGYGRIEHLKTLEQNVELAAAELKADEKSHAVILGDTRYDSAEKGSYHLALFGDRAQEIAGSAEVKTADGIHHIGIAGKQ

>Mutant_number:m0072 Master.m0002: fH_V3_P6h2y_WT Mutations: PA34R,KA109E,NA111Q,NA112D,PA113S,DA114E,KA115H,TA116S,DA117G,SA118K,LA119A,IA120V,QA122K,SA124Q,LA126R,GA210D,EA212A,TA216S,TA234E,IA237T,GA238A,EA239D,KA240G,VA241I,EA243H

VAADIGTGLADALTAPLDHKDKGLKSLTLEDSIRQNGTLTLSAQGAEKTFKAGDKDNSLNTGKLKNDKISRFDFVQKIEVDGQTITLASGEFQIYKQNHSAVVALQIEEIQDSEHSGKAVNKRQFRVSGLGGEHTAFNQLPGGKAEYHGKAFSSDDPNGRLHYSIDFTKKQGYGRIEHLKTLEQNVELAAAELKADEKSHAVILGDTRYDSAEKGSYHLALFGDRAQEIAGSAEVKTADGIHHIGIAGKQ

>Mutant_number:m0073 Master.m0002: fH_V3_P6h2y_WT Mutations: PA34R,KA109E,NA111Q,NA112D,PA113S,DA114E,KA115H,TA116S,DA117G,SA118K,LA119A,IA120V,QA122K,SA124Q,LA126R,VA127I,LA182P,GA210D,EA212A,TA216S,TA234E,IA237T,GA238A,EA239D,KA240G,VA241I,EA243H

VAADIGTGLADALTAPLDHKDKGLKSLTLEDSIRQNGTLTLSAQGAEKTFKAGDKDNSLNTGKLKNDKISRFDFVQKIEVDGQTITLASGEFQIYKQNHSAVVALQIEEIQDSEHSGKAVNKRQFRISGLGGEHTAFNQLPGGKAEYHGKAFSSDDPNGRLHYSIDFTKKQGYGRIEHLKTPEQNVELAAAELKADEKSHAVILGDTRYDSAEKGSYHLALFGDRAQEIAGSAEVKTADGIHHIGIAGKQ

>Mutant_number:m0074 Master.m0002: fH_V3_P6h2y_WT Mutations: PA34R,KA109E,NA111Q,NA112D,PA113S,DA114E,KA115H,TA116S,DA117G,SA118K,LA119A,IA120V,QA122K,SA124Q,LA126R,VA127I,TA216S,TA234E,IA237T,GA238A,EA239D,KA240G,VA241I,EA243H

VAADIGTGLADALTAPLDHKDKGLKSLTLEDSIRQNGTLTLSAQGAEKTFKAGDKDNSLNTGKLKNDKISRFDFVQKIEVDGQTITLASGEFQIYKQNHSAVVALQIEEIQDSEHSGKAVNKRQFRISGLGGEHTAFNQLPGGKAEYHGKAFSSDDPNGRLHYSIDFTKKQGYGRIEHLKTLEQNVELAAAELKADEKSHAVILGDTRYGSEEKGSYHLALFGDRAQEIAGSAEVKTADGIHHIGIAGKQ

>Mutant_number:m0075 Master.m0002: fH_V3_P6h2y_WT Mutations: PA34R,KA109E,NA111Q,NA112D,PA113S,DA114E,KA115H,TA116S,DA117G,SA118K,LA119A,IA120V,QA122K,SA124Q,LA126R,VA127I,GA210D,EA212A,TA216S,GA238A,EA239D,KA240G,VA241I,EA243H

VAADIGTGLADALTAPLDHKDKGLKSLTLEDSIRQNGTLTLSAQGAEKTFKAGDKDNSLNTGKLKNDKISRFDFVQKIEVDGQTITLASGEFQIYKQNHSAVVALQIEEIQDSEHSGKAVNKRQFRISGLGGEHTAFNQLPGGKAEYHGKAFSSDDPNGRLHYSIDFTKKQGYGRIEHLKTLEQNVELAAAELKADEKSHAVILGDTRYDSAEKGSYHLALFGDRAQEIAGSATVKIADGIHHIGIAGKQ

>Mutant_number:m0076 Master.m0002: fH_V3_P6h2y_WT Mutations: PA34R,KA109E,NA111Q,NA112D,PA113S,DA114E,KA115H,TA116S,DA117G,SA118K,LA119A,IA120V,QA122K,SA124Q,LA126R,VA127I,GA210D,EA212A,TA216S,TA234E,IA237T,KA240G,VA241I,EA243H

VAADIGTGLADALTAPLDHKDKGLKSLTLEDSIRQNGTLTLSAQGAEKTFKAGDKDNSLNTGKLKNDKISRFDFVQKIEVDGQTITLASGEFQIYKQNHSAVVALQIEEIQDSEHSGKAVNKRQFRISGLGGEHTAFNQLPGGKAEYHGKAFSSDDPNGRLHYSIDFTKKQGYGRIEHLKTLEQNVELAAAELKADEKSHAVILGDTRYDSAEKGSYHLALFGDRAQEIAGSAEVKTGEGIHHIGIAGKQ

>Mutant_number:m0077 Master.m0002: fH_V3_P6h2y_WT Mutations: PA34R,KA109E,NA111Q,LA119A,IA120V,QA122K,SA124Q,LA126R,VA127I,GA210D,EA212A,TA216S,TA234E,IA237T,GA238A,EA239D,KA240G,VA241I,EA243H

VAADIGTGLADALTAPLDHKDKGLKSLTLEDSIRQNGTLTLSAQGAEKTFKAGDKDNSLNTGKLKNDKISRFDFVQKIEVDGQTITLASGEFQIYKQNHSAVVALQIEEIQNPDKTDSAVNKRQFRISGLGGEHTAFNQLPGGKAEYHGKAFSSDDPNGRLHYSIDFTKKQGYGRIEHLKTLEQNVELAAAELKADEKSHAVILGDTRYDSAEKGSYHLALFGDRAQEIAGSAEVKTADGIHHIGIAGKQ

>Mutant_number:m0078 Master.m0002: fH_V3_P6h2y_WT Mutations: PA34S,QA76R,TA84L,AA88E,LA105F,KA109Q,NA111Q,NA112D,PA113S,DA114E,KA115H,TA116S,DA117G,SA118K,LA119M,IA120V,NA121A,QA122K,SA124Q,LA126R,VA127I,LA204S,DA206S,TA207V,GA210N,SA211Q,EA212A,TA216S,HA218S,TA234E,IA237T,GA238V,EA239N,KA240G,VA241I,HA242R,EA243H

VAADIGTGLADALTAPLDHKDKGLKSLTLEDSISQNGTLTLSAQGAEKTFKAGDKDNSLNTGKLKNDKISRFDFVRKIEVDGQLITLESGEFQIYKQNHSAVVAFQIEQIQDSEHSGKMVAKRQFRISGLGGEHTAFNQLPGGKAEYHGKAFSSDDPNGRLHYSIDFTKKQGYGRIEHLKTLEQNVELAAAELKADEKSHAVISGSVRYNQAEKGSYSLALFGDRAQEIAGSAEVKTVNGIRHIGIAGKQ

>Mutant_number:m0079 Master.m0002: fH_V3_P6h2y_WT Mutations: DA31Q,PA34R,GA37E,AA52V,QA76R,TA84L,AA88E,LA105F,KA109Q,NA111Q,NA112D,PA113S,DA114E,KA115H,TA116S,DA117G,SA118K,LA119M,IA120V,NA121A,QA122K,SA124Q,LA126R,VA127I,LA204S,DA206S,TA207V,GA210N,SA211Q,EA212A,TA216S,HA218S,TA234E,IA237T,GA238V,EA239N,KA240G,VA241I,HA242R,EA243H

VAADIGTGLADALTAPLDHKDKGLKSLTLEQSIRQNETLTLSAQGAEKTFKVGDKDNSLNTGKLKNDKISRFDFVRKIEVDGQLITLESGEFQIYKQNHSAVVAFQIEQIQDSEHSGKMVAKRQFRISGLGGEHTAFNQLPGGKAEYHGKAFSSDDPNGRLHYSIDFTKKQGYGRIEHLKTLEQNVELAAAELKADEKSHAVISGSVRYNQAEKGSYSLALFGDRAQEIAGSAEVKTVNGIRHIGIAGKQ

>Mutant_number:m0080 Master.m0002: fH_V3_P6h2y_WT Mutations: DA31Q,PA34R,GA37E,TA84L,AA88E,LA105F,KA109Q,NA111Q,NA112D,PA113S,DA114E,KA115H,TA116S,DA117G,SA118K,LA119M,IA120V,NA121A,QA122K,SA124Q,LA126R,VA127I,LA204S,DA206S,TA207V,GA210N,SA211Q,EA212A,TA216S,HA218S,TA234E,IA237T,GA238V,EA239N,KA240G,VA241I,HA242R,EA243H

VAADIGTGLADALTAPLDHKDKGLKSLTLEQSIRQNETLTLSAQGAEKTFKAGDKDNSLNTGKLKNDKISRFDFVQKIEVDGQLITLESGEFQIYKQNHSAVVAFQIEQIQDSEHSGKMVAKRQFRISGLGGEHTAFNQLPGGKAEYHGKAFSSDDPNGRLHYSIDFTKKQGYGRIEHLKTLEQNVELAAAELKADEKSHAVISGSVRYNQAEKGSYSLALFGDRAQEIAGSAEVKTVNGIRHIGIAGKQ

>Mutant_number:m0081 Master.m0002: fH_V3_P6h2y_WT Mutations: DA31Q,PA34R,GA37E,QA76R,LA105F,KA109Q,NA111Q,NA112D,PA113S,DA114E,KA115H,TA116S,DA117G,SA118K,LA119M,IA120V,NA121A,QA122K,SA124Q,LA126R,VA127I,LA204S,DA206S,TA207V,GA210N,SA211Q,EA212A,TA216S,HA218S,TA234E,IA237T,GA238V,EA239N,KA240G,VA241I,HA242R,EA243H

VAADIGTGLADALTAPLDHKDKGLKSLTLEQSIRQNETLTLSAQGAEKTFKAGDKDNSLNTGKLKNDKISRFDFVRKIEVDGQTITLASGEFQIYKQNHSAVVAFQIEQIQDSEHSGKMVAKRQFRISGLGGEHTAFNQLPGGKAEYHGKAFSSDDPNGRLHYSIDFTKKQGYGRIEHLKTLEQNVELAAAELKADEKSHAVISGSVRYNQAEKGSYSLALFGDRAQEIAGSAEVKTVNGIRHIGIAGKQ

>Mutant_number:m0082 Master.m0002: fH_V3_P6h2y_WT Mutations: DA31Q,PA34R,GA37E,QA76R,TA84L,AA88E,LA105F,KA109Q,PA113S,DA114E,KA115H,TA116S,DA117G,SA118K,LA119M,IA120V,NA121A,QA122K,SA124Q,LA126R,VA127I,LA204S,DA206S,TA207V,GA210N,SA211Q,EA212A,TA216S,HA218S,TA234E,IA237T,GA238V,EA239N,KA240G,VA241I,HA242R,EA243H

VAADIGTGLADALTAPLDHKDKGLKSLTLEQSIRQNETLTLSAQGAEKTFKAGDKDNSLNTGKLKNDKISRFDFVRKIEVDGQLITLESGEFQIYKQNHSAVVAFQIEQINNSEHSGKMVAKRQFRISGLGGEHTAFNQLPGGKAEYHGKAFSSDDPNGRLHYSIDFTKKQGYGRIEHLKTLEQNVELAAAELKADEKSHAVISGSVRYNQAEKGSYSLALFGDRAQEIAGSAEVKTVNGIRHIGIAGKQ

>Mutant_number:m0083 Master.m0002: fH_V3_P6h2y_WT Mutations: DA31Q,PA34R,GA37E,QA76R,TA84L,AA88E,LA105F,KA109Q,NA111Q,NA112D,PA113S,TA116S,DA117G,SA118K,LA119M,IA120V,NA121A,QA122K,SA124Q,LA126R,VA127I,LA204S,DA206S,TA207V,GA210N,SA211Q,EA212A,TA216S,HA218S,TA234E,IA237T,GA238V,EA239N,KA240G,VA241I,HA242R,EA243H

VAADIGTGLADALTAPLDHKDKGLKSLTLEQSIRQNETLTLSAQGAEKTFKAGDKDNSLNTGKLKNDKISRFDFVRKIEVDGQLITLESGEFQIYKQNHSAVVAFQIEQIQDSDKSGKMVAKRQFRISGLGGEHTAFNQLPGGKAEYHGKAFSSDDPNGRLHYSIDFTKKQGYGRIEHLKTLEQNVELAAAELKADEKSHAVISGSVRYNQAEKGSYSLALFGDRAQEIAGSAEVKTVNGIRHIGIAGKQ

>Mutant_number:m0084 Master.m0002: fH_V3_P6h2y_WT Mutations: DA31Q,PA34R,GA37E,QA76R,TA84L,AA88E,LA105F,KA109Q,NA111Q,NA112D,PA113S,DA117G,SA118K,LA119M,IA120V,NA121A,QA122K,SA124Q,LA126R,VA127I,LA204S,DA206S,TA207V,GA210N,SA211Q,EA212A,TA216S,HA218S,TA234E,IA237T,GA238V,EA239N,KA240G,VA241I,HA242R,EA243H

VAADIGTGLADALTAPLDHKDKGLKSLTLEQSIRQNETLTLSAQGAEKTFKAGDKDNSLNTGKLKNDKISRFDFVRKIEVDGQLITLESGEFQIYKQNHSAVVAFQIEQIQDSDKTGKMVAKRQFRISGLGGEHTAFNQLPGGKAEYHGKAFSSDDPNGRLHYSIDFTKKQGYGRIEHLKTLEQNVELAAAELKADEKSHAVISGSVRYNQAEKGSYSLALFGDRAQEIAGSAEVKTVNGIRHIGIAGKQ

>Mutant_number:m0085 Master.m0002: fH_V3_P6h2y_WT Mutations: DA31Q,PA34R,GA37E,QA76R,TA84L,AA88E,LA105F,KA109Q,NA111Q,NA112D,PA113S,DA114E,KA115H,TA116S,IA120V,NA121A,QA122K,SA124Q,LA126R,VA127I,LA204S,DA206S,TA207V,GA210N,SA211Q,EA212A,TA216S,HA218S,TA234E,IA237T,GA238V,EA239N,KA240G,VA241I,HA242R,EA243H

VAADIGTGLADALTAPLDHKDKGLKSLTLEQSIRQNETLTLSAQGAEKTFKAGDKDNSLNTGKLKNDKISRFDFVRKIEVDGQLITLESGEFQIYKQNHSAVVAFQIEQIQDSEHSDSLVAKRQFRISGLGGEHTAFNQLPGGKAEYHGKAFSSDDPNGRLHYSIDFTKKQGYGRIEHLKTLEQNVELAAAELKADEKSHAVISGSVRYNQAEKGSYSLALFGDRAQEIAGSAEVKTVNGIRHIGIAGKQ

>Mutant_number:m0086 Master.m0002: fH_V3_P6h2y_WT Mutations: DA31Q,PA34R,GA37E,QA76R,TA84L,AA88E,LA105F,KA109Q,NA111Q,NA112D,PA113S,DA114E,KA115H,TA116S,DA117G,SA118K,LA119M,IA120V,LA126R,VA127I,LA204S,DA206S,TA207V,GA210N,SA211Q,EA212A,TA216S,HA218S,TA234E,IA237T,GA238V,EA239N,KA240G,VA241I,HA242R,EA243H

VAADIGTGLADALTAPLDHKDKGLKSLTLEQSIRQNETLTLSAQGAEKTFKAGDKDNSLNTGKLKNDKISRFDFVRKIEVDGQLITLESGEFQIYKQNHSAVVAFQIEQIQDSEHSGKMVNQRSFRISGLGGEHTAFNQLPGGKAEYHGKAFSSDDPNGRLHYSIDFTKKQGYGRIEHLKTLEQNVELAAAELKADEKSHAVISGSVRYNQAEKGSYSLALFGDRAQEIAGSAEVKTVNGIRHIGIAGKQ

>Mutant_number:m0087 Master.m0002: fH_V3_P6h2y_WT Mutations: DA31Q,PA34R,GA37E,QA76R,TA84L,AA88E,LA105F,KA109Q,NA111Q,NA112D,PA113S,DA114E,KA115H,TA116S,DA117G,SA118K,LA119M,IA120V,NA121A,QA122K,SA124Q,LA204S,DA206S,TA207V,GA210N,SA211Q,EA212A,TA216S,HA218S,TA234E,IA237T,GA238V,EA239N,KA240G,VA241I,HA242R,EA243H

VAADIGTGLADALTAPLDHKDKGLKSLTLEQSIRQNETLTLSAQGAEKTFKAGDKDNSLNTGKLKNDKISRFDFVRKIEVDGQLITLESGEFQIYKQNHSAVVAFQIEQIQDSEHSGKMVAKRQFLVSGLGGEHTAFNQLPGGKAEYHGKAFSSDDPNGRLHYSIDFTKKQGYGRIEHLKTLEQNVELAAAELKADEKSHAVISGSVRYNQAEKGSYSLALFGDRAQEIAGSAEVKTVNGIRHIGIAGKQ

>Mutant_number:m0088 Master.m0002: fH_V3_P6h2y_WT Mutations: DA31Q,PA34R,GA37E,QA76R,TA84L,AA88E,LA105F,KA109Q,NA111Q,NA112D,PA113S,DA114E,KA115H,TA116S,DA117G,SA118K,LA119M,IA120V,NA121A,QA122K,SA124Q,LA126R,VA127I,LA182P,LA204S,DA206S,TA207V,GA210N,SA211Q,EA212A,TA216S,HA218S,TA234E,IA237T,GA238V,EA239N,KA240G,VA241I,HA242R,EA243H

VAADIGTGLADALTAPLDHKDKGLKSLTLEQSIRQNETLTLSAQGAEKTFKAGDKDNSLNTGKLKNDKISRFDFVRKIEVDGQLITLESGEFQIYKQNHSAVVAFQIEQIQDSEHSGKMVAKRQFRISGLGGEHTAFNQLPGGKAEYHGKAFSSDDPNGRLHYSIDFTKKQGYGRIEHLKTPEQNVELAAAELKADEKSHAVISGSVRYNQAEKGSYSLALFGDRAQEIAGSAEVKTVNGIRHIGIAGKQ

>Mutant_number:m0089 Master.m0002: fH_V3_P6h2y_WT Mutations: DA31Q,PA34R,GA37E,QA76R,TA84L,AA88E,LA105F,KA109Q,NA111Q,NA112D,PA113S,DA114E,KA115H,TA116S,DA117G,SA118K,LA119M,IA120V,NA121A,QA122K,SA124Q,LA126R,VA127I,GA210N,SA211Q,EA212A,TA216S,HA218S,TA234E,IA237T,GA238V,EA239N,KA240G,VA241I,HA242R,EA243H

VAADIGTGLADALTAPLDHKDKGLKSLTLEQSIRQNETLTLSAQGAEKTFKAGDKDNSLNTGKLKNDKISRFDFVRKIEVDGQLITLESGEFQIYKQNHSAVVAFQIEQIQDSEHSGKMVAKRQFRISGLGGEHTAFNQLPGGKAEYHGKAFSSDDPNGRLHYSIDFTKKQGYGRIEHLKTLEQNVELAAAELKADEKSHAVILGDTRYNQAEKGSYSLALFGDRAQEIAGSAEVKTVNGIRHIGIAGKQ

>Mutant_number:m0090 Master.m0002: fH_V3_P6h2y_WT Mutations: DA31Q,PA34R,GA37E,QA76R,TA84L,AA88E,LA105F,KA109Q,NA111Q,NA112D,PA113S,DA114E,KA115H,TA116S,DA117G,SA118K,LA119M,IA120V,NA121A,QA122K,SA124Q,LA126R,VA127I,LA204S,DA206S,TA207V,GA210N,SA211Q,EA212A,TA216S,TA234E,IA237T,GA238V,EA239N,KA240G,VA241I,HA242R,EA243H

VAADIGTGLADALTAPLDHKDKGLKSLTLEQSIRQNETLTLSAQGAEKTFKAGDKDNSLNTGKLKNDKISRFDFVRKIEVDGQLITLESGEFQIYKQNHSAVVAFQIEQIQDSEHSGKMVAKRQFRISGLGGEHTAFNQLPGGKAEYHGKAFSSDDPNGRLHYSIDFTKKQGYGRIEHLKTLEQNVELAAAELKADEKSHAVISGSVRYNQAEKGSYHLALFGDRAQEIAGSAEVKTVNGIRHIGIAGKQ

>Mutant_number:m0091 Master.m0002: fH_V3_P6h2y_WT Mutations: DA31Q,PA34R,GA37E,QA76R,TA84L,AA88E,LA105F,KA109Q,NA111Q,NA112D,PA113S,DA114E,KA115H,TA116S,DA117G,SA118K,LA119M,IA120V,NA121A,QA122K,SA124Q,LA126R,VA127I,LA204S,DA206S,TA207V,GA210N,SA211Q,EA212A,TA216S,HA218S,TA234E,IA237T,VA241I,HA242R,EA243H

VAADIGTGLADALTAPLDHKDKGLKSLTLEQSIRQNETLTLSAQGAEKTFKAGDKDNSLNTGKLKNDKISRFDFVRKIEVDGQLITLESGEFQIYKQNHSAVVAFQIEQIQDSEHSGKMVAKRQFRISGLGGEHTAFNQLPGGKAEYHGKAFSSDDPNGRLHYSIDFTKKQGYGRIEHLKTLEQNVELAAAELKADEKSHAVISGSVRYNQAEKGSYSLALFGDRAQEIAGSAEVKTGEKIRHIGIAGKQ

>Mutant_number:m0092 Master.m0002: fH_V3_P6h2y_WT Mutations: DA31Q,PA34R,GA37E,QA76R,TA84L,AA88E,LA105F,KA109Q,NA111Q,NA112D,PA113S,DA114E,KA115H,TA116S,DA117G,SA118K,LA119M,IA120V,NA121A,QA122K,SA124Q,LA126R,VA127I,LA204S,DA206S,TA207V,GA210N,SA211Q,EA212A,TA216S,HA218S,TA234E,IA237T,GA238V,EA239N,KA240G,HA242R

VAADIGTGLADALTAPLDHKDKGLKSLTLEQSIRQNETLTLSAQGAEKTFKAGDKDNSLNTGKLKNDKISRFDFVRKIEVDGQLITLESGEFQIYKQNHSAVVAFQIEQIQDSEHSGKMVAKRQFRISGLGGEHTAFNQLPGGKAEYHGKAFSSDDPNGRLHYSIDFTKKQGYGRIEHLKTLEQNVELAAAELKADEKSHAVISGSVRYNQAEKGSYSLALFGDRAQEIAGSAEVKTVNGVREIGIAGKQ

>Mutant_number:m0093 Master.m0002: fH_V3_P6h2y_WT Mutations: PA34R,GA37E,QA76R,TA84L,LA105F,NA111Q,NA112D,PA113S,DA114E,KA115H,TA116S,DA117G,SA118K,IA120V,LA126R,VA127I,TA207V,TA216S,HA218S,TA234E,IA237T,KA240G,VA241I,HA242R,EA243H

VAADIGTGLADALTAPLDHKDKGLKSLTLEDSIRQNETLTLSAQGAEKTFKAGDKDNSLNTGKLKNDKISRFDFVRKIEVDGQLITLASGEFQIYKQNHSAVVAFQIEKIQDSEHSGKLVNQRSFRISGLGGEHTAFNQLPGGKAEYHGKAFSSDDPNGRLHYSIDFTKKQGYGRIEHLKTLEQNVELAAAELKADEKSHAVILGDVRYGSEEKGSYSLALFGDRAQEIAGSAEVKTGEGIRHIGIAGKQ

>Mutant_number:m0094 Master.m0002: fH_V3_P6h2y_WT Mutations: DA31Q,PA34S,GA37E,QA76R,TA84L,LA105F,NA111Q,NA112D,PA113S,DA114E,KA115H,TA116S,DA117G,SA118K,IA120V,LA126R,VA127I,TA207V,TA216S,HA218S,TA234E,IA237T,KA240G,VA241I,HA242R,EA243H

VAADIGTGLADALTAPLDHKDKGLKSLTLEQSISQNETLTLSAQGAEKTFKAGDKDNSLNTGKLKNDKISRFDFVRKIEVDGQLITLASGEFQIYKQNHSAVVAFQIEKIQDSEHSGKLVNQRSFRISGLGGEHTAFNQLPGGKAEYHGKAFSSDDPNGRLHYSIDFTKKQGYGRIEHLKTLEQNVELAAAELKADEKSHAVILGDVRYGSEEKGSYSLALFGDRAQEIAGSAEVKTGEGIRHIGIAGKQ

>Mutant_number:m0095 Master.m0002: fH_V3_P6h2y_WT Mutations: DA31Q,PA34R,QA76R,TA84L,LA105F,NA111Q,NA112D,PA113S,DA114E,KA115H,TA116S,DA117G,SA118K,IA120V,LA126R,VA127I,TA207V,TA216S,HA218S,TA234E,IA237T,KA240G,VA241I,HA242R,EA243H

VAADIGTGLADALTAPLDHKDKGLKSLTLEQSIRQNGTLTLSAQGAEKTFKAGDKDNSLNTGKLKNDKISRFDFVRKIEVDGQLITLASGEFQIYKQNHSAVVAFQIEKIQDSEHSGKLVNQRSFRISGLGGEHTAFNQLPGGKAEYHGKAFSSDDPNGRLHYSIDFTKKQGYGRIEHLKTLEQNVELAAAELKADEKSHAVILGDVRYGSEEKGSYSLALFGDRAQEIAGSAEVKTGEGIRHIGIAGKQ

>Mutant_number:m0096 Master.m0002: fH_V3_P6h2y_WT Mutations: DA31Q,PA34R,GA37E,AA52V,QA76R,TA84L,LA105F,NA111Q,NA112D,PA113S,DA114E,KA115H,TA116S,DA117G,SA118K,IA120V,LA126R,VA127I,TA207V,TA216S,HA218S,TA234E,IA237T,KA240G,VA241I,HA242R,EA243H

VAADIGTGLADALTAPLDHKDKGLKSLTLEQSIRQNETLTLSAQGAEKTFKVGDKDNSLNTGKLKNDKISRFDFVRKIEVDGQLITLASGEFQIYKQNHSAVVAFQIEKIQDSEHSGKLVNQRSFRISGLGGEHTAFNQLPGGKAEYHGKAFSSDDPNGRLHYSIDFTKKQGYGRIEHLKTLEQNVELAAAELKADEKSHAVILGDVRYGSEEKGSYSLALFGDRAQEIAGSAEVKTGEGIRHIGIAGKQ

>Mutant_number:m0097 Master.m0002: fH_V3_P6h2y_WT Mutations: DA31Q,PA34R,GA37E,TA84L,LA105F,NA111Q,NA112D,PA113S,DA114E,KA115H,TA116S,DA117G,SA118K,IA120V,LA126R,VA127I,TA207V,TA216S,HA218S,TA234E,IA237T,KA240G,VA241I,HA242R,EA243H

VAADIGTGLADALTAPLDHKDKGLKSLTLEQSIRQNETLTLSAQGAEKTFKAGDKDNSLNTGKLKNDKISRFDFVQKIEVDGQLITLASGEFQIYKQNHSAVVAFQIEKIQDSEHSGKLVNQRSFRISGLGGEHTAFNQLPGGKAEYHGKAFSSDDPNGRLHYSIDFTKKQGYGRIEHLKTLEQNVELAAAELKADEKSHAVILGDVRYGSEEKGSYSLALFGDRAQEIAGSAEVKTGEGIRHIGIAGKQ

>Mutant_number:m0098 Master.m0002: fH_V3_P6h2y_WT Mutations: DA31Q,PA34R,GA37E,QA76R,LA105F,NA111Q,NA112D,PA113S,DA114E,KA115H,TA116S,DA117G,SA118K,IA120V,LA126R,VA127I,TA207V,TA216S,HA218S,TA234E,IA237T,KA240G,VA241I,HA242R,EA243H

VAADIGTGLADALTAPLDHKDKGLKSLTLEQSIRQNETLTLSAQGAEKTFKAGDKDNSLNTGKLKNDKISRFDFVRKIEVDGQTITLASGEFQIYKQNHSAVVAFQIEKIQDSEHSGKLVNQRSFRISGLGGEHTAFNQLPGGKAEYHGKAFSSDDPNGRLHYSIDFTKKQGYGRIEHLKTLEQNVELAAAELKADEKSHAVILGDVRYGSEEKGSYSLALFGDRAQEIAGSAEVKTGEGIRHIGIAGKQ

>Mutant_number:m0099 Master.m0002: fH_V3_P6h2y_WT Mutations: DA31Q,PA34R,GA37E,QA76R,TA84L,NA98D,LA105F,NA111Q,NA112D,PA113S,DA114E,KA115H,TA116S,DA117G,SA118K,IA120V,LA126R,VA127I,TA207V,TA216S,HA218S,TA234E,IA237T,KA240G,VA241I,HA242R,EA243H

VAADIGTGLADALTAPLDHKDKGLKSLTLEQSIRQNETLTLSAQGAEKTFKAGDKDNSLNTGKLKNDKISRFDFVRKIEVDGQLITLASGEFQIYKQDHSAVVAFQIEKIQDSEHSGKLVNQRSFRISGLGGEHTAFNQLPGGKAEYHGKAFSSDDPNGRLHYSIDFTKKQGYGRIEHLKTLEQNVELAAAELKADEKSHAVILGDVRYGSEEKGSYSLALFGDRAQEIAGSAEVKTGEGIRHIGIAGKQ

>Mutant_number:m0100 Master.m0002: fH_V3_P6h2y_WT Mutations: DA31Q,PA34R,GA37E,QA76R,TA84L,NA111Q,NA112D,PA113S,DA114E,KA115H,TA116S,DA117G,SA118K,IA120V,LA126R,VA127I,TA207V,TA216S,HA218S,TA234E,IA237T,KA240G,VA241I,HA242R,EA243H

VAADIGTGLADALTAPLDHKDKGLKSLTLEQSIRQNETLTLSAQGAEKTFKAGDKDNSLNTGKLKNDKISRFDFVRKIEVDGQLITLASGEFQIYKQNHSAVVALQIEKIQDSEHSGKLVNQRSFRISGLGGEHTAFNQLPGGKAEYHGKAFSSDDPNGRLHYSIDFTKKQGYGRIEHLKTLEQNVELAAAELKADEKSHAVILGDVRYGSEEKGSYSLALFGDRAQEIAGSAEVKTGEGIRHIGIAGKQ

>Mutant_number:m0101 Master.m0002: fH_V3_P6h2y_WT Mutations: DA31Q,PA34R,GA37E,QA76R,TA84L,LA105F,NA112D,PA113S,DA114E,KA115H,TA116S,DA117G,SA118K,IA120V,LA126R,VA127I,TA207V,TA216S,HA218S,TA234E,IA237T,KA240G,VA241I,HA242R,EA243H

VAADIGTGLADALTAPLDHKDKGLKSLTLEQSIRQNETLTLSAQGAEKTFKAGDKDNSLNTGKLKNDKISRFDFVRKIEVDGQLITLASGEFQIYKQNHSAVVAFQIEKINDSEHSGKLVNQRSFRISGLGGEHTAFNQLPGGKAEYHGKAFSSDDPNGRLHYSIDFTKKQGYGRIEHLKTLEQNVELAAAELKADEKSHAVILGDVRYGSEEKGSYSLALFGDRAQEIAGSAEVKTGEGIRHIGIAGKQ

>Mutant_number:m0102 Master.m0002: fH_V3_P6h2y_WT Mutations: DA31Q,PA34R,GA37E,QA76R,TA84L,LA105F,NA111Q,KA115H,TA116S,DA117G,SA118K,IA120V,LA126R,VA127I,TA207V,TA216S,HA218S,TA234E,IA237T,KA240G,VA241I,HA242R,EA243H

VAADIGTGLADALTAPLDHKDKGLKSLTLEQSIRQNETLTLSAQGAEKTFKAGDKDNSLNTGKLKNDKISRFDFVRKIEVDGQLITLASGEFQIYKQNHSAVVAFQIEKIQNPDHSGKLVNQRSFRISGLGGEHTAFNQLPGGKAEYHGKAFSSDDPNGRLHYSIDFTKKQGYGRIEHLKTLEQNVELAAAELKADEKSHAVILGDVRYGSEEKGSYSLALFGDRAQEIAGSAEVKTGEGIRHIGIAGKQ

>Mutant_number:m0103 Master.m0002: fH_V3_P6h2y_WT Mutations: DA31Q,PA34R,GA37E,QA76R,TA84L,LA105F,NA111Q,NA112D,PA113S,DA114E,SA118K,IA120V,LA126R,VA127I,TA207V,TA216S,HA218S,TA234E,IA237T,KA240G,VA241I,HA242R,EA243H

VAADIGTGLADALTAPLDHKDKGLKSLTLEQSIRQNETLTLSAQGAEKTFKAGDKDNSLNTGKLKNDKISRFDFVRKIEVDGQLITLASGEFQIYKQNHSAVVAFQIEKIQDSEKTDKLVNQRSFRISGLGGEHTAFNQLPGGKAEYHGKAFSSDDPNGRLHYSIDFTKKQGYGRIEHLKTLEQNVELAAAELKADEKSHAVILGDVRYGSEEKGSYSLALFGDRAQEIAGSAEVKTGEGIRHIGIAGKQ

>Mutant_number:m0104 Master.m0002: fH_V3_P6h2y_WT Mutations: DA31Q,PA34R,GA37E,QA76R,TA84L,LA105F,NA111Q,NA112D,PA113S,DA114E,KA115H,TA116S,DA117G,LA126R,VA127I,TA207V,TA216S,HA218S,TA234E,IA237T,KA240G,VA241I,HA242R,EA243H

VAADIGTGLADALTAPLDHKDKGLKSLTLEQSIRQNETLTLSAQGAEKTFKAGDKDNSLNTGKLKNDKISRFDFVRKIEVDGQLITLASGEFQIYKQNHSAVVAFQIEKIQDSEHSGSLINQRSFRISGLGGEHTAFNQLPGGKAEYHGKAFSSDDPNGRLHYSIDFTKKQGYGRIEHLKTLEQNVELAAAELKADEKSHAVILGDVRYGSEEKGSYSLALFGDRAQEIAGSAEVKTGEGIRHIGIAGKQ

>Mutant_number:m0105 Master.m0002: fH_V3_P6h2y_WT Mutations: DA31Q,PA34R,GA37E,QA76R,TA84L,LA105F,NA111Q,NA112D,PA113S,DA114E,KA115H,TA116S,DA117G,SA118K,IA120V,TA207V,TA216S,HA218S,TA234E,IA237T,KA240G,VA241I,HA242R,EA243H

VAADIGTGLADALTAPLDHKDKGLKSLTLEQSIRQNETLTLSAQGAEKTFKAGDKDNSLNTGKLKNDKISRFDFVRKIEVDGQLITLASGEFQIYKQNHSAVVAFQIEKIQDSEHSGKLVNQRSFLVSGLGGEHTAFNQLPGGKAEYHGKAFSSDDPNGRLHYSIDFTKKQGYGRIEHLKTLEQNVELAAAELKADEKSHAVILGDVRYGSEEKGSYSLALFGDRAQEIAGSAEVKTGEGIRHIGIAGKQ

>Mutant_number:m0106 Master.m0002: fH_V3_P6h2y_WT Mutations: DA31Q,PA34R,GA37E,QA76R,TA84L,LA105F,NA111Q,NA112D,PA113S,DA114E,KA115H,TA116S,DA117G,SA118K,IA120V,LA126R,VA127I,TA207V,TA234E,IA237T,KA240G,VA241I,HA242R,EA243H

VAADIGTGLADALTAPLDHKDKGLKSLTLEQSIRQNETLTLSAQGAEKTFKAGDKDNSLNTGKLKNDKISRFDFVRKIEVDGQLITLASGEFQIYKQNHSAVVAFQIEKIQDSEHSGKLVNQRSFRISGLGGEHTAFNQLPGGKAEYHGKAFSSDDPNGRLHYSIDFTKKQGYGRIEHLKTLEQNVELAAAELKADEKSHAVILGDVRYGSEEKGTYHLALFGDRAQEIAGSAEVKTGEGIRHIGIAGKQ

>Mutant_number:m0107 Master.m0002: fH_V3_P6h2y_WT Mutations: DA31Q,PA34R,GA37E,QA76R,TA84L,LA105F,NA111Q,NA112D,PA113S,DA114E,KA115H,TA116S,DA117G,SA118K,IA120V,LA126R,VA127I,TA207V,TA216S,HA218S,TA234E,VA241I,HA242R,EA243H

VAADIGTGLADALTAPLDHKDKGLKSLTLEQSIRQNETLTLSAQGAEKTFKAGDKDNSLNTGKLKNDKISRFDFVRKIEVDGQLITLASGEFQIYKQNHSAVVAFQIEKIQDSEHSGKLVNQRSFRISGLGGEHTAFNQLPGGKAEYHGKAFSSDDPNGRLHYSIDFTKKQGYGRIEHLKTLEQNVELAAAELKADEKSHAVILGDVRYGSEEKGSYSLALFGDRAQEIAGSAEVKIGEKIRHIGIAGKQ

>Mutant_number:m0108 Master.m0002: fH_V3_P6h2y_WT Mutations: PA34S,GA37E,QA76R,TA84L,AA88S,LA105F,KA109E,NA111Q,NA112D,PA113S,DA114E,KA115H,TA116S,DA117G,SA118K,LA119A,IA120V,NA121D,QA122T,SA124G,LA126R,VA127I,LA204T,DA206K,TA207V,GA210D,SA211G,EA212D,TA216S,HA218S,TA234E,IA237T,GA238A,EA239D,KA240G,VA241I,HA242R,EA243H

VAADIGTGLADALTAPLDHKDKGLKSLTLEDSISQNETLTLSAQGAEKTFKAGDKDNSLNTGKLKNDKISRFDFVRKIEVDGQLITLSSGEFQIYKQNHSAVVAFQIEEIQDSEHSGKAVDTRGFRISGLGGEHTAFNQLPGGKAEYHGKAFSSDDPNGRLHYSIDFTKKQGYGRIEHLKTLEQNVELAAAELKADEKSHAVITGKVRYDGDEKGSYSLALFGDRAQEIAGSAEVKTADGIRHIGIAGKQ

>Mutant_number:m0109 Master.m0002: fH_V3_P6h2y_WT Mutations: DA31Q,PA34R,QA76R,TA84L,AA88S,LA105F,KA109E,NA111Q,NA112D,PA113S,DA114E,KA115H,TA116S,DA117G,SA118K,LA119A,IA120V,NA121D,QA122T,SA124G,LA126R,VA127I,LA204T,DA206K,TA207V,GA210D,SA211G,EA212D,TA216S,HA218S,TA234E,IA237T,GA238A,EA239D,KA240G,VA241I,HA242R,EA243H

VAADIGTGLADALTAPLDHKDKGLKSLTLEQSIRQNGTLTLSAQGAEKTFKAGDKDNSLNTGKLKNDKISRFDFVRKIEVDGQLITLSSGEFQIYKQNHSAVVAFQIEEIQDSEHSGKAVDTRGFRISGLGGEHTAFNQLPGGKAEYHGKAFSSDDPNGRLHYSIDFTKKQGYGRIEHLKTLEQNVELAAAELKADEKSHAVITGKVRYDGDEKGSYSLALFGDRAQEIAGSAEVKTADGIRHIGIAGKQ

>Mutant_number:m0110 Master.m0002: fH_V3_P6h2y_WT Mutations: DA31Q,PA34R,GA37E,AA52V,QA76R,TA84L,AA88S,LA105F,KA109E,NA111Q,NA112D,PA113S,DA114E,KA115H,TA116S,DA117G,SA118K,LA119A,IA120V,NA121D,QA122T,SA124G,LA126R,VA127I,LA204T,DA206K,TA207V,GA210D,SA211G,EA212D,TA216S,HA218S,TA234E,IA237T,GA238A,EA239D,KA240G,VA241I,HA242R,EA243H

VAADIGTGLADALTAPLDHKDKGLKSLTLEQSIRQNETLTLSAQGAEKTFKVGDKDNSLNTGKLKNDKISRFDFVRKIEVDGQLITLSSGEFQIYKQNHSAVVAFQIEEIQDSEHSGKAVDTRGFRISGLGGEHTAFNQLPGGKAEYHGKAFSSDDPNGRLHYSIDFTKKQGYGRIEHLKTLEQNVELAAAELKADEKSHAVITGKVRYDGDEKGSYSLALFGDRAQEIAGSAEVKTADGIRHIGIAGKQ

>Mutant_number:m0111 Master.m0002: fH_V3_P6h2y_WT Mutations: DA31Q,PA34R,GA37E,TA84L,AA88S,LA105F,KA109E,NA111Q,NA112D,PA113S,DA114E,KA115H,TA116S,DA117G,SA118K,LA119A,IA120V,NA121D,QA122T,SA124G,LA126R,VA127I,LA204T,DA206K,TA207V,GA210D,SA211G,EA212D,TA216S,HA218S,TA234E,IA237T,GA238A,EA239D,KA240G,VA241I,HA242R,EA243H

VAADIGTGLADALTAPLDHKDKGLKSLTLEQSIRQNETLTLSAQGAEKTFKAGDKDNSLNTGKLKNDKISRFDFVQKIEVDGQLITLSSGEFQIYKQNHSAVVAFQIEEIQDSEHSGKAVDTRGFRISGLGGEHTAFNQLPGGKAEYHGKAFSSDDPNGRLHYSIDFTKKQGYGRIEHLKTLEQNVELAAAELKADEKSHAVITGKVRYDGDEKGSYSLALFGDRAQEIAGSAEVKTADGIRHIGIAGKQ

>Mutant_number:m0112 Master.m0002: fH_V3_P6h2y_WT Mutations: DA31Q,PA34R,GA37E,QA76R,AA88S,LA105F,KA109E,NA111Q,NA112D,PA113S,DA114E,KA115H,TA116S,DA117G,SA118K,LA119A,IA120V,NA121D,QA122T,SA124G,LA126R,VA127I,LA204T,DA206K,TA207V,GA210D,SA211G,EA212D,TA216S,HA218S,TA234E,IA237T,GA238A,EA239D,KA240G,VA241I,HA242R,EA243H

VAADIGTGLADALTAPLDHKDKGLKSLTLEQSIRQNETLTLSAQGAEKTFKAGDKDNSLNTGKLKNDKISRFDFVRKIEVDGQTITLSSGEFQIYKQNHSAVVAFQIEEIQDSEHSGKAVDTRGFRISGLGGEHTAFNQLPGGKAEYHGKAFSSDDPNGRLHYSIDFTKKQGYGRIEHLKTLEQNVELAAAELKADEKSHAVITGKVRYDGDEKGSYSLALFGDRAQEIAGSAEVKTADGIRHIGIAGKQ

>Mutant_number:m0113 Master.m0002: fH_V3_P6h2y_WT Mutations: DA31Q,PA34R,GA37E,QA76R,TA84L,LA105F,KA109E,NA111Q,NA112D,PA113S,DA114E,KA115H,TA116S,DA117G,SA118K,LA119A,IA120V,NA121D,QA122T,SA124G,LA126R,VA127I,LA204T,DA206K,TA207V,GA210D,SA211G,EA212D,TA216S,HA218S,TA234E,IA237T,GA238A,EA239D,KA240G,VA241I,HA242R,EA243H

VAADIGTGLADALTAPLDHKDKGLKSLTLEQSIRQNETLTLSAQGAEKTFKAGDKDNSLNTGKLKNDKISRFDFVRKIEVDGQLITLASGEFQIYKQNHSAVVAFQIEEIQDSEHSGKAVDTRGFRISGLGGEHTAFNQLPGGKAEYHGKAFSSDDPNGRLHYSIDFTKKQGYGRIEHLKTLEQNVELAAAELKADEKSHAVITGKVRYDGDEKGSYSLALFGDRAQEIAGSAEVKTADGIRHIGIAGKQ

>Mutant_number:m0114 Master.m0002: fH_V3_P6h2y_WT Mutations: DA31Q,PA34R,GA37E,QA76R,TA84L,AA88S,NA98D,LA105F,KA109E,NA111Q,NA112D,PA113S,DA114E,KA115H,TA116S,DA117G,SA118K,LA119A,IA120V,NA121D,QA122T,SA124G,LA126R,VA127I,LA204T,DA206K,TA207V,GA210D,SA211G,EA212D,TA216S,HA218S,TA234E,IA237T,GA238A,EA239D,KA240G,VA241I,HA242R,EA243H

VAADIGTGLADALTAPLDHKDKGLKSLTLEQSIRQNETLTLSAQGAEKTFKAGDKDNSLNTGKLKNDKISRFDFVRKIEVDGQLITLSSGEFQIYKQDHSAVVAFQIEEIQDSEHSGKAVDTRGFRISGLGGEHTAFNQLPGGKAEYHGKAFSSDDPNGRLHYSIDFTKKQGYGRIEHLKTLEQNVELAAAELKADEKSHAVITGKVRYDGDEKGSYSLALFGDRAQEIAGSAEVKTADGIRHIGIAGKQ

>Mutant_number:m0115 Master.m0002: fH_V3_P6h2y_WT Mutations: DA31Q,PA34R,GA37E,QA76R,TA84L,AA88S,NA111Q,NA112D,PA113S,DA114E,KA115H,TA116S,DA117G,SA118K,LA119A,IA120V,NA121D,QA122T,SA124G,LA126R,VA127I,LA204T,DA206K,TA207V,GA210D,SA211G,EA212D,TA216S,HA218S,TA234E,IA237T,GA238A,EA239D,KA240G,VA241I,HA242R,EA243H

VAADIGTGLADALTAPLDHKDKGLKSLTLEQSIRQNETLTLSAQGAEKTFKAGDKDNSLNTGKLKNDKISRFDFVRKIEVDGQLITLSSGEFQIYKQNHSAVVALQIEKIQDSEHSGKAVDTRGFRISGLGGEHTAFNQLPGGKAEYHGKAFSSDDPNGRLHYSIDFTKKQGYGRIEHLKTLEQNVELAAAELKADEKSHAVITGKVRYDGDEKGSYSLALFGDRAQEIAGSAEVKTADGIRHIGIAGKQ

>Mutant_number:m0116 Master.m0002: fH_V3_P6h2y_WT Mutations: DA31Q,PA34R,GA37E,QA76R,TA84L,AA88S,LA105F,KA109E,PA113S,DA114E,KA115H,TA116S,DA117G,SA118K,LA119A,IA120V,NA121D,QA122T,SA124G,LA126R,VA127I,LA204T,DA206K,TA207V,GA210D,SA211G,EA212D,TA216S,HA218S,TA234E,IA237T,GA238A,EA239D,KA240G,VA241I,HA242R,EA243H

VAADIGTGLADALTAPLDHKDKGLKSLTLEQSIRQNETLTLSAQGAEKTFKAGDKDNSLNTGKLKNDKISRFDFVRKIEVDGQLITLSSGEFQIYKQNHSAVVAFQIEEINNSEHSGKAVDTRGFRISGLGGEHTAFNQLPGGKAEYHGKAFSSDDPNGRLHYSIDFTKKQGYGRIEHLKTLEQNVELAAAELKADEKSHAVITGKVRYDGDEKGSYSLALFGDRAQEIAGSAEVKTADGIRHIGIAGKQ

>Mutant_number:m0117 Master.m0002: fH_V3_P6h2y_WT Mutations: DA31Q,PA34R,GA37E,QA76R,TA84L,AA88S,LA105F,KA109E,NA111Q,NA112D,DA117G,SA118K,LA119A,IA120V,NA121D,QA122T,SA124G,LA126R,VA127I,LA204T,DA206K,TA207V,GA210D,SA211G,EA212D,TA216S,HA218S,TA234E,IA237T,GA238A,EA239D,KA240G,VA241I,HA242R,EA243H

VAADIGTGLADALTAPLDHKDKGLKSLTLEQSIRQNETLTLSAQGAEKTFKAGDKDNSLNTGKLKNDKISRFDFVRKIEVDGQLITLSSGEFQIYKQNHSAVVAFQIEEIQDPDKTGKAVDTRGFRISGLGGEHTAFNQLPGGKAEYHGKAFSSDDPNGRLHYSIDFTKKQGYGRIEHLKTLEQNVELAAAELKADEKSHAVITGKVRYDGDEKGSYSLALFGDRAQEIAGSAEVKTADGIRHIGIAGKQ

>Mutant_number:m0118 Master.m0002: fH_V3_P6h2y_WT Mutations: DA31Q,PA34R,GA37E,QA76R,TA84L,AA88S,LA105F,KA109E,NA111Q,NA112D,PA113S,DA114E,KA115H,TA116S,IA120V,NA121D,QA122T,SA124G,LA126R,VA127I,LA204T,DA206K,TA207V,GA210D,SA211G,EA212D,TA216S,HA218S,TA234E,IA237T,GA238A,EA239D,KA240G,VA241I,HA242R,EA243H

VAADIGTGLADALTAPLDHKDKGLKSLTLEQSIRQNETLTLSAQGAEKTFKAGDKDNSLNTGKLKNDKISRFDFVRKIEVDGQLITLSSGEFQIYKQNHSAVVAFQIEEIQDSEHSDSLVDTRGFRISGLGGEHTAFNQLPGGKAEYHGKAFSSDDPNGRLHYSIDFTKKQGYGRIEHLKTLEQNVELAAAELKADEKSHAVITGKVRYDGDEKGSYSLALFGDRAQEIAGSAEVKTADGIRHIGIAGKQ

>Mutant_number:m0119 Master.m0002: fH_V3_P6h2y_WT Mutations: DA31Q,PA34R,GA37E,QA76R,TA84L,AA88S,LA105F,KA109E,NA111Q,NA112D,PA113S,DA114E,KA115H,TA116S,DA117G,SA118K,LA119A,SA124G,LA126R,VA127I,LA204T,DA206K,TA207V,GA210D,SA211G,EA212D,TA216S,HA218S,TA234E,IA237T,GA238A,EA239D,KA240G,VA241I,HA242R,EA243H

VAADIGTGLADALTAPLDHKDKGLKSLTLEQSIRQNETLTLSAQGAEKTFKAGDKDNSLNTGKLKNDKISRFDFVRKIEVDGQLITLSSGEFQIYKQNHSAVVAFQIEEIQDSEHSGKAINQRGFRISGLGGEHTAFNQLPGGKAEYHGKAFSSDDPNGRLHYSIDFTKKQGYGRIEHLKTLEQNVELAAAELKADEKSHAVITGKVRYDGDEKGSYSLALFGDRAQEIAGSAEVKTADGIRHIGIAGKQ

>Mutant_number:m0120 Master.m0002: fH_V3_P6h2y_WT Mutations: DA31Q,PA34R,GA37E,QA76R,TA84L,AA88S,LA105F,KA109E,NA111Q,NA112D,PA113S,DA114E,KA115H,TA116S,DA117G,SA118K,LA119A,IA120V,NA121D,QA122T,LA204T,DA206K,TA207V,GA210D,SA211G,EA212D,TA216S,HA218S,TA234E,IA237T,GA238A,EA239D,KA240G,VA241I,HA242R,EA243H

VAADIGTGLADALTAPLDHKDKGLKSLTLEQSIRQNETLTLSAQGAEKTFKAGDKDNSLNTGKLKNDKISRFDFVRKIEVDGQLITLSSGEFQIYKQNHSAVVAFQIEEIQDSEHSGKAVDTRSFLVSGLGGEHTAFNQLPGGKAEYHGKAFSSDDPNGRLHYSIDFTKKQGYGRIEHLKTLEQNVELAAAELKADEKSHAVITGKVRYDGDEKGSYSLALFGDRAQEIAGSAEVKTADGIRHIGIAGKQ

>Mutant_number:m0121 Master.m0002: fH_V3_P6h2y_WT Mutations: DA31Q,PA34R,GA37E,QA76R,TA84L,AA88S,LA105F,KA109E,NA111Q,NA112D,PA113S,DA114E,KA115H,TA116S,DA117G,SA118K,LA119A,IA120V,NA121D,QA122T,SA124G,LA126R,VA127I,GA210D,SA211G,EA212D,TA216S,HA218S,TA234E,IA237T,GA238A,EA239D,KA240G,VA241I,HA242R,EA243H

VAADIGTGLADALTAPLDHKDKGLKSLTLEQSIRQNETLTLSAQGAEKTFKAGDKDNSLNTGKLKNDKISRFDFVRKIEVDGQLITLSSGEFQIYKQNHSAVVAFQIEEIQDSEHSGKAVDTRGFRISGLGGEHTAFNQLPGGKAEYHGKAFSSDDPNGRLHYSIDFTKKQGYGRIEHLKTLEQNVELAAAELKADEKSHAVILGDTRYDGDEKGSYSLALFGDRAQEIAGSAEVKTADGIRHIGIAGKQ

>Mutant_number:m0122 Master.m0002: fH_V3_P6h2y_WT Mutations: DA31Q,PA34R,GA37E,QA76R,TA84L,AA88S,LA105F,KA109E,NA111Q,NA112D,PA113S,DA114E,KA115H,TA116S,DA117G,SA118K,LA119A,IA120V,NA121D,QA122T,SA124G,LA126R,VA127I,LA204T,DA206K,TA207V,GA210D,SA211G,EA212D,TA216S,HA218S,TA234E,KA240G,VA241I,HA242R,EA243H

VAADIGTGLADALTAPLDHKDKGLKSLTLEQSIRQNETLTLSAQGAEKTFKAGDKDNSLNTGKLKNDKISRFDFVRKIEVDGQLITLSSGEFQIYKQNHSAVVAFQIEEIQDSEHSGKAVDTRGFRISGLGGEHTAFNQLPGGKAEYHGKAFSSDDPNGRLHYSIDFTKKQGYGRIEHLKTLEQNVELAAAELKADEKSHAVITGKVRYDGDEKGSYSLALFGDRAQEIAGSAEVKIGEGIRHIGIAGKQ

>Mutant_number:m0123 Master.m0001: fH_V1_P2ypv_WT Mutations: EA76R,VA77S

VAADIGAGLADALTAPLDHKDKGLQSLTLDQSVRKNEKLKLAAQGAEKTYGNGDSLNTGKLKNDKVSRFDFIRQIRSDGQLITLESGEFQVYKQSHSALTAFQTEQIQDSEHSGKMVAKRQFRIGDIAGEHTSFDKLPEGGRATYRGTAFGSDDAGGKLTYTIDFAAKQGNGKIEHLKSPELNVDLAAADIKPDGKRHAVISGSVLYNQAEKGSYSLGIFGGKAQEVAGSAEVKTVNGIRHIGLAAKQ

>Mutant_number:m0124 Master.m0002: fH_V3_P6h2y_WT Mutations: PA34S,AA52V,NA98D,LA182P

VAADIGTGLADALTAPLDHKDKGLKSLTLEDSISQNGTLTLSAQGAEKTFKVGDKDNSLNTGKLKNDKISRFDFVQKIEVDGQTITLASGEFQIYKQDHSAVVALQIEKINNPDKTDSLINQRSFLVSGLGGEHTAFNQLPGGKAEYHGKAFSSDDPNGRLHYSIDFTKKQGYGRIEHLKTPEQNVELAAAELKADEKSHAVILGDTRYGSEEKGTYHLALFGDRAQEIAGSATVKIGEKVHEIGIAGKQ

>Mutant_number:m0125 Master.m0001: fH_V1_P2ypv_WT Mutations: IA72V,QA74K,EA76R,VA77S

VAADIGAGLADALTAPLDHKDKGLQSLTLDQSVRKNEKLKLAAQGAEKTYGNGDSLNTGKLKNDKVSRFDFVRKIRSDGQLITLESGEFQVYKQSHSALTAFQTEQIQDSEHSGKMVAKRQFRIGDIAGEHTSFDKLPEGGRATYRGTAFGSDDAGGKLTYTIDFAAKQGNGKIEHLKSPELNVDLAAADIKPDGKRHAVISGSVLYNQAEKGSYSLGIFGGKAQEVAGSAEVKTVNGIRHIGLAAKQ

>Mutant_number:m0126 Master.m0001: fH_V1_P2ypv_WT Mutations: EA76R,VA77S,AA155P,GA156N

VAADIGAGLADALTAPLDHKDKGLQSLTLDQSVRKNEKLKLAAQGAEKTYGNGDSLNTGKLKNDKVSRFDFIRQIRSDGQLITLESGEFQVYKQSHSALTAFQTEQIQDSEHSGKMVAKRQFRIGDIAGEHTSFDKLPEGGRATYRGTAFGSDDPNGKLTYTIDFAAKQGNGKIEHLKSPELNVDLAAADIKPDGKRHAVISGSVLYNQAEKGSYSLGIFGGKAQEVAGSAEVKTVNGIRHIGLAAKQ

>Mutant_number:m0127 Master.m0002: fH_V3_P6h2y_WT Mutations: PA34R,GA37E,NA98D,LA182P

VAADIGTGLADALTAPLDHKDKGLKSLTLEDSIRQNETLTLSAQGAEKTFKAGDKDNSLNTGKLKNDKISRFDFVQKIEVDGQTITLASGEFQIYKQDHSAVVALQIEKINNPDKTDSLINQRSFLVSGLGGEHTAFNQLPGGKAEYHGKAFSSDDPNGRLHYSIDFTKKQGYGRIEHLKTPEQNVELAAAELKADEKSHAVILGDTRYGSEEKGTYHLALFGDRAQEIAGSATVKIGEKVHEIGIAGKQ

>Mutant_number:m0128 Master.m0002: fH_V3_P6h2y_WT Mutations: PA34S,AA52V,NA98D,QA122K,SA124Q,LA126R,LA182P

VAADIGTGLADALTAPLDHKDKGLKSLTLEDSISQNGTLTLSAQGAEKTFKVGDKDNSLNTGKLKNDKISRFDFVQKIEVDGQTITLASGEFQIYKQDHSAVVALQIEKINNPDKTDSLINKRQFRVSGLGGEHTAFNQLPGGKAEYHGKAFSSDDPNGRLHYSIDFTKKQGYGRIEHLKTPEQNVELAAAELKADEKSHAVILGDTRYGSEEKGTYHLALFGDRAQEIAGSATVKIGEKVHEIGIAGKQ

>Mutant_number:m0129 Master.m0002: fH_V3_P6h2y_WT Mutations: PA34S,AA52V,NA98D,LA182P,GA210N,EA212A,TA216S

VAADIGTGLADALTAPLDHKDKGLKSLTLEDSISQNGTLTLSAQGAEKTFKVGDKDNSLNTGKLKNDKISRFDFVQKIEVDGQTITLASGEFQIYKQDHSAVVALQIEKINNPDKTDSLINQRSFLVSGLGGEHTAFNQLPGGKAEYHGKAFSSDDPNGRLHYSIDFTKKQGYGRIEHLKTPEQNVELAAAELKADEKSHAVILGDTRYNSAEKGSYHLALFGDRAQEIAGSATVKIGEKVHEIGIAGKQ

>Mutant_number:m0130 Master.m0002: fH_V3_P6h2y_WT Mutations: PA34S,AA52V,NA98D,LA182P,EA239N,KA240G,VA241I,EA243H

VAADIGTGLADALTAPLDHKDKGLKSLTLEDSISQNGTLTLSAQGAEKTFKVGDKDNSLNTGKLKNDKISRFDFVQKIEVDGQTITLASGEFQIYKQDHSAVVALQIEKINNPDKTDSLINQRSFLVSGLGGEHTAFNQLPGGKAEYHGKAFSSDDPNGRLHYSIDFTKKQGYGRIEHLKTPEQNVELAAAELKADEKSHAVILGDTRYGSEEKGTYHLALFGDRAQEIAGSATVKIGNGIHHIGIAGKQ

>Mutant_number:m0131 Master.m0002: fH_V3_P6h2y_WT Mutations: PA34R,SA124Q,LA126R,VA127I,GA210D,EA212A,TA216S,TA234E,IA237T,GA238A,EA239D,KA240G,VA241I,EA243H

VAADIGTGLADALTAPLDHKDKGLKSLTLEDSIRQNGTLTLSAQGAEKTFKAGDKDNSLNTGKLKNDKISRFDFVQKIEVDGQTITLASGEFQIYKQNHSAVVALQIEKINNPDKTDSLINQRQFRISGLGGEHTAFNQLPGGKAEYHGKAFSSDDPNGRLHYSIDFTKKQGYGRIEHLKTLEQNVELAAAELKADEKSHAVILGDTRYDSAEKGSYHLALFGDRAQEIAGSAEVKTADGIHHIGIAGKQ

# END of job
