# Supplementary material for: Design of cross-reactive antigens with machine learning and high-throughput experimental evaluation
Source: Front Bioinform. 2025 Jul 16;5:1580967. doi: 10.3389/fbinf.2025.1580967 (PMC12319226; doi:10.3389/fbinf.2025.1580967)
Supplement: Supplementary file 4 [file Table7.docx]

**Table S7: Chemical and size classification of amino acid side chains** (71).

|  | **Aliphatic** | **Aromatic** | **Acidic** | **Basic** | **Hydroxilic** | **Sulfurous** | **Amidic** |
| --- | --- | --- | --- | --- | --- | --- | --- |
| **Very Large** |  | Phe  Trp  Tyr |  |  |  |  |  |
| **Large** | Ile  Leu |  |  | Lys  Arg |  | Met |  |
| **Medium** | Val |  | Glu | His |  |  | Gln |
| **Small** | Pro |  | Asp |  | Thr | Cys | Asn |
| **Very Small** | Gly  Ala |  |  |  | Ser |  |  |
