# Supplementary material for: Design of cross-reactive antigens with machine learning and high-throughput experimental evaluation
Source: Front Bioinform. 2025 Jul 16;5:1580967. doi: 10.3389/fbinf.2025.1580967 (PMC12319226; doi:10.3389/fbinf.2025.1580967)
Supplement: Supplementary file 5 [file Image2.pdf]

Figure S2

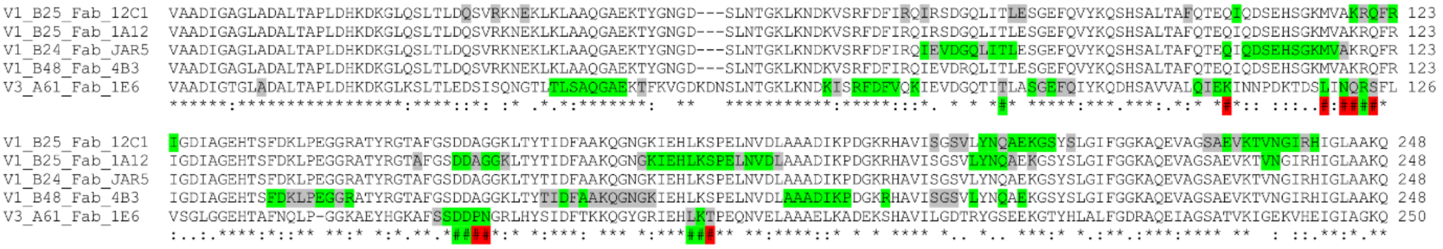

Conflicting residues, i.e. overlapping positions (#) with different residues in V1 antigens and V3 are marked in red

Fig S2: fHbp epitopes. Annotation of residues assigned to putative epitopes in models of fHbp v3.28 bound to mAb 12C1, JAR5, 1A12, 1E6, and 4B3. Residues within the 5-angstrom epitope are highlighted in green and residues within the 7-angstrom epitope are highlighted in grey.
