# Supplementary material for: Design of cross-reactive antigens with machine learning and high-throughput experimental evaluation
Source: Front Bioinform. 2025 Jul 16;5:1580967. doi: 10.3389/fbinf.2025.1580967 (PMC12319226; doi:10.3389/fbinf.2025.1580967)
Supplement: Supplementary file 8 [file Supplementaryfile2.docx]

Document S2

>v3.28

MGPDSDRLQQRRVAADIGTGLADALTAPLDHKDKGLKSLTLEDSIPQNGTLTLSAQGAEKTFKAGDKDNSLNTGKLKNDKISRFDFVQKIEVDGQTITLASGEFQIYKQNHSAVVALQIEKINNPDKTDSLINQRSFLVSGLGGEHTAFNQLPGGKAEYHGKAFSSDDPNGRLHYSIDFTKKQGYGRIEHLKTLEQNVELAAAELKADEKSHAVILGDTRYGSEEKGTYHLALFGDRAQEIAGSATVKIGEKVHEIGIAGKQLEHHHHHH

>m002415 Mutant_number:m002415 Master.m0006: m0006 Mutations: GA222N,SA223G,GA250V,GA252A

MGPDSDRLQQRRVAADIGTGLADALTAPLDHKDKGLKSLTLEDSIRQNGTLTLSAQGAEKTFKAGDKDNSLNTGKLKNDKISRFDFVQKIEVDGQTITLASGEFQIYKQNHSAVVALQIEKIQDSEHSGKLVNKRQFRISGLGGEHTAFNQLPGGKAEYHGKAFSSDDPNGRLHYSIDFTKKQGYGRIEHLKTLEQNVELAAAELKADEKSHAVILGDTRYNGAEKGSYHLALFGDRAQEIAGSAEVKTVEAIHHIGIAGKQLEHHHHHH

>m001119 Mutant_number:m001119 Master.m0006: m0006 Mutations: YA221L,GA250V,GA252S

MGPDSDRLQQRRVAADIGTGLADALTAPLDHKDKGLKSLTLEDSIRQNGTLTLSAQGAEKTFKAGDKDNSLNTGKLKNDKISRFDFVQKIEVDGQTITLASGEFQIYKQNHSAVVALQIEKIQDSEHSGKLVNKRQFRISGLGGEHTAFNQLPGGKAEYHGKAFSSDDPNGRLHYSIDFTKKQGYGRIEHLKTLEQNVELAAAELKADEKSHAVILGDTRLGSAEKGSYHLALFGDRAQEIAGSAEVKTVESIHHIGIAGKQLEHHHHHH

>m001688 Mutant_number:m001688 Master.m0006: m0006 Mutations: YA221L,GA222N,SA223G,GA250V,EA251N

MGPDSDRLQQRRVAADIGTGLADALTAPLDHKDKGLKSLTLEDSIRQNGTLTLSAQGAEKTFKAGDKDNSLNTGKLKNDKISRFDFVQKIEVDGQTITLASGEFQIYKQNHSAVVALQIEKIQDSEHSGKLVNKRQFRISGLGGEHTAFNQLPGGKAEYHGKAFSSDDPNGRLHYSIDFTKKQGYGRIEHLKTLEQNVELAAAELKADEKSHAVILGDTRLNGAEKGSYHLALFGDRAQEIAGSAEVKTVNGIHHIGIAGKQLEHHHHHH

>m002958 Mutant_number:m002958 Master.m0006: m0006 Mutations: YA221T,GA222N,SA223Q,TA249S,GA250V,EA251N,GA252A

MGPDSDRLQQRRVAADIGTGLADALTAPLDHKDKGLKSLTLEDSIRQNGTLTLSAQGAEKTFKAGDKDNSLNTGKLKNDKISRFDFVQKIEVDGQTITLASGEFQIYKQNHSAVVALQIEKIQDSEHSGKLVNKRQFRISGLGGEHTAFNQLPGGKAEYHGKAFSSDDPNGRLHYSIDFTKKQGYGRIEHLKTLEQNVELAAAELKADEKSHAVILGDTRTNQAEKGSYHLALFGDRAQEIAGSAEVKSVNAIHHIGIAGKQLEHHHHHH

>m001199 Mutant_number:m001199 Master.m0006: m0006 Mutations: YA221L,SA223G,GA250V

MGPDSDRLQQRRVAADIGTGLADALTAPLDHKDKGLKSLTLEDSIRQNGTLTLSAQGAEKTFKAGDKDNSLNTGKLKNDKISRFDFVQKIEVDGQTITLASGEFQIYKQNHSAVVALQIEKIQDSEHSGKLVNKRQFRISGLGGEHTAFNQLPGGKAEYHGKAFSSDDPNGRLHYSIDFTKKQGYGRIEHLKTLEQNVELAAAELKADEKSHAVILGDTRLGGAEKGSYHLALFGDRAQEIAGSAEVKTVEGIHHIGIAGKQLEHHHHHH

>m001375 Mutant_number:m001375 Master.m0006: m0006 Mutations: YA221L,GA222R,GA250D,EA251N,GA252A

MGPDSDRLQQRRVAADIGTGLADALTAPLDHKDKGLKSLTLEDSIRQNGTLTLSAQGAEKTFKAGDKDNSLNTGKLKNDKISRFDFVQKIEVDGQTITLASGEFQIYKQNHSAVVALQIEKIQDSEHSGKLVNKRQFRISGLGGEHTAFNQLPGGKAEYHGKAFSSDDPNGRLHYSIDFTKKQGYGRIEHLKTLEQNVELAAAELKADEKSHAVILGDTRLRSAEKGSYHLALFGDRAQEIAGSAEVKTDNAIHHIGIAGKQLEHHHHHH

>m001604 Mutant_number:m001604 Master.m0006: m0006 Mutations: YA221L,GA222N,GA250V

MGPDSDRLQQRRVAADIGTGLADALTAPLDHKDKGLKSLTLEDSIRQNGTLTLSAQGAEKTFKAGDKDNSLNTGKLKNDKISRFDFVQKIEVDGQTITLASGEFQIYKQNHSAVVALQIEKIQDSEHSGKLVNKRQFRISGLGGEHTAFNQLPGGKAEYHGKAFSSDDPNGRLHYSIDFTKKQGYGRIEHLKTLEQNVELAAAELKADEKSHAVILGDTRLNSAEKGSYHLALFGDRAQEIAGSAEVKTVEGIHHIGIAGKQLEHHHHHH

>m002416 Mutant_number:m002416 Master.m0006: m0006 Mutations: GA222N,SA223G,GA250V,EA251N

MGPDSDRLQQRRVAADIGTGLADALTAPLDHKDKGLKSLTLEDSIRQNGTLTLSAQGAEKTFKAGDKDNSLNTGKLKNDKISRFDFVQKIEVDGQTITLASGEFQIYKQNHSAVVALQIEKIQDSEHSGKLVNKRQFRISGLGGEHTAFNQLPGGKAEYHGKAFSSDDPNGRLHYSIDFTKKQGYGRIEHLKTLEQNVELAAAELKADEKSHAVILGDTRYNGAEKGSYHLALFGDRAQEIAGSAEVKTVNGIHHIGIAGKQLEHHHHHH

>m001848 Mutant_number:m001848 Master.m0006: m0006 Mutations: GA250V,GA252A

MGPDSDRLQQRRVAADIGTGLADALTAPLDHKDKGLKSLTLEDSIRQNGTLTLSAQGAEKTFKAGDKDNSLNTGKLKNDKISRFDFVQKIEVDGQTITLASGEFQIYKQNHSAVVALQIEKIQDSEHSGKLVNKRQFRISGLGGEHTAFNQLPGGKAEYHGKAFSSDDPNGRLHYSIDFTKKQGYGRIEHLKTLEQNVELAAAELKADEKSHAVILGDTRYGSAEKGSYHLALFGDRAQEIAGSAEVKTVEAIHHIGIAGKQLEHHHHHH

>m002421 Mutant_number:m002421 Master.m0006: m0006 Mutations: GA222N,SA223G,GA250V,EA251V,GA252A

MGPDSDRLQQRRVAADIGTGLADALTAPLDHKDKGLKSLTLEDSIRQNGTLTLSAQGAEKTFKAGDKDNSLNTGKLKNDKISRFDFVQKIEVDGQTITLASGEFQIYKQNHSAVVALQIEKIQDSEHSGKLVNKRQFRISGLGGEHTAFNQLPGGKAEYHGKAFSSDDPNGRLHYSIDFTKKQGYGRIEHLKTLEQNVELAAAELKADEKSHAVILGDTRYNGAEKGSYHLALFGDRAQEIAGSAEVKTVVAIHHIGIAGKQLEHHHHHH

>m002251 Mutant_number:m002251 Master.m0006: m0006 Mutations: GA222N,SA223Q,GA250V

MGPDSDRLQQRRVAADIGTGLADALTAPLDHKDKGLKSLTLEDSIRQNGTLTLSAQGAEKTFKAGDKDNSLNTGKLKNDKISRFDFVQKIEVDGQTITLASGEFQIYKQNHSAVVALQIEKIQDSEHSGKLVNKRQFRISGLGGEHTAFNQLPGGKAEYHGKAFSSDDPNGRLHYSIDFTKKQGYGRIEHLKTLEQNVELAAAELKADEKSHAVILGDTRYNQAEKGSYHLALFGDRAQEIAGSAEVKTVEGIHHIGIAGKQLEHHHHHH

>m001582 Mutant_number:m001582 Master.m0006: m0006 Mutations: YA221L,GA222N,TA249S,GA250V,EA251N,GA252A

MGPDSDRLQQRRVAADIGTGLADALTAPLDHKDKGLKSLTLEDSIRQNGTLTLSAQGAEKTFKAGDKDNSLNTGKLKNDKISRFDFVQKIEVDGQTITLASGEFQIYKQNHSAVVALQIEKIQDSEHSGKLVNKRQFRISGLGGEHTAFNQLPGGKAEYHGKAFSSDDPNGRLHYSIDFTKKQGYGRIEHLKTLEQNVELAAAELKADEKSHAVILGDTRLNSAEKGSYHLALFGDRAQEIAGSAEVKSVNAIHHIGIAGKQLEHHHHHH

>m002440 Mutant_number:m002440 Master.m0006: m0006 Mutations: GA222N,SA223G,TA249I,GA250V

MGPDSDRLQQRRVAADIGTGLADALTAPLDHKDKGLKSLTLEDSIRQNGTLTLSAQGAEKTFKAGDKDNSLNTGKLKNDKISRFDFVQKIEVDGQTITLASGEFQIYKQNHSAVVALQIEKIQDSEHSGKLVNKRQFRISGLGGEHTAFNQLPGGKAEYHGKAFSSDDPNGRLHYSIDFTKKQGYGRIEHLKTLEQNVELAAAELKADEKSHAVILGDTRYNGAEKGSYHLALFGDRAQEIAGSAEVKIVEGIHHIGIAGKQLEHHHHHH

>m002792 Mutant_number:m002792 Master.m0006: m0006 Mutations: YA221T,GA222R,TA249S,GA250V,GA252S

MGPDSDRLQQRRVAADIGTGLADALTAPLDHKDKGLKSLTLEDSIRQNGTLTLSAQGAEKTFKAGDKDNSLNTGKLKNDKISRFDFVQKIEVDGQTITLASGEFQIYKQNHSAVVALQIEKIQDSEHSGKLVNKRQFRISGLGGEHTAFNQLPGGKAEYHGKAFSSDDPNGRLHYSIDFTKKQGYGRIEHLKTLEQNVELAAAELKADEKSHAVILGDTRTRSAEKGSYHLALFGDRAQEIAGSAEVKSVESIHHIGIAGKQLEHHHHHH

>m001551 Mutant_number:m001551 Master.m0006: m0006 Mutations: YA221L,GA222N,SA223Q,TA249I,GA250V,GA252S

MGPDSDRLQQRRVAADIGTGLADALTAPLDHKDKGLKSLTLEDSIRQNGTLTLSAQGAEKTFKAGDKDNSLNTGKLKNDKISRFDFVQKIEVDGQTITLASGEFQIYKQNHSAVVALQIEKIQDSEHSGKLVNKRQFRISGLGGEHTAFNQLPGGKAEYHGKAFSSDDPNGRLHYSIDFTKKQGYGRIEHLKTLEQNVELAAAELKADEKSHAVILGDTRLNQAEKGSYHLALFGDRAQEIAGSAEVKIVESIHHIGIAGKQLEHHHHHH

>m002413 Mutant_number:m002413 Master.m0006: m0006 Mutations: GA222N,SA223G,GA250V

MGPDSDRLQQRRVAADIGTGLADALTAPLDHKDKGLKSLTLEDSIRQNGTLTLSAQGAEKTFKAGDKDNSLNTGKLKNDKISRFDFVQKIEVDGQTITLASGEFQIYKQNHSAVVALQIEKIQDSEHSGKLVNKRQFRISGLGGEHTAFNQLPGGKAEYHGKAFSSDDPNGRLHYSIDFTKKQGYGRIEHLKTLEQNVELAAAELKADEKSHAVILGDTRYNGAEKGSYHLALFGDRAQEIAGSAEVKTVEGIHHIGIAGKQLEHHHHHH

>m001039 Mutant_number:m001039 Master.m0006: m0006 Mutations: YA221L,SA223Q,GA250V,GA252A

MGPDSDRLQQRRVAADIGTGLADALTAPLDHKDKGLKSLTLEDSIRQNGTLTLSAQGAEKTFKAGDKDNSLNTGKLKNDKISRFDFVQKIEVDGQTITLASGEFQIYKQNHSAVVALQIEKIQDSEHSGKLVNKRQFRISGLGGEHTAFNQLPGGKAEYHGKAFSSDDPNGRLHYSIDFTKKQGYGRIEHLKTLEQNVELAAAELKADEKSHAVILGDTRLGQAEKGSYHLALFGDRAQEIAGSAEVKTVEAIHHIGIAGKQLEHHHHHH

>m001687 Mutant_number:m001687 Master.m0006: m0006 Mutations: YA221L,GA222N,SA223G,GA250V,GA252A

MGPDSDRLQQRRVAADIGTGLADALTAPLDHKDKGLKSLTLEDSIRQNGTLTLSAQGAEKTFKAGDKDNSLNTGKLKNDKISRFDFVQKIEVDGQTITLASGEFQIYKQNHSAVVALQIEKIQDSEHSGKLVNKRQFRISGLGGEHTAFNQLPGGKAEYHGKAFSSDDPNGRLHYSIDFTKKQGYGRIEHLKTLEQNVELAAAELKADEKSHAVILGDTRLNGAEKGSYHLALFGDRAQEIAGSAEVKTVEAIHHIGIAGKQLEHHHHHH

>m002340 Mutant_number:m002340 Master.m0006: m0006 Mutations: GA222N,GA250V,EA251V,GA252A

MGPDSDRLQQRRVAADIGTGLADALTAPLDHKDKGLKSLTLEDSIRQNGTLTLSAQGAEKTFKAGDKDNSLNTGKLKNDKISRFDFVQKIEVDGQTITLASGEFQIYKQNHSAVVALQIEKIQDSEHSGKLVNKRQFRISGLGGEHTAFNQLPGGKAEYHGKAFSSDDPNGRLHYSIDFTKKQGYGRIEHLKTLEQNVELAAAELKADEKSHAVILGDTRYNSAEKGSYHLALFGDRAQEIAGSAEVKTVVAIHHIGIAGKQLEHHHHHH

>m001927 Mutant_number:m001927 Master.m0006: m0006 Mutations: SA223G,GA250V

MGPDSDRLQQRRVAADIGTGLADALTAPLDHKDKGLKSLTLEDSIRQNGTLTLSAQGAEKTFKAGDKDNSLNTGKLKNDKISRFDFVQKIEVDGQTITLASGEFQIYKQNHSAVVALQIEKIQDSEHSGKLVNKRQFRISGLGGEHTAFNQLPGGKAEYHGKAFSSDDPNGRLHYSIDFTKKQGYGRIEHLKTLEQNVELAAAELKADEKSHAVILGDTRYGGAEKGSYHLALFGDRAQEIAGSAEVKTVEGIHHIGIAGKQLEHHHHHH

>m002332 Mutant_number:m002332 Master.m0006: m0006 Mutations: GA222N,GA250V

MGPDSDRLQQRRVAADIGTGLADALTAPLDHKDKGLKSLTLEDSIRQNGTLTLSAQGAEKTFKAGDKDNSLNTGKLKNDKISRFDFVQKIEVDGQTITLASGEFQIYKQNHSAVVALQIEKIQDSEHSGKLVNKRQFRISGLGGEHTAFNQLPGGKAEYHGKAFSSDDPNGRLHYSIDFTKKQGYGRIEHLKTLEQNVELAAAELKADEKSHAVILGDTRYNSAEKGSYHLALFGDRAQEIAGSAEVKTVEGIHHIGIAGKQLEHHHHHH

>m001605 Mutant_number:m001605 Master.m0006: m0006 Mutations: YA221L,GA222N,GA250V,GA252S

MGPDSDRLQQRRVAADIGTGLADALTAPLDHKDKGLKSLTLEDSIRQNGTLTLSAQGAEKTFKAGDKDNSLNTGKLKNDKISRFDFVQKIEVDGQTITLASGEFQIYKQNHSAVVALQIEKIQDSEHSGKLVNKRQFRISGLGGEHTAFNQLPGGKAEYHGKAFSSDDPNGRLHYSIDFTKKQGYGRIEHLKTLEQNVELAAAELKADEKSHAVILGDTRLNSAEKGSYHLALFGDRAQEIAGSAEVKTVESIHHIGIAGKQLEHHHHHH

>m002420 Mutant_number:m002420 Master.m0006: m0006 Mutations: GA222N,SA223G,GA250V,EA251V,GA252S

MGPDSDRLQQRRVAADIGTGLADALTAPLDHKDKGLKSLTLEDSIRQNGTLTLSAQGAEKTFKAGDKDNSLNTGKLKNDKISRFDFVQKIEVDGQTITLASGEFQIYKQNHSAVVALQIEKIQDSEHSGKLVNKRQFRISGLGGEHTAFNQLPGGKAEYHGKAFSSDDPNGRLHYSIDFTKKQGYGRIEHLKTLEQNVELAAAELKADEKSHAVILGDTRYNGAEKGSYHLALFGDRAQEIAGSAEVKTVVSIHHIGIAGKQLEHHHHHH

>m001607 Mutant_number:m001607 Master.m0006: m0006 Mutations: YA221L,GA222N,GA250V,EA251N

MGPDSDRLQQRRVAADIGTGLADALTAPLDHKDKGLKSLTLEDSIRQNGTLTLSAQGAEKTFKAGDKDNSLNTGKLKNDKISRFDFVQKIEVDGQTITLASGEFQIYKQNHSAVVALQIEKIQDSEHSGKLVNKRQFRISGLGGEHTAFNQLPGGKAEYHGKAFSSDDPNGRLHYSIDFTKKQGYGRIEHLKTLEQNVELAAAELKADEKSHAVILGDTRLNSAEKGSYHLALFGDRAQEIAGSAEVKTVNGIHHIGIAGKQLEHHHHHH

>m002334 Mutant_number:m002334 Master.m0006: m0006 Mutations: GA222N,GA250V,GA252A

MGPDSDRLQQRRVAADIGTGLADALTAPLDHKDKGLKSLTLEDSIRQNGTLTLSAQGAEKTFKAGDKDNSLNTGKLKNDKISRFDFVQKIEVDGQTITLASGEFQIYKQNHSAVVALQIEKIQDSEHSGKLVNKRQFRISGLGGEHTAFNQLPGGKAEYHGKAFSSDDPNGRLHYSIDFTKKQGYGRIEHLKTLEQNVELAAAELKADEKSHAVILGDTRYNSAEKGSYHLALFGDRAQEIAGSAEVKTVEAIHHIGIAGKQLEHHHHHH

>m002414 Mutant_number:m002414 Master.m0006: m0006 Mutations: GA222N,SA223G,GA250V,GA252S

MGPDSDRLQQRRVAADIGTGLADALTAPLDHKDKGLKSLTLEDSIRQNGTLTLSAQGAEKTFKAGDKDNSLNTGKLKNDKISRFDFVQKIEVDGQTITLASGEFQIYKQNHSAVVALQIEKIQDSEHSGKLVNKRQFRISGLGGEHTAFNQLPGGKAEYHGKAFSSDDPNGRLHYSIDFTKKQGYGRIEHLKTLEQNVELAAAELKADEKSHAVILGDTRYNGAEKGSYHLALFGDRAQEIAGSAEVKTVESIHHIGIAGKQLEHHHHHH

>m001768 Mutant_number:m001768 Master.m0006: m0006 Mutations: SA223Q,GA250V,GA252A

MGPDSDRLQQRRVAADIGTGLADALTAPLDHKDKGLKSLTLEDSIRQNGTLTLSAQGAEKTFKAGDKDNSLNTGKLKNDKISRFDFVQKIEVDGQTITLASGEFQIYKQNHSAVVALQIEKIQDSEHSGKLVNKRQFRISGLGGEHTAFNQLPGGKAEYHGKAFSSDDPNGRLHYSIDFTKKQGYGRIEHLKTLEQNVELAAAELKADEKSHAVILGDTRYGQAEKGSYHLALFGDRAQEIAGSAEVKTVEAIHHIGIAGKQLEHHHHHH

>m001211 Mutant_number:m001211 Master.m0006: m0006 Mutations: YA221L,SA223G,GA250D,EA251N

MGPDSDRLQQRRVAADIGTGLADALTAPLDHKDKGLKSLTLEDSIRQNGTLTLSAQGAEKTFKAGDKDNSLNTGKLKNDKISRFDFVQKIEVDGQTITLASGEFQIYKQNHSAVVALQIEKIQDSEHSGKLVNKRQFRISGLGGEHTAFNQLPGGKAEYHGKAFSSDDPNGRLHYSIDFTKKQGYGRIEHLKTLEQNVELAAAELKADEKSHAVILGDTRLGGAEKGSYHLALFGDRAQEIAGSAEVKTDNGIHHIGIAGKQLEHHHHHH

>m001608 Mutant_number:m001608 Master.m0006: m0006 Mutations: YA221L,GA222N,GA250V,EA251N,GA252S

MGPDSDRLQQRRVAADIGTGLADALTAPLDHKDKGLKSLTLEDSIRQNGTLTLSAQGAEKTFKAGDKDNSLNTGKLKNDKISRFDFVQKIEVDGQTITLASGEFQIYKQNHSAVVALQIEKIQDSEHSGKLVNKRQFRISGLGGEHTAFNQLPGGKAEYHGKAFSSDDPNGRLHYSIDFTKKQGYGRIEHLKTLEQNVELAAAELKADEKSHAVILGDTRLNSAEKGSYHLALFGDRAQEIAGSAEVKTVNSIHHIGIAGKQLEHHHHHH

>m001120 Mutant_number:m001120 Master.m0006: m0006 Mutations: YA221L,GA250V,GA252A

MGPDSDRLQQRRVAADIGTGLADALTAPLDHKDKGLKSLTLEDSIRQNGTLTLSAQGAEKTFKAGDKDNSLNTGKLKNDKISRFDFVQKIEVDGQTITLASGEFQIYKQNHSAVVALQIEKIQDSEHSGKLVNKRQFRISGLGGEHTAFNQLPGGKAEYHGKAFSSDDPNGRLHYSIDFTKKQGYGRIEHLKTLEQNVELAAAELKADEKSHAVILGDTRLGSAEKGSYHLALFGDRAQEIAGSAEVKTVEAIHHIGIAGKQLEHHHHHH

>m002281 Mutant_number:m002281 Master.m0006: m0006 Mutations: GA222N,SA223Q,TA249I,GA250V,EA251N

MGPDSDRLQQRRVAADIGTGLADALTAPLDHKDKGLKSLTLEDSIRQNGTLTLSAQGAEKTFKAGDKDNSLNTGKLKNDKISRFDFVQKIEVDGQTITLASGEFQIYKQNHSAVVALQIEKIQDSEHSGKLVNKRQFRISGLGGEHTAFNQLPGGKAEYHGKAFSSDDPNGRLHYSIDFTKKQGYGRIEHLKTLEQNVELAAAELKADEKSHAVILGDTRYNQAEKGSYHLALFGDRAQEIAGSAEVKIVNGIHHIGIAGKQLEHHHHHH

>m001928 Mutant_number:m001928 Master.m0006: m0006 Mutations: SA223G,GA250V,GA252S

MGPDSDRLQQRRVAADIGTGLADALTAPLDHKDKGLKSLTLEDSIRQNGTLTLSAQGAEKTFKAGDKDNSLNTGKLKNDKISRFDFVQKIEVDGQTITLASGEFQIYKQNHSAVVALQIEKIQDSEHSGKLVNKRQFRISGLGGEHTAFNQLPGGKAEYHGKAFSSDDPNGRLHYSIDFTKKQGYGRIEHLKTLEQNVELAAAELKADEKSHAVILGDTRYGGAEKGSYHLALFGDRAQEIAGSAEVKTVESIHHIGIAGKQLEHHHHHH

>m001418 Mutant_number:m001418 Master.m0006: m0006 Mutations: YA221L,GA222R,SA223G,TA249S,GA250V,EA251N

MGPDSDRLQQRRVAADIGTGLADALTAPLDHKDKGLKSLTLEDSIRQNGTLTLSAQGAEKTFKAGDKDNSLNTGKLKNDKISRFDFVQKIEVDGQTITLASGEFQIYKQNHSAVVALQIEKIQDSEHSGKLVNKRQFRISGLGGEHTAFNQLPGGKAEYHGKAFSSDDPNGRLHYSIDFTKKQGYGRIEHLKTLEQNVELAAAELKADEKSHAVILGDTRLRGAEKGSYHLALFGDRAQEIAGSAEVKSVNGIHHIGIAGKQLEHHHHHH

>m001660 Mutant_number:m001660 Master.m0006: m0006 Mutations: YA221L,GA222N,SA223G,TA249S,GA250V,GA252A

MGPDSDRLQQRRVAADIGTGLADALTAPLDHKDKGLKSLTLEDSIRQNGTLTLSAQGAEKTFKAGDKDNSLNTGKLKNDKISRFDFVQKIEVDGQTITLASGEFQIYKQNHSAVVALQIEKIQDSEHSGKLVNKRQFRISGLGGEHTAFNQLPGGKAEYHGKAFSSDDPNGRLHYSIDFTKKQGYGRIEHLKTLEQNVELAAAELKADEKSHAVILGDTRLNGAEKGSYHLALFGDRAQEIAGSAEVKSVEAIHHIGIAGKQLEHHHHHH

>m002392 Mutant_number:m002392 Master.m0006: m0006 Mutations: GA222N,SA223G,TA249S,GA250V,EA251V

MGPDSDRLQQRRVAADIGTGLADALTAPLDHKDKGLKSLTLEDSIRQNGTLTLSAQGAEKTFKAGDKDNSLNTGKLKNDKISRFDFVQKIEVDGQTITLASGEFQIYKQNHSAVVALQIEKIQDSEHSGKLVNKRQFRISGLGGEHTAFNQLPGGKAEYHGKAFSSDDPNGRLHYSIDFTKKQGYGRIEHLKTLEQNVELAAAELKADEKSHAVILGDTRYNGAEKGSYHLALFGDRAQEIAGSAEVKSVVGIHHIGIAGKQLEHHHHHH

>m001531 Mutant_number:m001531 Master.m0006: m0006 Mutations: YA221L,GA222N,SA223Q,GA250V,EA251V,GA252A

MGPDSDRLQQRRVAADIGTGLADALTAPLDHKDKGLKSLTLEDSIRQNGTLTLSAQGAEKTFKAGDKDNSLNTGKLKNDKISRFDFVQKIEVDGQTITLASGEFQIYKQNHSAVVALQIEKIQDSEHSGKLVNKRQFRISGLGGEHTAFNQLPGGKAEYHGKAFSSDDPNGRLHYSIDFTKKQGYGRIEHLKTLEQNVELAAAELKADEKSHAVILGDTRLNQAEKGSYHLALFGDRAQEIAGSAEVKTVVAIHHIGIAGKQLEHHHHHH

>m002333 Mutant_number:m002333 Master.m0006: m0006 Mutations: GA222N,GA250V,GA252S

MGPDSDRLQQRRVAADIGTGLADALTAPLDHKDKGLKSLTLEDSIRQNGTLTLSAQGAEKTFKAGDKDNSLNTGKLKNDKISRFDFVQKIEVDGQTITLASGEFQIYKQNHSAVVALQIEKIQDSEHSGKLVNKRQFRISGLGGEHTAFNQLPGGKAEYHGKAFSSDDPNGRLHYSIDFTKKQGYGRIEHLKTLEQNVELAAAELKADEKSHAVILGDTRYNSAEKGSYHLALFGDRAQEIAGSAEVKTVESIHHIGIAGKQLEHHHHHH

>m001118 Mutant_number:m001118 Master.m0006: m0006 Mutations: YA221L,GA250V

MGPDSDRLQQRRVAADIGTGLADALTAPLDHKDKGLKSLTLEDSIRQNGTLTLSAQGAEKTFKAGDKDNSLNTGKLKNDKISRFDFVQKIEVDGQTITLASGEFQIYKQNHSAVVALQIEKIQDSEHSGKLVNKRQFRISGLGGEHTAFNQLPGGKAEYHGKAFSSDDPNGRLHYSIDFTKKQGYGRIEHLKTLEQNVELAAAELKADEKSHAVILGDTRLGSAEKGSYHLALFGDRAQEIAGSAEVKTVEGIHHIGIAGKQLEHHHHHH

>m002335 Mutant_number:m002335 Master.m0006: m0006 Mutations: GA222N,GA250V,EA251N

MGPDSDRLQQRRVAADIGTGLADALTAPLDHKDKGLKSLTLEDSIRQNGTLTLSAQGAEKTFKAGDKDNSLNTGKLKNDKISRFDFVQKIEVDGQTITLASGEFQIYKQNHSAVVALQIEKIQDSEHSGKLVNKRQFRISGLGGEHTAFNQLPGGKAEYHGKAFSSDDPNGRLHYSIDFTKKQGYGRIEHLKTLEQNVELAAAELKADEKSHAVILGDTRYNSAEKGSYHLALFGDRAQEIAGSAEVKTVNGIHHIGIAGKQLEHHHHHH

>m001832 Mutant_number:m001832 Master.m0006: m0006 Mutations: TA249S,GA250D,EA251N

MGPDSDRLQQRRVAADIGTGLADALTAPLDHKDKGLKSLTLEDSIRQNGTLTLSAQGAEKTFKAGDKDNSLNTGKLKNDKISRFDFVQKIEVDGQTITLASGEFQIYKQNHSAVVALQIEKIQDSEHSGKLVNKRQFRISGLGGEHTAFNQLPGGKAEYHGKAFSSDDPNGRLHYSIDFTKKQGYGRIEHLKTLEQNVELAAAELKADEKSHAVILGDTRYGSAEKGSYHLALFGDRAQEIAGSAEVKSDNGIHHIGIAGKQLEHHHHHH

>m001503 Mutant_number:m001503 Master.m0006: m0006 Mutations: YA221L,GA222N,SA223Q,TA249S,GA250V,EA251V,GA252S

MGPDSDRLQQRRVAADIGTGLADALTAPLDHKDKGLKSLTLEDSIRQNGTLTLSAQGAEKTFKAGDKDNSLNTGKLKNDKISRFDFVQKIEVDGQTITLASGEFQIYKQNHSAVVALQIEKIQDSEHSGKLVNKRQFRISGLGGEHTAFNQLPGGKAEYHGKAFSSDDPNGRLHYSIDFTKKQGYGRIEHLKTLEQNVELAAAELKADEKSHAVILGDTRLNQAEKGSYHLALFGDRAQEIAGSAEVKSVVSIHHIGIAGKQLEHHHHHH

>m001658 Mutant_number:m001658 Master.m0006: m0006 Mutations: YA221L,GA222N,SA223G,TA249S,GA250V

MGPDSDRLQQRRVAADIGTGLADALTAPLDHKDKGLKSLTLEDSIRQNGTLTLSAQGAEKTFKAGDKDNSLNTGKLKNDKISRFDFVQKIEVDGQTITLASGEFQIYKQNHSAVVALQIEKIQDSEHSGKLVNKRQFRISGLGGEHTAFNQLPGGKAEYHGKAFSSDDPNGRLHYSIDFTKKQGYGRIEHLKTLEQNVELAAAELKADEKSHAVILGDTRLNGAEKGSYHLALFGDRAQEIAGSAEVKSVEGIHHIGIAGKQLEHHHHHH

>m001847 Mutant_number:m001847 Master.m0006: m0006 Mutations: GA250V,GA252S

MGPDSDRLQQRRVAADIGTGLADALTAPLDHKDKGLKSLTLEDSIRQNGTLTLSAQGAEKTFKAGDKDNSLNTGKLKNDKISRFDFVQKIEVDGQTITLASGEFQIYKQNHSAVVALQIEKIQDSEHSGKLVNKRQFRISGLGGEHTAFNQLPGGKAEYHGKAFSSDDPNGRLHYSIDFTKKQGYGRIEHLKTLEQNVELAAAELKADEKSHAVILGDTRYGSAEKGSYHLALFGDRAQEIAGSAEVKTVESIHHIGIAGKQLEHHHHHH

>m002253 Mutant_number:m002253 Master.m0006: m0006 Mutations: GA222N,SA223Q,GA250V,GA252A

MGPDSDRLQQRRVAADIGTGLADALTAPLDHKDKGLKSLTLEDSIRQNGTLTLSAQGAEKTFKAGDKDNSLNTGKLKNDKISRFDFVQKIEVDGQTITLASGEFQIYKQNHSAVVALQIEKIQDSEHSGKLVNKRQFRISGLGGEHTAFNQLPGGKAEYHGKAFSSDDPNGRLHYSIDFTKKQGYGRIEHLKTLEQNVELAAAELKADEKSHAVILGDTRYNQAEKGSYHLALFGDRAQEIAGSAEVKTVEAIHHIGIAGKQLEHHHHHH

>m001200 Mutant_number:m001200 Master.m0006: m0006 Mutations: YA221L,SA223G,GA250V,GA252S

MGPDSDRLQQRRVAADIGTGLADALTAPLDHKDKGLKSLTLEDSIRQNGTLTLSAQGAEKTFKAGDKDNSLNTGKLKNDKISRFDFVQKIEVDGQTITLASGEFQIYKQNHSAVVALQIEKIQDSEHSGKLVNKRQFRISGLGGEHTAFNQLPGGKAEYHGKAFSSDDPNGRLHYSIDFTKKQGYGRIEHLKTLEQNVELAAAELKADEKSHAVILGDTRLGGAEKGSYHLALFGDRAQEIAGSAEVKTVESIHHIGIAGKQLEHHHHHH

>m002452 Mutant_number:m002452 Master.m0006: m0006 Mutations: GA222N,SA223G,TA249I,GA250D,EA251N

MGPDSDRLQQRRVAADIGTGLADALTAPLDHKDKGLKSLTLEDSIRQNGTLTLSAQGAEKTFKAGDKDNSLNTGKLKNDKISRFDFVQKIEVDGQTITLASGEFQIYKQNHSAVVALQIEKIQDSEHSGKLVNKRQFRISGLGGEHTAFNQLPGGKAEYHGKAFSSDDPNGRLHYSIDFTKKQGYGRIEHLKTLEQNVELAAAELKADEKSHAVILGDTRYNGAEKGSYHLALFGDRAQEIAGSAEVKIDNGIHHIGIAGKQLEHHHHHH

>m002446 Mutant_number:m002446 Master.m0006: m0006 Mutations: GA222N,SA223G,TA249I,GA250V,EA251V

MGPDSDRLQQRRVAADIGTGLADALTAPLDHKDKGLKSLTLEDSIRQNGTLTLSAQGAEKTFKAGDKDNSLNTGKLKNDKISRFDFVQKIEVDGQTITLASGEFQIYKQNHSAVVALQIEKIQDSEHSGKLVNKRQFRISGLGGEHTAFNQLPGGKAEYHGKAFSSDDPNGRLHYSIDFTKKQGYGRIEHLKTLEQNVELAAAELKADEKSHAVILGDTRYNGAEKGSYHLALFGDRAQEIAGSAEVKIVVGIHHIGIAGKQLEHHHHHH

>m001040 Mutant_number:m001040 Master.m0006: m0006 Mutations: YA221L,SA223Q,GA250V,EA251N

MGPDSDRLQQRRVAADIGTGLADALTAPLDHKDKGLKSLTLEDSIRQNGTLTLSAQGAEKTFKAGDKDNSLNTGKLKNDKISRFDFVQKIEVDGQTITLASGEFQIYKQNHSAVVALQIEKIQDSEHSGKLVNKRQFRISGLGGEHTAFNQLPGGKAEYHGKAFSSDDPNGRLHYSIDFTKKQGYGRIEHLKTLEQNVELAAAELKADEKSHAVILGDTRLGQAEKGSYHLALFGDRAQEIAGSAEVKTVNGIHHIGIAGKQLEHHHHHH

>m002252 Mutant_number:m002252 Master.m0006: m0006 Mutations: GA222N,SA223Q,GA250V,GA252S

MGPDSDRLQQRRVAADIGTGLADALTAPLDHKDKGLKSLTLEDSIRQNGTLTLSAQGAEKTFKAGDKDNSLNTGKLKNDKISRFDFVQKIEVDGQTITLASGEFQIYKQNHSAVVALQIEKIQDSEHSGKLVNKRQFRISGLGGEHTAFNQLPGGKAEYHGKAFSSDDPNGRLHYSIDFTKKQGYGRIEHLKTLEQNVELAAAELKADEKSHAVILGDTRYNQAEKGSYHLALFGDRAQEIAGSAEVKTVESIHHIGIAGKQLEHHHHHH

>m002419 Mutant_number:m002419 Master.m0006: m0006 Mutations: GA222N,SA223G,GA250V,EA251V

MGPDSDRLQQRRVAADIGTGLADALTAPLDHKDKGLKSLTLEDSIRQNGTLTLSAQGAEKTFKAGDKDNSLNTGKLKNDKISRFDFVQKIEVDGQTITLASGEFQIYKQNHSAVVALQIEKIQDSEHSGKLVNKRQFRISGLGGEHTAFNQLPGGKAEYHGKAFSSDDPNGRLHYSIDFTKKQGYGRIEHLKTLEQNVELAAAELKADEKSHAVILGDTRYNGAEKGSYHLALFGDRAQEIAGSAEVKTVVGIHHIGIAGKQLEHHHHHH

>m002232 Mutant_number:m002232 Master.m0006: m0006 Mutations: GA222N,SA223Q,TA249S,GA250V,EA251V,GA252A

MGPDSDRLQQRRVAADIGTGLADALTAPLDHKDKGLKSLTLEDSIRQNGTLTLSAQGAEKTFKAGDKDNSLNTGKLKNDKISRFDFVQKIEVDGQTITLASGEFQIYKQNHSAVVALQIEKIQDSEHSGKLVNKRQFRISGLGGEHTAFNQLPGGKAEYHGKAFSSDDPNGRLHYSIDFTKKQGYGRIEHLKTLEQNVELAAAELKADEKSHAVILGDTRYNQAEKGSYHLALFGDRAQEIAGSAEVKSVVAIHHIGIAGKQLEHHHHHH

>m001929 Mutant_number:m001929 Master.m0006: m0006 Mutations: SA223G,GA250V,GA252A

MGPDSDRLQQRRVAADIGTGLADALTAPLDHKDKGLKSLTLEDSIRQNGTLTLSAQGAEKTFKAGDKDNSLNTGKLKNDKISRFDFVQKIEVDGQTITLASGEFQIYKQNHSAVVALQIEKIQDSEHSGKLVNKRQFRISGLGGEHTAFNQLPGGKAEYHGKAFSSDDPNGRLHYSIDFTKKQGYGRIEHLKTLEQNVELAAAELKADEKSHAVILGDTRYGGAEKGSYHLALFGDRAQEIAGSAEVKTVEAIHHIGIAGKQLEHHHHHH

>m002447 Mutant_number:m002447 Master.m0006: m0006 Mutations: GA222N,SA223G,TA249I,GA250V,EA251V,GA252S

MGPDSDRLQQRRVAADIGTGLADALTAPLDHKDKGLKSLTLEDSIRQNGTLTLSAQGAEKTFKAGDKDNSLNTGKLKNDKISRFDFVQKIEVDGQTITLASGEFQIYKQNHSAVVALQIEKIQDSEHSGKLVNKRQFRISGLGGEHTAFNQLPGGKAEYHGKAFSSDDPNGRLHYSIDFTKKQGYGRIEHLKTLEQNVELAAAELKADEKSHAVILGDTRYNGAEKGSYHLALFGDRAQEIAGSAEVKIVVSIHHIGIAGKQLEHHHHHH

>m002552 Mutant_number:m002552 Master.m0006: m0006 Mutations: YA221T,TA249S,GA250V,EA251N,GA252S

MGPDSDRLQQRRVAADIGTGLADALTAPLDHKDKGLKSLTLEDSIRQNGTLTLSAQGAEKTFKAGDKDNSLNTGKLKNDKISRFDFVQKIEVDGQTITLASGEFQIYKQNHSAVVALQIEKIQDSEHSGKLVNKRQFRISGLGGEHTAFNQLPGGKAEYHGKAFSSDDPNGRLHYSIDFTKKQGYGRIEHLKTLEQNVELAAAELKADEKSHAVILGDTRTGSAEKGSYHLALFGDRAQEIAGSAEVKSVNSIHHIGIAGKQLEHHHHHH

>m001621 Mutant_number:m001621 Master.m0006: m0006 Mutations: YA221L,GA222N,GA250D,EA251V,GA252A

MGPDSDRLQQRRVAADIGTGLADALTAPLDHKDKGLKSLTLEDSIRQNGTLTLSAQGAEKTFKAGDKDNSLNTGKLKNDKISRFDFVQKIEVDGQTITLASGEFQIYKQNHSAVVALQIEKIQDSEHSGKLVNKRQFRISGLGGEHTAFNQLPGGKAEYHGKAFSSDDPNGRLHYSIDFTKKQGYGRIEHLKTLEQNVELAAAELKADEKSHAVILGDTRLNSAEKGSYHLALFGDRAQEIAGSAEVKTDVAIHHIGIAGKQLEHHHHHH

>m001525 Mutant_number:m001525 Master.m0006: m0006 Mutations: YA221L,GA222N,SA223Q,GA250V,GA252A

MGPDSDRLQQRRVAADIGTGLADALTAPLDHKDKGLKSLTLEDSIRQNGTLTLSAQGAEKTFKAGDKDNSLNTGKLKNDKISRFDFVQKIEVDGQTITLASGEFQIYKQNHSAVVALQIEKIQDSEHSGKLVNKRQFRISGLGGEHTAFNQLPGGKAEYHGKAFSSDDPNGRLHYSIDFTKKQGYGRIEHLKTLEQNVELAAAELKADEKSHAVILGDTRLNQAEKGSYHLALFGDRAQEIAGSAEVKTVEAIHHIGIAGKQLEHHHHHH

>m001932 Mutant_number:m001932 Master.m0006: m0006 Mutations: SA223G,GA250V,EA251N,GA252A

MGPDSDRLQQRRVAADIGTGLADALTAPLDHKDKGLKSLTLEDSIRQNGTLTLSAQGAEKTFKAGDKDNSLNTGKLKNDKISRFDFVQKIEVDGQTITLASGEFQIYKQNHSAVVALQIEKIQDSEHSGKLVNKRQFRISGLGGEHTAFNQLPGGKAEYHGKAFSSDDPNGRLHYSIDFTKKQGYGRIEHLKTLEQNVELAAAELKADEKSHAVILGDTRYGGAEKGSYHLALFGDRAQEIAGSAEVKTVNAIHHIGIAGKQLEHHHHHH

>m001659 Mutant_number:m001659 Master.m0006: m0006 Mutations: YA221L,GA222N,SA223G,TA249S,GA250V,GA252S

MGPDSDRLQQRRVAADIGTGLADALTAPLDHKDKGLKSLTLEDSIRQNGTLTLSAQGAEKTFKAGDKDNSLNTGKLKNDKISRFDFVQKIEVDGQTITLASGEFQIYKQNHSAVVALQIEKIQDSEHSGKLVNKRQFRISGLGGEHTAFNQLPGGKAEYHGKAFSSDDPNGRLHYSIDFTKKQGYGRIEHLKTLEQNVELAAAELKADEKSHAVILGDTRLNGAEKGSYHLALFGDRAQEIAGSAEVKSVESIHHIGIAGKQLEHHHHHH

>m001015 Mutant_number:m001015 Master.m0006: m0006 Mutations: YA221L,SA223Q,TA249S,GA250V,EA251N,GA252A

MGPDSDRLQQRRVAADIGTGLADALTAPLDHKDKGLKSLTLEDSIRQNGTLTLSAQGAEKTFKAGDKDNSLNTGKLKNDKISRFDFVQKIEVDGQTITLASGEFQIYKQNHSAVVALQIEKIQDSEHSGKLVNKRQFRISGLGGEHTAFNQLPGGKAEYHGKAFSSDDPNGRLHYSIDFTKKQGYGRIEHLKTLEQNVELAAAELKADEKSHAVILGDTRLGQAEKGSYHLALFGDRAQEIAGSAEVKSVNAIHHIGIAGKQLEHHHHHH

>m001529 Mutant_number:m001529 Master.m0006: m0006 Mutations: YA221L,GA222N,SA223Q,GA250V,EA251V

MGPDSDRLQQRRVAADIGTGLADALTAPLDHKDKGLKSLTLEDSIRQNGTLTLSAQGAEKTFKAGDKDNSLNTGKLKNDKISRFDFVQKIEVDGQTITLASGEFQIYKQNHSAVVALQIEKIQDSEHSGKLVNKRQFRISGLGGEHTAFNQLPGGKAEYHGKAFSSDDPNGRLHYSIDFTKKQGYGRIEHLKTLEQNVELAAAELKADEKSHAVILGDTRLNQAEKGSYHLALFGDRAQEIAGSAEVKTVVGIHHIGIAGKQLEHHHHHH

>m003144 Mutant_number:m003144 Master.m0006: m0006 Mutations: YA221T,GA222N,SA223G,GA250V,GA252A

MGPDSDRLQQRRVAADIGTGLADALTAPLDHKDKGLKSLTLEDSIRQNGTLTLSAQGAEKTFKAGDKDNSLNTGKLKNDKISRFDFVQKIEVDGQTITLASGEFQIYKQNHSAVVALQIEKIQDSEHSGKLVNKRQFRISGLGGEHTAFNQLPGGKAEYHGKAFSSDDPNGRLHYSIDFTKKQGYGRIEHLKTLEQNVELAAAELKADEKSHAVILGDTRTNGAEKGSYHLALFGDRAQEIAGSAEVKTVEAIHHIGIAGKQLEHHHHHH

>m001636 Mutant_number:m001636 Master.m0006: m0006 Mutations: YA221L,GA222N,TA249I,GA250V,EA251N,GA252A

MGPDSDRLQQRRVAADIGTGLADALTAPLDHKDKGLKSLTLEDSIRQNGTLTLSAQGAEKTFKAGDKDNSLNTGKLKNDKISRFDFVQKIEVDGQTITLASGEFQIYKQNHSAVVALQIEKIQDSEHSGKLVNKRQFRISGLGGEHTAFNQLPGGKAEYHGKAFSSDDPNGRLHYSIDFTKKQGYGRIEHLKTLEQNVELAAAELKADEKSHAVILGDTRLNSAEKGSYHLALFGDRAQEIAGSAEVKIVNAIHHIGIAGKQLEHHHHHH

>m002254 Mutant_number:m002254 Master.m0006: m0006 Mutations: GA222N,SA223Q,GA250V,EA251N

MGPDSDRLQQRRVAADIGTGLADALTAPLDHKDKGLKSLTLEDSIRQNGTLTLSAQGAEKTFKAGDKDNSLNTGKLKNDKISRFDFVQKIEVDGQTITLASGEFQIYKQNHSAVVALQIEKIQDSEHSGKLVNKRQFRISGLGGEHTAFNQLPGGKAEYHGKAFSSDDPNGRLHYSIDFTKKQGYGRIEHLKTLEQNVELAAAELKADEKSHAVILGDTRYNQAEKGSYHLALFGDRAQEIAGSAEVKTVNGIHHIGIAGKQLEHHHHHH

>m001690 Mutant_number:m001690 Master.m0006: m0006 Mutations: YA221L,GA222N,SA223G,GA250V,EA251N,GA252A

MGPDSDRLQQRRVAADIGTGLADALTAPLDHKDKGLKSLTLEDSIRQNGTLTLSAQGAEKTFKAGDKDNSLNTGKLKNDKISRFDFVQKIEVDGQTITLASGEFQIYKQNHSAVVALQIEKIQDSEHSGKLVNKRQFRISGLGGEHTAFNQLPGGKAEYHGKAFSSDDPNGRLHYSIDFTKKQGYGRIEHLKTLEQNVELAAAELKADEKSHAVILGDTRLNGAEKGSYHLALFGDRAQEIAGSAEVKTVNAIHHIGIAGKQLEHHHHHH

>m002404 Mutant_number:m002404 Master.m0006: m0006 Mutations: GA222N,SA223G

MGPDSDRLQQRRVAADIGTGLADALTAPLDHKDKGLKSLTLEDSIRQNGTLTLSAQGAEKTFKAGDKDNSLNTGKLKNDKISRFDFVQKIEVDGQTITLASGEFQIYKQNHSAVVALQIEKIQDSEHSGKLVNKRQFRISGLGGEHTAFNQLPGGKAEYHGKAFSSDDPNGRLHYSIDFTKKQGYGRIEHLKTLEQNVELAAAELKADEKSHAVILGDTRYNGAEKGSYHLALFGDRAQEIAGSAEVKTGEGIHHIGIAGKQLEHHHHHH

>m001935 Mutant_number:m001935 Master.m0006: m0006 Mutations: SA223G,GA250V,EA251V,GA252A

MGPDSDRLQQRRVAADIGTGLADALTAPLDHKDKGLKSLTLEDSIRQNGTLTLSAQGAEKTFKAGDKDNSLNTGKLKNDKISRFDFVQKIEVDGQTITLASGEFQIYKQNHSAVVALQIEKIQDSEHSGKLVNKRQFRISGLGGEHTAFNQLPGGKAEYHGKAFSSDDPNGRLHYSIDFTKKQGYGRIEHLKTLEQNVELAAAELKADEKSHAVILGDTRYGGAEKGSYHLALFGDRAQEIAGSAEVKTVVAIHHIGIAGKQLEHHHHHH

>m001099 Mutant_number:m001099 Master.m0006: m0006 Mutations: YA221L,TA249S,GA250V,EA251V,GA252A

MGPDSDRLQQRRVAADIGTGLADALTAPLDHKDKGLKSLTLEDSIRQNGTLTLSAQGAEKTFKAGDKDNSLNTGKLKNDKISRFDFVQKIEVDGQTITLASGEFQIYKQNHSAVVALQIEKIQDSEHSGKLVNKRQFRISGLGGEHTAFNQLPGGKAEYHGKAFSSDDPNGRLHYSIDFTKKQGYGRIEHLKTLEQNVELAAAELKADEKSHAVILGDTRLGSAEKGSYHLALFGDRAQEIAGSAEVKSVVAIHHIGIAGKQLEHHHHHH

>m002256 Mutant_number:m002256 Master.m0006: m0006 Mutations: GA222N,SA223Q,GA250V,EA251N,GA252A

MGPDSDRLQQRRVAADIGTGLADALTAPLDHKDKGLKSLTLEDSIRQNGTLTLSAQGAEKTFKAGDKDNSLNTGKLKNDKISRFDFVQKIEVDGQTITLASGEFQIYKQNHSAVVALQIEKIQDSEHSGKLVNKRQFRISGLGGEHTAFNQLPGGKAEYHGKAFSSDDPNGRLHYSIDFTKKQGYGRIEHLKTLEQNVELAAAELKADEKSHAVILGDTRYNQAEKGSYHLALFGDRAQEIAGSAEVKTVNAIHHIGIAGKQLEHHHHHH

>m001912 Mutant_number:m001912 Master.m0006: m0006 Mutations: SA223G,TA249S,GA250D,EA251N

MGPDSDRLQQRRVAADIGTGLADALTAPLDHKDKGLKSLTLEDSIRQNGTLTLSAQGAEKTFKAGDKDNSLNTGKLKNDKISRFDFVQKIEVDGQTITLASGEFQIYKQNHSAVVALQIEKIQDSEHSGKLVNKRQFRISGLGGEHTAFNQLPGGKAEYHGKAFSSDDPNGRLHYSIDFTKKQGYGRIEHLKTLEQNVELAAAELKADEKSHAVILGDTRYGGAEKGSYHLALFGDRAQEIAGSAEVKSDNGIHHIGIAGKQLEHHHHHH

>m001691 Mutant_number:m001691 Master.m0006: m0006 Mutations: YA221L,GA222N,SA223G,GA250V,EA251V

MGPDSDRLQQRRVAADIGTGLADALTAPLDHKDKGLKSLTLEDSIRQNGTLTLSAQGAEKTFKAGDKDNSLNTGKLKNDKISRFDFVQKIEVDGQTITLASGEFQIYKQNHSAVVALQIEKIQDSEHSGKLVNKRQFRISGLGGEHTAFNQLPGGKAEYHGKAFSSDDPNGRLHYSIDFTKKQGYGRIEHLKTLEQNVELAAAELKADEKSHAVILGDTRLNGAEKGSYHLALFGDRAQEIAGSAEVKTVVGIHHIGIAGKQLEHHHHHH

>m001496 Mutant_number:m001496 Master.m0006: m0006 Mutations: YA221L,GA222N,SA223Q,TA249S,GA250V

MGPDSDRLQQRRVAADIGTGLADALTAPLDHKDKGLKSLTLEDSIRQNGTLTLSAQGAEKTFKAGDKDNSLNTGKLKNDKISRFDFVQKIEVDGQTITLASGEFQIYKQNHSAVVALQIEKIQDSEHSGKLVNKRQFRISGLGGEHTAFNQLPGGKAEYHGKAFSSDDPNGRLHYSIDFTKKQGYGRIEHLKTLEQNVELAAAELKADEKSHAVILGDTRLNQAEKGSYHLALFGDRAQEIAGSAEVKSVEGIHHIGIAGKQLEHHHHHH

>m001767 Mutant_number:m001767 Master.m0006: m0006 Mutations: SA223Q,GA250V,GA252S

MGPDSDRLQQRRVAADIGTGLADALTAPLDHKDKGLKSLTLEDSIRQNGTLTLSAQGAEKTFKAGDKDNSLNTGKLKNDKISRFDFVQKIEVDGQTITLASGEFQIYKQNHSAVVALQIEKIQDSEHSGKLVNKRQFRISGLGGEHTAFNQLPGGKAEYHGKAFSSDDPNGRLHYSIDFTKKQGYGRIEHLKTLEQNVELAAAELKADEKSHAVILGDTRYGQAEKGSYHLALFGDRAQEIAGSAEVKTVESIHHIGIAGKQLEHHHHHH

>m001960 Mutant_number:m001960 Master.m0006: m0006 Mutations: SA223G,TA249I,GA250V,EA251V

MGPDSDRLQQRRVAADIGTGLADALTAPLDHKDKGLKSLTLEDSIRQNGTLTLSAQGAEKTFKAGDKDNSLNTGKLKNDKISRFDFVQKIEVDGQTITLASGEFQIYKQNHSAVVALQIEKIQDSEHSGKLVNKRQFRISGLGGEHTAFNQLPGGKAEYHGKAFSSDDPNGRLHYSIDFTKKQGYGRIEHLKTLEQNVELAAAELKADEKSHAVILGDTRYGGAEKGSYHLALFGDRAQEIAGSAEVKIVVGIHHIGIAGKQLEHHHHHH

>m001207 Mutant_number:m001207 Master.m0006: m0006 Mutations: YA221L,SA223G,GA250V,EA251V,GA252A

MGPDSDRLQQRRVAADIGTGLADALTAPLDHKDKGLKSLTLEDSIRQNGTLTLSAQGAEKTFKAGDKDNSLNTGKLKNDKISRFDFVQKIEVDGQTITLASGEFQIYKQNHSAVVALQIEKIQDSEHSGKLVNKRQFRISGLGGEHTAFNQLPGGKAEYHGKAFSSDDPNGRLHYSIDFTKKQGYGRIEHLKTLEQNVELAAAELKADEKSHAVILGDTRLGGAEKGSYHLALFGDRAQEIAGSAEVKTVVAIHHIGIAGKQLEHHHHHH

>m001497 Mutant_number:m001497 Master.m0006: m0006 Mutations: YA221L,GA222N,SA223Q,TA249S,GA250V,GA252S

MGPDSDRLQQRRVAADIGTGLADALTAPLDHKDKGLKSLTLEDSIRQNGTLTLSAQGAEKTFKAGDKDNSLNTGKLKNDKISRFDFVQKIEVDGQTITLASGEFQIYKQNHSAVVALQIEKIQDSEHSGKLVNKRQFRISGLGGEHTAFNQLPGGKAEYHGKAFSSDDPNGRLHYSIDFTKKQGYGRIEHLKTLEQNVELAAAELKADEKSHAVILGDTRLNQAEKGSYHLALFGDRAQEIAGSAEVKSVESIHHIGIAGKQLEHHHHHH

>m001448 Mutant_number:m001448 Master.m0006: m0006 Mutations: YA221L,GA222R,SA223G,GA250V,EA251V

MGPDSDRLQQRRVAADIGTGLADALTAPLDHKDKGLKSLTLEDSIRQNGTLTLSAQGAEKTFKAGDKDNSLNTGKLKNDKISRFDFVQKIEVDGQTITLASGEFQIYKQNHSAVVALQIEKIQDSEHSGKLVNKRQFRISGLGGEHTAFNQLPGGKAEYHGKAFSSDDPNGRLHYSIDFTKKQGYGRIEHLKTLEQNVELAAAELKADEKSHAVILGDTRLRGAEKGSYHLALFGDRAQEIAGSAEVKTVVGIHHIGIAGKQLEHHHHHH

>m001037 Mutant_number:m001037 Master.m0006: m0006 Mutations: YA221L,SA223Q,GA250V

MGPDSDRLQQRRVAADIGTGLADALTAPLDHKDKGLKSLTLEDSIRQNGTLTLSAQGAEKTFKAGDKDNSLNTGKLKNDKISRFDFVQKIEVDGQTITLASGEFQIYKQNHSAVVALQIEKIQDSEHSGKLVNKRQFRISGLGGEHTAFNQLPGGKAEYHGKAFSSDDPNGRLHYSIDFTKKQGYGRIEHLKTLEQNVELAAAELKADEKSHAVILGDTRLGQAEKGSYHLALFGDRAQEIAGSAEVKTVEGIHHIGIAGKQLEHHHHHH

>m001663 Mutant_number:m001663 Master.m0006: m0006 Mutations: YA221L,GA222N,SA223G,TA249S,GA250V,EA251N,GA252A

MGPDSDRLQQRRVAADIGTGLADALTAPLDHKDKGLKSLTLEDSIRQNGTLTLSAQGAEKTFKAGDKDNSLNTGKLKNDKISRFDFVQKIEVDGQTITLASGEFQIYKQNHSAVVALQIEKIQDSEHSGKLVNKRQFRISGLGGEHTAFNQLPGGKAEYHGKAFSSDDPNGRLHYSIDFTKKQGYGRIEHLKTLEQNVELAAAELKADEKSHAVILGDTRLNGAEKGSYHLALFGDRAQEIAGSAEVKSVNAIHHIGIAGKQLEHHHHHH

>m001556 Mutant_number:m001556 Master.m0006: m0006 Mutations: YA221L,GA222N,SA223Q,TA249I,GA250V,EA251V

MGPDSDRLQQRRVAADIGTGLADALTAPLDHKDKGLKSLTLEDSIRQNGTLTLSAQGAEKTFKAGDKDNSLNTGKLKNDKISRFDFVQKIEVDGQTITLASGEFQIYKQNHSAVVALQIEKIQDSEHSGKLVNKRQFRISGLGGEHTAFNQLPGGKAEYHGKAFSSDDPNGRLHYSIDFTKKQGYGRIEHLKTLEQNVELAAAELKADEKSHAVILGDTRLNQAEKGSYHLALFGDRAQEIAGSAEVKIVVGIHHIGIAGKQLEHHHHHH

>m001820 Mutant_number:m001820 Master.m0006: m0006 Mutations: TA249S,GA250V

MGPDSDRLQQRRVAADIGTGLADALTAPLDHKDKGLKSLTLEDSIRQNGTLTLSAQGAEKTFKAGDKDNSLNTGKLKNDKISRFDFVQKIEVDGQTITLASGEFQIYKQNHSAVVALQIEKIQDSEHSGKLVNKRQFRISGLGGEHTAFNQLPGGKAEYHGKAFSSDDPNGRLHYSIDFTKKQGYGRIEHLKTLEQNVELAAAELKADEKSHAVILGDTRYGSAEKGSYHLALFGDRAQEIAGSAEVKSVEGIHHIGIAGKQLEHHHHHH

>m003063 Mutant_number:m003063 Master.m0006: m0006 Mutations: YA221T,GA222N,GA250V,GA252A

MGPDSDRLQQRRVAADIGTGLADALTAPLDHKDKGLKSLTLEDSIRQNGTLTLSAQGAEKTFKAGDKDNSLNTGKLKNDKISRFDFVQKIEVDGQTITLASGEFQIYKQNHSAVVALQIEKIQDSEHSGKLVNKRQFRISGLGGEHTAFNQLPGGKAEYHGKAFSSDDPNGRLHYSIDFTKKQGYGRIEHLKTLEQNVELAAAELKADEKSHAVILGDTRTNSAEKGSYHLALFGDRAQEIAGSAEVKTVEAIHHIGIAGKQLEHHHHHH

>m001403 Mutant_number:m001403 Master.m0006: m0006 Mutations: YA221L,GA222R,TA249I,GA250D,EA251V

MGPDSDRLQQRRVAADIGTGLADALTAPLDHKDKGLKSLTLEDSIRQNGTLTLSAQGAEKTFKAGDKDNSLNTGKLKNDKISRFDFVQKIEVDGQTITLASGEFQIYKQNHSAVVALQIEKIQDSEHSGKLVNKRQFRISGLGGEHTAFNQLPGGKAEYHGKAFSSDDPNGRLHYSIDFTKKQGYGRIEHLKTLEQNVELAAAELKADEKSHAVILGDTRLRSAEKGSYHLALFGDRAQEIAGSAEVKIDVGIHHIGIAGKQLEHHHHHH

>m001523 Mutant_number:m001523 Master.m0006: m0006 Mutations: YA221L,GA222N,SA223Q,GA250V

MGPDSDRLQQRRVAADIGTGLADALTAPLDHKDKGLKSLTLEDSIRQNGTLTLSAQGAEKTFKAGDKDNSLNTGKLKNDKISRFDFVQKIEVDGQTITLASGEFQIYKQNHSAVVALQIEKIQDSEHSGKLVNKRQFRISGLGGEHTAFNQLPGGKAEYHGKAFSSDDPNGRLHYSIDFTKKQGYGRIEHLKTLEQNVELAAAELKADEKSHAVILGDTRLNQAEKGSYHLALFGDRAQEIAGSAEVKTVEGIHHIGIAGKQLEHHHHHH

>m002173 Mutant_number:m002173 Master.m0006: m0006 Mutations: GA222R,SA223G,GA250V,EA251N

MGPDSDRLQQRRVAADIGTGLADALTAPLDHKDKGLKSLTLEDSIRQNGTLTLSAQGAEKTFKAGDKDNSLNTGKLKNDKISRFDFVQKIEVDGQTITLASGEFQIYKQNHSAVVALQIEKIQDSEHSGKLVNKRQFRISGLGGEHTAFNQLPGGKAEYHGKAFSSDDPNGRLHYSIDFTKKQGYGRIEHLKTLEQNVELAAAELKADEKSHAVILGDTRYRGAEKGSYHLALFGDRAQEIAGSAEVKTVNGIHHIGIAGKQLEHHHHHH

>m001152 Mutant_number:m001152 Master.m0006: m0006 Mutations: YA221L,TA249I,GA250V,EA251V,GA252S

MGPDSDRLQQRRVAADIGTGLADALTAPLDHKDKGLKSLTLEDSIRQNGTLTLSAQGAEKTFKAGDKDNSLNTGKLKNDKISRFDFVQKIEVDGQTITLASGEFQIYKQNHSAVVALQIEKIQDSEHSGKLVNKRQFRISGLGGEHTAFNQLPGGKAEYHGKAFSSDDPNGRLHYSIDFTKKQGYGRIEHLKTLEQNVELAAAELKADEKSHAVILGDTRLGSAEKGSYHLALFGDRAQEIAGSAEVKIVVSIHHIGIAGKQLEHHHHHH

>m001854 Mutant_number:m001854 Master.m0006: m0006 Mutations: GA250V,EA251V,GA252A

MGPDSDRLQQRRVAADIGTGLADALTAPLDHKDKGLKSLTLEDSIRQNGTLTLSAQGAEKTFKAGDKDNSLNTGKLKNDKISRFDFVQKIEVDGQTITLASGEFQIYKQNHSAVVALQIEKIQDSEHSGKLVNKRQFRISGLGGEHTAFNQLPGGKAEYHGKAFSSDDPNGRLHYSIDFTKKQGYGRIEHLKTLEQNVELAAAELKADEKSHAVILGDTRYGSAEKGSYHLALFGDRAQEIAGSAEVKTVVAIHHIGIAGKQLEHHHHHH

>m001720 Mutant_number:m001720 Master.m0006: m0006 Mutations: YA221L,GA222N,SA223G,TA249I,GA250V,EA251V,GA252A

MGPDSDRLQQRRVAADIGTGLADALTAPLDHKDKGLKSLTLEDSIRQNGTLTLSAQGAEKTFKAGDKDNSLNTGKLKNDKISRFDFVQKIEVDGQTITLASGEFQIYKQNHSAVVALQIEKIQDSEHSGKLVNKRQFRISGLGGEHTAFNQLPGGKAEYHGKAFSSDDPNGRLHYSIDFTKKQGYGRIEHLKTLEQNVELAAAELKADEKSHAVILGDTRLNGAEKGSYHLALFGDRAQEIAGSAEVKIVVAIHHIGIAGKQLEHHHHHH

>m001336 Mutant_number:m001336 Master.m0006: m0006 Mutations: YA221L,GA222R,TA249S,GA250V,GA252A

MGPDSDRLQQRRVAADIGTGLADALTAPLDHKDKGLKSLTLEDSIRQNGTLTLSAQGAEKTFKAGDKDNSLNTGKLKNDKISRFDFVQKIEVDGQTITLASGEFQIYKQNHSAVVALQIEKIQDSEHSGKLVNKRQFRISGLGGEHTAFNQLPGGKAEYHGKAFSSDDPNGRLHYSIDFTKKQGYGRIEHLKTLEQNVELAAAELKADEKSHAVILGDTRLRSAEKGSYHLALFGDRAQEIAGSAEVKSVEAIHHIGIAGKQLEHHHHHH

>m001581 Mutant_number:m001581 Master.m0006: m0006 Mutations: YA221L,GA222N,TA249S,GA250V,EA251N,GA252S

MGPDSDRLQQRRVAADIGTGLADALTAPLDHKDKGLKSLTLEDSIRQNGTLTLSAQGAEKTFKAGDKDNSLNTGKLKNDKISRFDFVQKIEVDGQTITLASGEFQIYKQNHSAVVALQIEKIQDSEHSGKLVNKRQFRISGLGGEHTAFNQLPGGKAEYHGKAFSSDDPNGRLHYSIDFTKKQGYGRIEHLKTLEQNVELAAAELKADEKSHAVILGDTRLNSAEKGSYHLALFGDRAQEIAGSAEVKSVNSIHHIGIAGKQLEHHHHHH

>m002429 Mutant_number:m002429 Master.m0006: m0006 Mutations: GA222N,SA223G,GA250D,EA251V,GA252S

MGPDSDRLQQRRVAADIGTGLADALTAPLDHKDKGLKSLTLEDSIRQNGTLTLSAQGAEKTFKAGDKDNSLNTGKLKNDKISRFDFVQKIEVDGQTITLASGEFQIYKQNHSAVVALQIEKIQDSEHSGKLVNKRQFRISGLGGEHTAFNQLPGGKAEYHGKAFSSDDPNGRLHYSIDFTKKQGYGRIEHLKTLEQNVELAAAELKADEKSHAVILGDTRYNGAEKGSYHLALFGDRAQEIAGSAEVKTDVSIHHIGIAGKQLEHHHHHH

>m001213 Mutant_number:m001213 Master.m0006: m0006 Mutations: YA221L,SA223G,GA250D,EA251N,GA252A

MGPDSDRLQQRRVAADIGTGLADALTAPLDHKDKGLKSLTLEDSIRQNGTLTLSAQGAEKTFKAGDKDNSLNTGKLKNDKISRFDFVQKIEVDGQTITLASGEFQIYKQNHSAVVALQIEKIQDSEHSGKLVNKRQFRISGLGGEHTAFNQLPGGKAEYHGKAFSSDDPNGRLHYSIDFTKKQGYGRIEHLKTLEQNVELAAAELKADEKSHAVILGDTRLGGAEKGSYHLALFGDRAQEIAGSAEVKTDNAIHHIGIAGKQLEHHHHHH

>m001177 Mutant_number:m001177 Master.m0006: m0006 Mutations: YA221L,SA223G,TA249S,GA250V,EA251N,GA252A

MGPDSDRLQQRRVAADIGTGLADALTAPLDHKDKGLKSLTLEDSIRQNGTLTLSAQGAEKTFKAGDKDNSLNTGKLKNDKISRFDFVQKIEVDGQTITLASGEFQIYKQNHSAVVALQIEKIQDSEHSGKLVNKRQFRISGLGGEHTAFNQLPGGKAEYHGKAFSSDDPNGRLHYSIDFTKKQGYGRIEHLKTLEQNVELAAAELKADEKSHAVILGDTRLGGAEKGSYHLALFGDRAQEIAGSAEVKSVNAIHHIGIAGKQLEHHHHHH

>m001822 Mutant_number:m001822 Master.m0006: m0006 Mutations: TA249S,GA250V,GA252A

MGPDSDRLQQRRVAADIGTGLADALTAPLDHKDKGLKSLTLEDSIRQNGTLTLSAQGAEKTFKAGDKDNSLNTGKLKNDKISRFDFVQKIEVDGQTITLASGEFQIYKQNHSAVVALQIEKIQDSEHSGKLVNKRQFRISGLGGEHTAFNQLPGGKAEYHGKAFSSDDPNGRLHYSIDFTKKQGYGRIEHLKTLEQNVELAAAELKADEKSHAVILGDTRYGSAEKGSYHLALFGDRAQEIAGSAEVKSVEAIHHIGIAGKQLEHHHHHH

>m001661 Mutant_number:m001661 Master.m0006: m0006 Mutations: YA221L,GA222N,SA223G,TA249S,GA250V,EA251N

MGPDSDRLQQRRVAADIGTGLADALTAPLDHKDKGLKSLTLEDSIRQNGTLTLSAQGAEKTFKAGDKDNSLNTGKLKNDKISRFDFVQKIEVDGQTITLASGEFQIYKQNHSAVVALQIEKIQDSEHSGKLVNKRQFRISGLGGEHTAFNQLPGGKAEYHGKAFSSDDPNGRLHYSIDFTKKQGYGRIEHLKTLEQNVELAAAELKADEKSHAVILGDTRLNGAEKGSYHLALFGDRAQEIAGSAEVKSVNGIHHIGIAGKQLEHHHHHH

>m001672 Mutant_number:m001672 Master.m0006: m0006 Mutations: YA221L,GA222N,SA223G,TA249S,GA250D,EA251N,GA252A

MGPDSDRLQQRRVAADIGTGLADALTAPLDHKDKGLKSLTLEDSIRQNGTLTLSAQGAEKTFKAGDKDNSLNTGKLKNDKISRFDFVQKIEVDGQTITLASGEFQIYKQNHSAVVALQIEKIQDSEHSGKLVNKRQFRISGLGGEHTAFNQLPGGKAEYHGKAFSSDDPNGRLHYSIDFTKKQGYGRIEHLKTLEQNVELAAAELKADEKSHAVILGDTRLNGAEKGSYHLALFGDRAQEIAGSAEVKSDNAIHHIGIAGKQLEHHHHHH

>m002367 Mutant_number:m002367 Master.m0006: m0006 Mutations: GA222N,TA249I,GA250V,EA251V,GA252A

MGPDSDRLQQRRVAADIGTGLADALTAPLDHKDKGLKSLTLEDSIRQNGTLTLSAQGAEKTFKAGDKDNSLNTGKLKNDKISRFDFVQKIEVDGQTITLASGEFQIYKQNHSAVVALQIEKIQDSEHSGKLVNKRQFRISGLGGEHTAFNQLPGGKAEYHGKAFSSDDPNGRLHYSIDFTKKQGYGRIEHLKTLEQNVELAAAELKADEKSHAVILGDTRYNSAEKGSYHLALFGDRAQEIAGSAEVKIVVAIHHIGIAGKQLEHHHHHH

>m002389 Mutant_number:m002389 Master.m0006: m0006 Mutations: GA222N,SA223G,TA249S,GA250V,EA251N

MGPDSDRLQQRRVAADIGTGLADALTAPLDHKDKGLKSLTLEDSIRQNGTLTLSAQGAEKTFKAGDKDNSLNTGKLKNDKISRFDFVQKIEVDGQTITLASGEFQIYKQNHSAVVALQIEKIQDSEHSGKLVNKRQFRISGLGGEHTAFNQLPGGKAEYHGKAFSSDDPNGRLHYSIDFTKKQGYGRIEHLKTLEQNVELAAAELKADEKSHAVILGDTRYNGAEKGSYHLALFGDRAQEIAGSAEVKSVNGIHHIGIAGKQLEHHHHHH

>m002336 Mutant_number:m002336 Master.m0006: m0006 Mutations: GA222N,GA250V,EA251N,GA252S

MGPDSDRLQQRRVAADIGTGLADALTAPLDHKDKGLKSLTLEDSIRQNGTLTLSAQGAEKTFKAGDKDNSLNTGKLKNDKISRFDFVQKIEVDGQTITLASGEFQIYKQNHSAVVALQIEKIQDSEHSGKLVNKRQFRISGLGGEHTAFNQLPGGKAEYHGKAFSSDDPNGRLHYSIDFTKKQGYGRIEHLKTLEQNVELAAAELKADEKSHAVILGDTRYNSAEKGSYHLALFGDRAQEIAGSAEVKTVNSIHHIGIAGKQLEHHHHHH

>m001577 Mutant_number:m001577 Master.m0006: m0006 Mutations: YA221L,GA222N,TA249S,GA250V

MGPDSDRLQQRRVAADIGTGLADALTAPLDHKDKGLKSLTLEDSIRQNGTLTLSAQGAEKTFKAGDKDNSLNTGKLKNDKISRFDFVQKIEVDGQTITLASGEFQIYKQNHSAVVALQIEKIQDSEHSGKLVNKRQFRISGLGGEHTAFNQLPGGKAEYHGKAFSSDDPNGRLHYSIDFTKKQGYGRIEHLKTLEQNVELAAAELKADEKSHAVILGDTRLNSAEKGSYHLALFGDRAQEIAGSAEVKSVEGIHHIGIAGKQLEHHHHHH

>m002255 Mutant_number:m002255 Master.m0006: m0006 Mutations: GA222N,SA223Q,GA250V,EA251N,GA252S

MGPDSDRLQQRRVAADIGTGLADALTAPLDHKDKGLKSLTLEDSIRQNGTLTLSAQGAEKTFKAGDKDNSLNTGKLKNDKISRFDFVQKIEVDGQTITLASGEFQIYKQNHSAVVALQIEKIQDSEHSGKLVNKRQFRISGLGGEHTAFNQLPGGKAEYHGKAFSSDDPNGRLHYSIDFTKKQGYGRIEHLKTLEQNVELAAAELKADEKSHAVILGDTRYNQAEKGSYHLALFGDRAQEIAGSAEVKTVNSIHHIGIAGKQLEHHHHHH

>m001176 Mutant_number:m001176 Master.m0006: m0006 Mutations: YA221L,SA223G,TA249S,GA250V,EA251N,GA252S

MGPDSDRLQQRRVAADIGTGLADALTAPLDHKDKGLKSLTLEDSIRQNGTLTLSAQGAEKTFKAGDKDNSLNTGKLKNDKISRFDFVQKIEVDGQTITLASGEFQIYKQNHSAVVALQIEKIQDSEHSGKLVNKRQFRISGLGGEHTAFNQLPGGKAEYHGKAFSSDDPNGRLHYSIDFTKKQGYGRIEHLKTLEQNVELAAAELKADEKSHAVILGDTRLGGAEKGSYHLALFGDRAQEIAGSAEVKSVNSIHHIGIAGKQLEHHHHHH

>m002417 Mutant_number:m002417 Master.m0006: m0006 Mutations: GA222N,SA223G,GA250V,EA251N,GA252S

MGPDSDRLQQRRVAADIGTGLADALTAPLDHKDKGLKSLTLEDSIRQNGTLTLSAQGAEKTFKAGDKDNSLNTGKLKNDKISRFDFVQKIEVDGQTITLASGEFQIYKQNHSAVVALQIEKIQDSEHSGKLVNKRQFRISGLGGEHTAFNQLPGGKAEYHGKAFSSDDPNGRLHYSIDFTKKQGYGRIEHLKTLEQNVELAAAELKADEKSHAVILGDTRYNGAEKGSYHLALFGDRAQEIAGSAEVKTVNSIHHIGIAGKQLEHHHHHH

>m001524 Mutant_number:m001524 Master.m0006: m0006 Mutations: YA221L,GA222N,SA223Q,GA250V,GA252S

MGPDSDRLQQRRVAADIGTGLADALTAPLDHKDKGLKSLTLEDSIRQNGTLTLSAQGAEKTFKAGDKDNSLNTGKLKNDKISRFDFVQKIEVDGQTITLASGEFQIYKQNHSAVVALQIEKIQDSEHSGKLVNKRQFRISGLGGEHTAFNQLPGGKAEYHGKAFSSDDPNGRLHYSIDFTKKQGYGRIEHLKTLEQNVELAAAELKADEKSHAVILGDTRLNQAEKGSYHLALFGDRAQEIAGSAEVKTVESIHHIGIAGKQLEHHHHHH

>m001639 Mutant_number:m001639 Master.m0006: m0006 Mutations: YA221L,GA222N,TA249I,GA250V,EA251V,GA252A

MGPDSDRLQQRRVAADIGTGLADALTAPLDHKDKGLKSLTLEDSIRQNGTLTLSAQGAEKTFKAGDKDNSLNTGKLKNDKISRFDFVQKIEVDGQTITLASGEFQIYKQNHSAVVALQIEKIQDSEHSGKLVNKRQFRISGLGGEHTAFNQLPGGKAEYHGKAFSSDDPNGRLHYSIDFTKKQGYGRIEHLKTLEQNVELAAAELKADEKSHAVILGDTRLNSAEKGSYHLALFGDRAQEIAGSAEVKIVVAIHHIGIAGKQLEHHHHHH

>m002428 Mutant_number:m002428 Master.m0006: m0006 Mutations: GA222N,SA223G,GA250D,EA251V

MGPDSDRLQQRRVAADIGTGLADALTAPLDHKDKGLKSLTLEDSIRQNGTLTLSAQGAEKTFKAGDKDNSLNTGKLKNDKISRFDFVQKIEVDGQTITLASGEFQIYKQNHSAVVALQIEKIQDSEHSGKLVNKRQFRISGLGGEHTAFNQLPGGKAEYHGKAFSSDDPNGRLHYSIDFTKKQGYGRIEHLKTLEQNVELAAAELKADEKSHAVILGDTRYNGAEKGSYHLALFGDRAQEIAGSAEVKTDVGIHHIGIAGKQLEHHHHHH

>m002091 Mutant_number:m002091 Master.m0006: m0006 Mutations: GA222R,GA250V,GA252A

MGPDSDRLQQRRVAADIGTGLADALTAPLDHKDKGLKSLTLEDSIRQNGTLTLSAQGAEKTFKAGDKDNSLNTGKLKNDKISRFDFVQKIEVDGQTITLASGEFQIYKQNHSAVVALQIEKIQDSEHSGKLVNKRQFRISGLGGEHTAFNQLPGGKAEYHGKAFSSDDPNGRLHYSIDFTKKQGYGRIEHLKTLEQNVELAAAELKADEKSHAVILGDTRYRSAEKGSYHLALFGDRAQEIAGSAEVKTVEAIHHIGIAGKQLEHHHHHH

>m001180 Mutant_number:m001180 Master.m0006: m0006 Mutations: YA221L,SA223G,TA249S,GA250V,EA251V,GA252A

MGPDSDRLQQRRVAADIGTGLADALTAPLDHKDKGLKSLTLEDSIRQNGTLTLSAQGAEKTFKAGDKDNSLNTGKLKNDKISRFDFVQKIEVDGQTITLASGEFQIYKQNHSAVVALQIEKIQDSEHSGKLVNKRQFRISGLGGEHTAFNQLPGGKAEYHGKAFSSDDPNGRLHYSIDFTKKQGYGRIEHLKTLEQNVELAAAELKADEKSHAVILGDTRLGGAEKGSYHLALFGDRAQEIAGSAEVKSVVAIHHIGIAGKQLEHHHHHH

>m001941 Mutant_number:m001941 Master.m0006: m0006 Mutations: SA223G,GA250D,EA251N,GA252A

MGPDSDRLQQRRVAADIGTGLADALTAPLDHKDKGLKSLTLEDSIRQNGTLTLSAQGAEKTFKAGDKDNSLNTGKLKNDKISRFDFVQKIEVDGQTITLASGEFQIYKQNHSAVVALQIEKIQDSEHSGKLVNKRQFRISGLGGEHTAFNQLPGGKAEYHGKAFSSDDPNGRLHYSIDFTKKQGYGRIEHLKTLEQNVELAAAELKADEKSHAVILGDTRYGGAEKGSYHLALFGDRAQEIAGSAEVKTDNAIHHIGIAGKQLEHHHHHH

>WT_v3

MGPDSDRLQQRRVAADIGTGLADALTAPLDHKDKGLKSLTLEDSIPQNGTLTLSAQGAEKTFKAGDKDNSLNTGKLKNDKISRFDFVQKIEVDGQTITLASGEFQIYKQNHSAVVALQIEKINNPDKTDSLINQRSFLVSGLGGEHTAFNQLPGGKAEYHGKAFSSDDPNGRLHYSIDFTKKQGYGRIEHLKTLEQNVELAAAELKADEKSHAVILGDTRYGSEEKGTYHLALFGDRAQEIAGSATVKIGEKVHEIGIAGKQLEHHHHHG
