# Supplementary material for: Design of cross-reactive antigens with machine learning and high-throughput experimental evaluation
Source: Front Bioinform. 2025 Jul 16;5:1580967. doi: 10.3389/fbinf.2025.1580967 (PMC12319226; doi:10.3389/fbinf.2025.1580967)
Supplement: Supplementary file 9 [file Table6.docx]

**Table S6: Data reduction and refinement statics.**

|  | **fHbp 2416 + JAR5 Fab** | **fHbp v1.1 + JAR5 Fab** |
| --- | --- | --- |
| PDB Code | 7SA6 | 7SBZ |
| Data Collection Source | APS 22-ID | APS 22-ID |
| Space Group | C2 | I4_1_22 |
| Unit Cell | 148.99 66.36 105.37  90.0 93.429 90.0 | 167.66 167.66 313.16  90.0 90.0 90.0 |
| Resolution (Å) | 19.93 – 2.9 (3.003 – 2.90) | 49.27 – 2.9 (3.004-2.9) |
| Total Reflections | 86303 (8980) | 661600 (67817) |
| Unique Reflections | 22479 (2240) | 49617 (4851) |
| R_merge_ | 0.1362 (1.366) | 0.5835 (4.16) |
| R_meas_ | 0.1582 (1.575) | 0.6071 (4.318) |
| Completeness | 97.46 (99.29) | 99.83 (99.73) |
| Multiplicity | 3.8 (4.0) | 13.3 (14.0) |
| I/σ | 9.15 (1.45) | 9.48 (1.49) |
| CC1/2 | 0.993 (0.486) | 0.996 (0.649) |
|  |  |  |
| Refinement Reflections | 22465 | 49573 |
| R_free_ Reflections | 1126 | 3818 |
| R_work_/R_free_ | 0.2486 / 0.2899 | 0.2474/0.2951 |
| No. of Atoms | 4158 | 10211 |
| Bond Lengths RMS (Å) | 0.0031 | 0.003 |
| Bond Angles RMS (°) | 0.641 | 0.61 |
| Rotamer Outliers (%) | 0.0 | 1.7 |
| Ramachandran Outliers (%) | 0.0 | 0.0 |
| Ramachandran Favored (%) | 97.12 | 95.56 |
| Clashscore | 6.42 | 9.32 |
