# Supplementary material for: Design of cross-reactive antigens with machine learning and high-throughput experimental evaluation
Source: Front Bioinform. 2025 Jul 16;5:1580967. doi: 10.3389/fbinf.2025.1580967 (PMC12319226; doi:10.3389/fbinf.2025.1580967)
Supplement: Supplementary file 11 [file Image1.pdf]

Figure S1

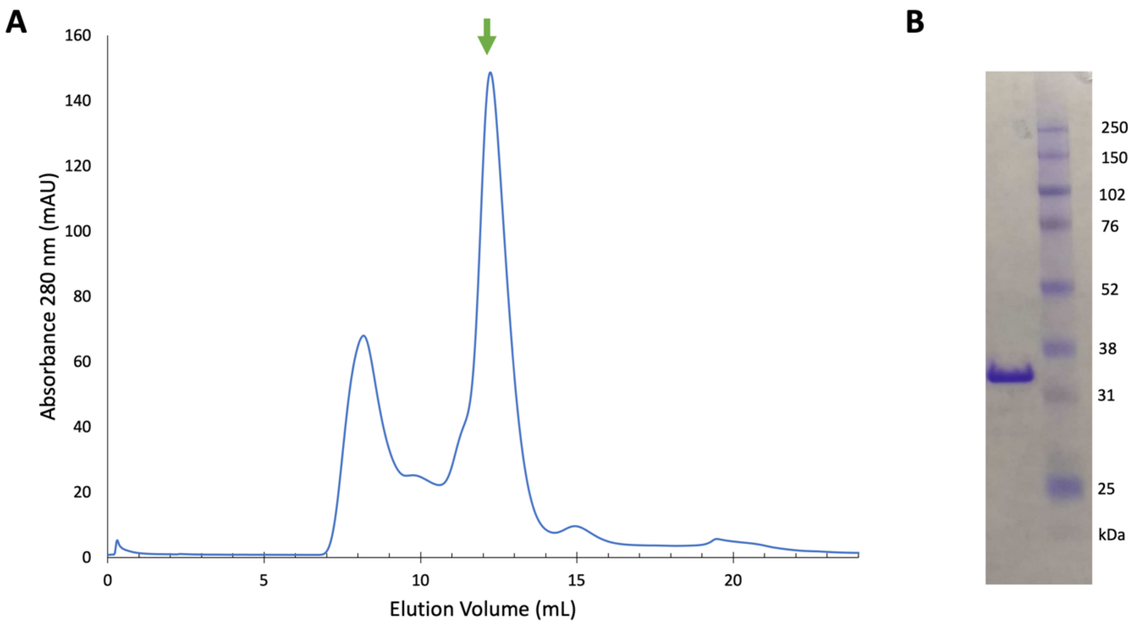

Fig S1: fHbp protein quality. (A) Size exclusion chromatography trace of fHbp m002416 collected during protein purification. (B) SDS-PAGE analysis of purified protein collected from the peak of the SEC trac.
